# Three new species of the spider genus *Plato* and the new genus *Cuacuba* from caves of the states of Pará and Minas Gerais, Brazil (Araneae, Theridiosomatidae)

**DOI:** 10.3897/zookeys.753.20805

**Published:** 2018-04-27

**Authors:** Pedro H. Prete, Igor Cizauskas, Antonio D. Brescovit

**Affiliations:** 1 Laboratório Especial de Coleções Zoológicas, Instituto Butantan. Av. Vital Brazil, 1500, CEP 05503-900, São Paulo, SP, Brazil

**Keywords:** biospeleology, Neotropical region, taxonomy

## Abstract

Three new species of the genus *Plato* from caves in the states of Pará and Minas Gerais, Brazil, are described. *P. novalima* **sp. n.**, from Minas Gerais, is the first record of the genus in the southeastern region of Brazil. *P. ferriferus* **sp. n.** and *P. striatus* **sp. n.**, from Carajás, Pará, north of Brazil, are also described. The former is an extremely abundant species, whereas the latter has only one known male specimen. *Cuacuba* **gen. n.** is proposed and represented by two new species, *C. mariana* **sp. n.** (type species) and *C. morrodopilar* **sp. n.**, both from the state of Minas Gerais. Morphology of genitalia in *Cuacuba* **gen. n.** is similar to other Theridiosomatidae genera and is herein discussed. None of the proposed species presents troglomorphic adaptations. They are widespread, abundant inside caves in different and large karst areas, and each genus prefers different lithologies.

## Introduction

The family Theridiosomatidae Simon 1881 is currently represented by 18 genera and 111 species worldwide (World Spider Catalog 2017). Specimens of this family differ from others by the presence of pits on the anterior margin of the sternum (except in *Chthonos* Coddington, 1986), connate spermathecae in female genitalia (except in *Coddingtonia* Miller, Griswold & Yin, 2009 and *Tagalogonia* Labarque & Griswold, 2014) and tibial dorsal trichobothria, usually 2 to 4 times longer than tibial diameter (shorter only in *Wendilgarda* Keyserling, 1886) (Coddington 1986).

Theridiosomatidae is divided into four subfamilies (Coddington 1986), which include the following Neotropical genera: Epeirotypinae Coddington, 1986, with *Epeirotypus* O. Pickard-Cambridge, 1894 and *Naatlo* Coddington, 1986; Ogulninae Coddington, 1986, only with *Ogulnius* O. Pickard-Cambridge, 1882; Theridiosomatinae Coddington, 1986, with *Theridiosoma* O. Pickard-Cambridge, 1879, *Baalzebub* Coddington, 1986, *Epilineutes* Coddington, 1986 and *Wendilgarda*; Platoninae Coddington, 1986, with *Chthonos* and *Plato* Coddington, 1986. Representatives of the subfamily Platoninae differ from other Theridiosomatidae due to the following characteristics: cubic eggsacs suspended from a single long wire; male palp with paracymbium T-shaped; margin of cymbium and distal alveoli punctuated or bifid medially; medial apophysis with a long and curved tip; and conductor with a thick ventral apophysis (Coddington 1986).

The diagnostic characteristics that differentiate *Plato* from other genera in Theridiosomatidae are the presence of a median projection in the female epigynum base, grooves in the distal or mesal area of the cymbium, and a strong, curved ventral apophysis in the conductor of male palp (Coddington 1986). *Plato* includes six species: the type species *Plato troglodita* Coddington 1986, from Ecuador; *Plato bicolor* (Keyserling 1986), from the Amazonian region in Brazil; *Plato bruneti* (Gertsch 1960), from Trinidad and Tobago; *Plato guacharo* and *Plato miranda*, both described by Brignoli (1972), from Venezuela; and *Plato juberthiei* Lopez 1996, from French Guiana (World Spider Catalog 2017). Their distribution is currently limited to South America and, so far, only *P. bicolor* has been described from Brazil. Specimens of *Plato* are found in caves, near stream passages, or on the wall, next to water bodies, preying on small flying insects (Fig. 3B; Trajano and Bichuette 2010).

Two species were collected from the Carajás region, *Plato ferriferus* sp. n. and *Plato striatus* sp. n. *Plato novalima* sp. n. was collected in the Iron Quadrangle region and surrounding cave areas in the state of Minas Gerais. Besides *Plato*, other Theridiosomatidae genera are also commonly found inside caves, such as *Baalzebub*, also from the state of Minas Gerais (Prete et al. 2016), and *Wendilgarda* (Trajano 1987; Gnaspini and Trajano 1994) and *Epilineutes* (Gnaspini and Trajano 1994), both from the state of São Paulo.

Furthermore, two new species are described and included in the newly proposed genus, *Cuacuba* gen. n. Specimens of this genus were recorded in the literature as unidentified *Plato* sp. from the caves Lapa Encantada (Pinto-da-Rocha 1995) and Morena (Gnaspini and Trajano 1994), both in the state of Minas Gerais. While reexamining the material currently deposited in the IBSP collection, we detected diagnostic characters, mainly in the genital structures, that allow us to propose a new genus to include the type species *Cuacuba mariana* sp. n. and *Cuacuba morrodopilar* sp. n., both from caves of Iron Quadrangle formation. The species of this genus have a set of characteristics that do not place them into any Theridiosomatidae genus. Males are diagnosed by having a bifid conductor with two long apophyses: the posterior one involving a long embolus and the anterior one distally ornamented. Females present an epigynal plate with a projected posterior margin. Relationships among the new genus and other Theridiosomatidae still need to be evaluated phylogenetically as the morphological characters of genitalia in this genus do not allow it to be placed within any of the current subfamilies.

## Materials and methods

A total of 3868 adult specimens of five species from 1007 different caves was analyzed. The examined material was collected between 1984 and 2015 and deposited in the following taxonomic collections (curators between parentheses): IBSP, Instituto Butantan, São Paulo (A.D. Brescovit); ISLA, Coleção de Invertebrados Subterrâneos de Lavras, Universidade Federal de Lavras, Lavras (R.L. Ferreira); MPEG, Museu Paraense Emilio Goeldi, Belém (A.B. Bonaldo); MZSP, Museu de Zoologia da Universidade de São Paulo, São Paulo (R. Pinto da Rocha).

Spiders were collected in different karst areas throughout the states of Minas Gerais and Pará in Brazil. Iron and carbonate formations are the most representative among the explored areas. All samples were manually collected.

The material was studied using a stereomicroscope Nikon SMZ 745T. Multifocal photos were taken using a stereomicroscope Leica DFC500 and the Leica Application Suite v3.3.0 software. Scanning Electron Microscopy (SEM) images of the left palp of males (as is standard in arachnological studies) were made using a FEI Quanta 250 microscope from Laboratório de Biologia Celular of Instituto Butantan.

Genitalia were dissected and illustrations were made using a Leica MZ12 stereomicroscope with an attached camera lucida. Descriptions of species follow Coddington (1986). All measurements are presented in millimeters (mm). Maps were produced using GPS Track Maker-PRO and edited in GIMP v2.8.14 and Inkscape v0.48.4. Graphics and tables were made in Microsoft Office Excel 2013.

Abbreviations:

**ALE** anterior lateral eyes,

**AME** anterior median eyes,

**MF** multifocal photo

**PLE** posterior lateral eyes,

**PME** posterior median eyes,

**SEM** scanning electron microscopy.

### Collecting sites

Five new species of Theridiosomatidae were found inside caves in the states of Pará and Minas Gerais, Brazil. The species’ distributions are mainly restricted to two karst areas of great economic and mineral interest. One of them is the region of Carajás, state of Pará, an important speleological area in Brazil. Its iron formation has a great potential for forming cavities, as it concentrates one of the largest iron reserves in the world. These caves have a singular biological evolution (Ab’Sáber 1986; Piló and Auler 2009), in which a series of discontinuous mountains and hills are separated by wide valleys of iron ore that emerge above the forested plateau with elevations of 600 to 800 m. The main elevations are the northern and southern mountains (Serra Norte e Serra Sul) (Cunha Jr. et al. 2007).

The second area is in the Iron Quadrangle, located in the central part of the state of Minas Gerais, and covers approximately 7,200 km^2^. During the evolution of the area`s relief, small erosion surfaces were formed, distributed at different altitudes and with a clear lithostructural control. This region is a unique landscape when compared to the other southeastern geological patterns in Brazil (Varajão 1991).

## Taxonomic part

### Family Theridiosomatidae

#### Subfamily Platoninae

##### Genus Plato

Taxon classificationAnimaliaAraneaeTheridiosomatidae

Coddington, 1986

###### Type species.

*Plato troglodita* Coddington, 1986, by original designation.

##### Plato ferriferus sp. n.

Taxon classificationAnimaliaAraneaeTheridiosomatidae

http://zoobank.org/3C5D2399-EB83-4572-964A-6EF7058C4C9C

**Figure 1.**
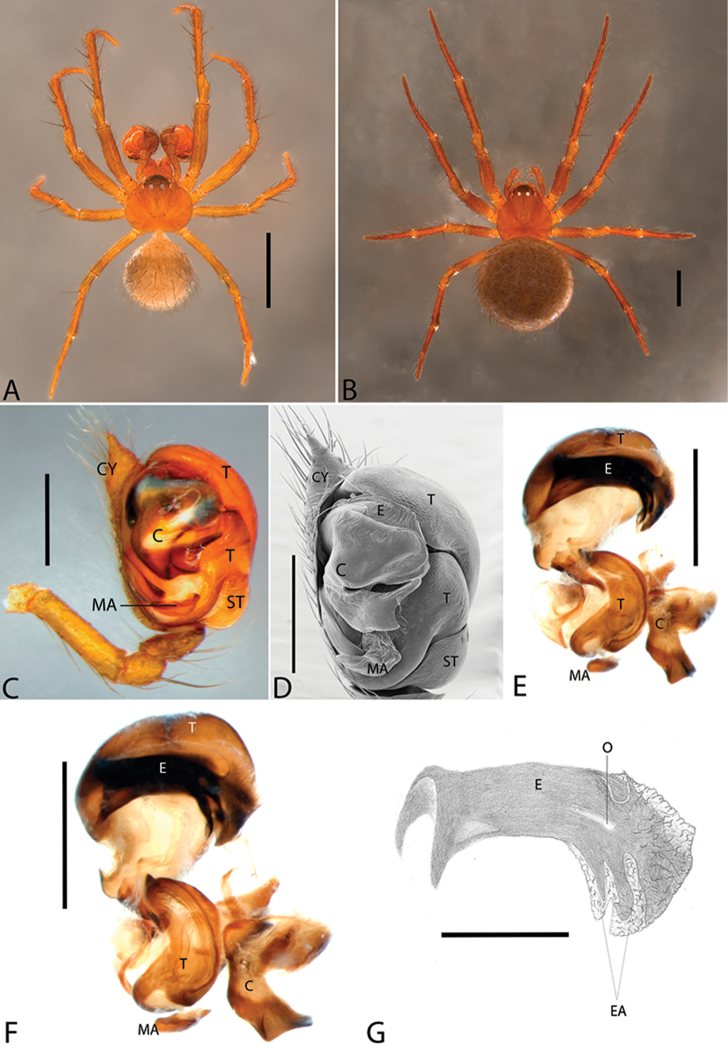
*Plato ferriferus* sp. n. **A** male, dorsal view **B** female, dorsal view **C** male palp, ventral view **D** male palp, ventral view (SEM) **E–F** male palp, ventral view, expanded and with conductor removed **G** embolus, illustration. Abbreviations: **C** conductor; **CY** cymbium; **E** embolus; **EA** mesal embolic apophysis; **MA** median apophysis; **O** embolic opening; **ST** subtegulum; **T** tegulum. Scale bars: **A** 1.1; **B** 1; **C–F** 0.3; **G** 0.15 mm.

**Figure 2.**
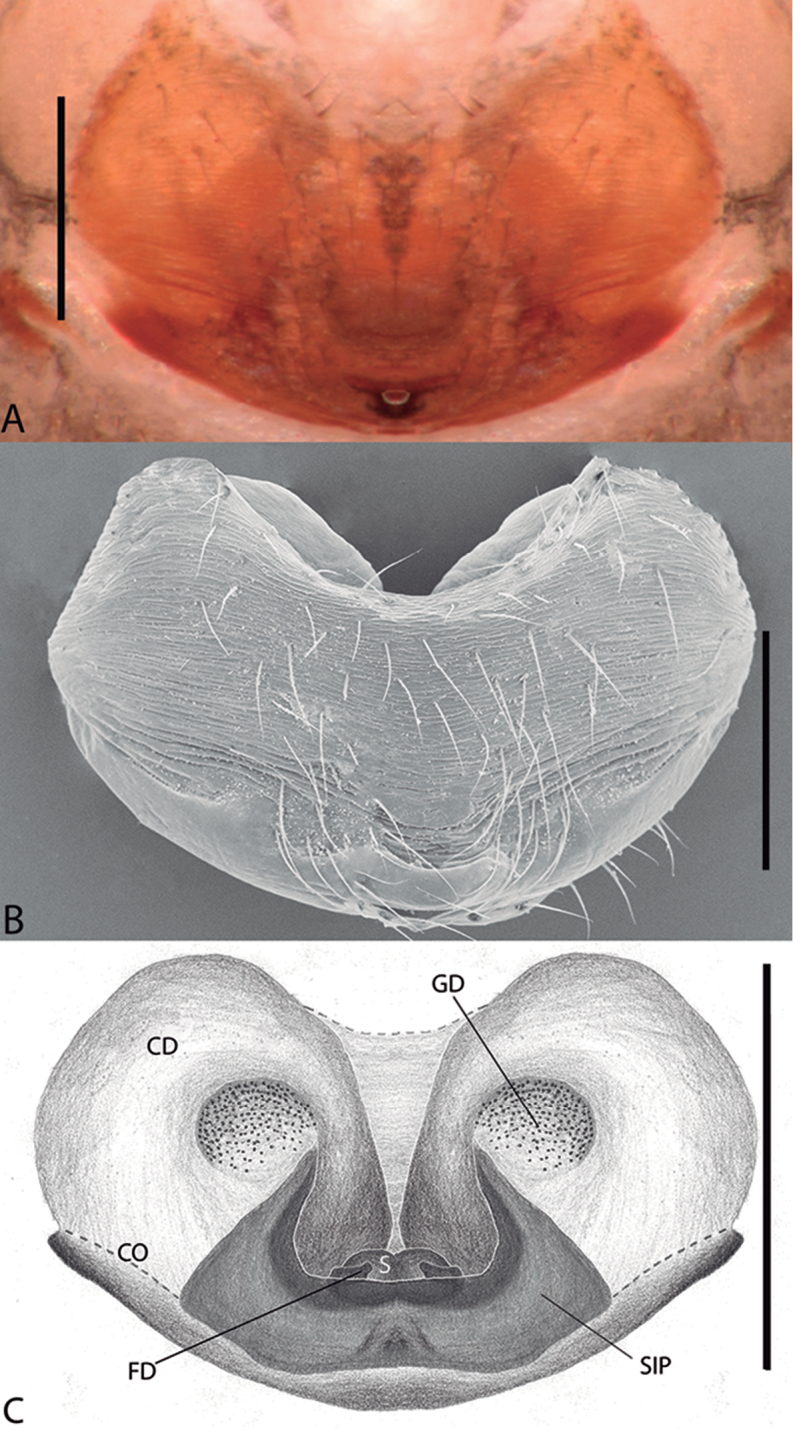
*Plato ferriferus* sp. n. **A** female epigynum, ventral view (LM) **B** female epigynum, ventral view (SEM) **C** female epigynum, dorsal view (drawing). Abbreviations: **CD** copulatory ducts; **CO** copulatory opening; **FD** fertilization ducts; **GD** glandular ducts; **S** spermathecae; **SIP** sclerotized internal plate. Scale bars: **A** 0.17; **B** 0.2; **C** 0.5 mm.

**Figure 3.**
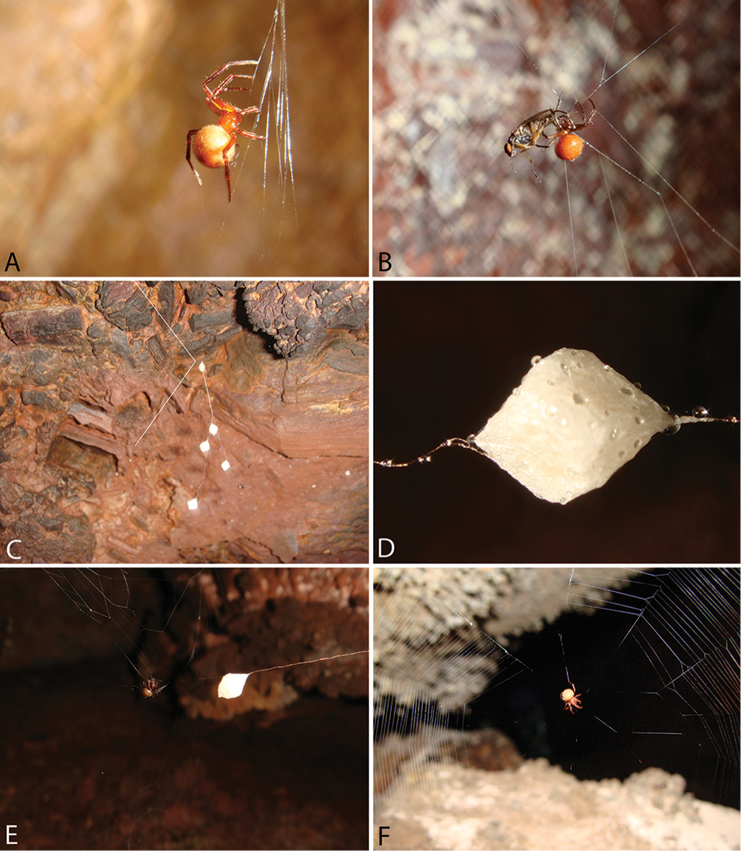
**A** *Plato ferriferus* sp. n. on its web **B** *Plato ferriferus* sp. n. preying on fly **C, D** *Plato ferriferus* sp. n. egg sacs **E** *Plato novalima* sp. n. on its web, building its egg sac **F** *Cuacuba mariana* sp. n. on its web; Photos **A–D** were taken in caves in Parauapebas, PA, Brazil. Photo E was taken in Mariana, Minas Gerais, Brazil. Photo **F** was taken in Matozinhos, Minas Gerais, Brazil. (I. Cizauskas).

Figures 1 , 2 , 3 , 16

###### Types.

Male holotype and female paratype from Cave N4E_0079 (6°02'00"S, 50°09'07"W), Parauapebas, Pará, Brazil, 19/II–4/III/2010, deposited in IBSP 173283. Paratypes: male and female from Cave N5S_0037 (6°06'23"S, 50°07'59"W), 15–21/IX/2009 (MPEG 32028); male and female from Cave N5S_0012 (6°06'13"S, 50°07'33"W), 14–23/X/2009 (MPEG 32027); male and female from Cave N5S_0030 (6°05'20"S, 50°07'12"W), 14–16/XII/2010 (MZSP 70929); male and female from Cave N5S_0028 (6°05'19"S, 50°07'33"W), 10–19/V/2011 (MZSP 70930), all from Parauapebas, Pará, Brazil, R. Andrade & I. Cizauskas et al. coll.

###### Other material examined.

BRAZIL. **Pará**: Parauapebas, FLONA de Carajás, Cave NV_01 (6°28'36"S, 49°54'13"W), 1♀ (IBSP 55373); Cave NV_03 (6°28'43"S, 49°54'10"W), 1♀ (IBSP 55385); Cave NV_05 (6°28'43"S, 49°54'9"W), 1♀ (IBSP 55369); Cave NV_06 (6°28'48"S, 49°54'26"W), 1♀ (IBSP 55380); Cave NV_07 (6°28'41"S, 49°54'20"W), 1♀ (IBSP 55386), all collected on 22–28/II/2005 by R. Andrade et al.; Cave N5E_0001 (6°4'25"S, 50°7'5"W), 1♀ (IBSP 55786); Cave N5E_0008 (6°4'54"S, 50°7'50"W), 2♀ (IBSP 55379); Cave N5E_0009 (6°4'54"S, 50°7'43"W), 1♀ (IBSP 55383); Cave N5E_0010 (6°4'50"S, 50°7'42"W), 2♀ (IBSP 55377); Cave N5E_0011 1♀ (IBSP 55374); Cave N5E_0013, 1♀ (IBSP 55368); Cave N5E_0014, 2♀ (IBSP 55370); Cave N5E_0015 1♀ (IBSP 55378); Cave N5E_0016 2♀ (IBSP 55375), all collected on 22/III–03/IV/2005 by R. Andrade et al.; Cave N5E_0004 (6°5'11"S, 50°7'47"W), 1♂ 2♀, 03–13/V/2005 (IBSP 55376), collected by R. Andrade et al.; Cave N4E_0017 (6°2'7"S, 50°9'37"W), 2♀ (IBSP 174998); Cave N4E_0027 (6°2'15"S, 50°10'4"W), 1♀ (IBSP 175039); Cave N4E_0028 (6°2'15"S, 50°10'3"W), 2♀ (IBSP 175040); Cave N4E_0030 (6°2'25"S, 50°9'41"W), 3♀ (IBSP 175041); Cave N4E_0032 (6°2'24"S, 50°9'40"W), 1♂ 1♀, (IBSP 175049); Cave N4E_0034 (6°2'24"S, 50°9'39"W), 2♀ (IBSP 175065); Cave N4E_0035 (6°2'20"S, 50°9'39"W), 1♂ 2♀ (IBSP 175067); Cave N4E_0036 (6°2'10"S, 50°9'37"W), 1♀ (IBSP 175071); 1♀, 18/VIII–03/IX/2009 (IBSP 173197); Cave N4E_0038 (6°2'6"S, 50°9'39"W), 1♀ (IBSP 175073); Cave N4E_0040 (6°2'1"S, 50°9'45"W), 2♀ (IBSP 175074); Cave N4E_0041 (6°2'0"S, 50°9'43"W), 3♀ (IBSP 175076); 3♀, 24–30/VII/2009 (IBSP 173209); Cave N4E_0061 (6°2'22"S, 50°10'5"W), 1♂ 3♀ (IBSP 175078); 7♀, 24–30/VII/2009 (IBSP 173216, IBSP 173217, IBSP 173218, IBSP 173219), all collected by R. Andrade et al.; Cave N4E_0010 (6°2'22"S, 50°9'40"W), 2♀ (IBSP 174967, IBSP 174971), 1♂ 1♀, 20/IV–04/V/2010 (IBSP 173323, IBSP 173324), 3♀, 20/X–01/XI/2006 (IBSP 174966); Cave N4E_0022 (6°2'3"S, 50°10'5"W) 1♀ (IBSP 175017); 7♀, 20/IV–04/V/2010 (IBSP 173344–IBSP 173347); 1♂ 9♀, 20/X–01/XI/2006 (IBSP 175013); Cave N4E_0026 (6°2'16"S, 50°10'4"W), 7♀ (IBSP 175027); 1♂ 19♀, 08–12/II/2007 (IBSP 175026); 2♂ 16♀, 18/VIII–03/IX/2009 (IBSP 173187–IBSP 173192); Cave N4E_0033 (6°2'26"S, 50°9'38"W), 1♂ 3♀ (IBSP 175059); 1♂ 15♀, 08–12/II/2007 (IBSP 175054); 2♂ 7♀, 15–22/IX/2009 (IBSP 173200–IBSP 173203); Cave N4E_0068 (6°1'56"S, 50°9'4"W), 3♂ 2♀ (IBSP 175081); 3♀, 19/II–04/III/2010 (IBSP 173252, IBSP 173253); 1♂ 2♀, 24–30/VII/2009 (IBSP 173146, IBSP 173147); Cave N4E_0092 (6°2'24"S, 50°9'32"W), 3♀ (IBSP 175082); 2♂ 5♀, 19/II–04/III/2010 (IBSP 173297–IBSP 173300); 11♀, 24–30/VII/2009 (IBSP 173180–IBSP 173184); Cave N4E_0095 (6°2'25"S, 50°9'33"W), 1♂ 6♀ (IBSP 175085, IBSP 175086); 2♂ 7♀, 19/II–04/III/2010 (IBSP 173303–IBSP 173305); 1♂ 13♀, 24–30/VII/2009 (IBSP 173166–IBSP 173169) all collected by R. Andrade et al.; Cave N4E_0039 (6°1'60"S, 50°9'40"W), 1♂ 3♀ (IBSP 173206–IBSP 173208); Cave N4E_0062 (6°2'2"S, 50°9'14"W), 1♀ (IBSP 173138); Cave N4E_0082 (6°2'0"S, 50°9'23"W), 4♀ (IBSP 173143, IBSP 173144) all collected on 24–30/VII/2009 by R. Andrade et al.; Cave N4E_0031 (6°2'26"S, 50°9'41"W), 1♂ 1♀ (IBSP 173195); Cave N4E_0085 (6°2'4"S, 50°9'27"W), 4♀ (IBSP 173193, IBSP 173194); 1♀, 19/II–04/III/2010 (IBSP 173289); Cave N4E_0087 (6°1'58"S, 50°9'2"W), 1♀ (IBSP 173185, IBSP 173186); 1♀, 20/IV–04/V/2010 (IBSP 173290, IBSP 173291) all collected by R. Andrade et al.; Cave N4E_0075 (6°1'54"S, 50°9'3"W), 1♂ 5♀ (IBSP 173204, IBSP 173205); 2♂ 6♀, 19/II–04/III/2010 (IBSP 173274, IBSP 173275, IBSP 173276, IBSP 173277) collected by I. Cizauskas; Cave N4E_0043 (6°1'56"S, 50°9'52"W), 2♀ (IBSP 173220); Cave N4E_0044 (6°1'56"S, 50°9'51"W), 1♂ 3♀ (IBSP 173221, IBSP 173222); 2♀, 24–30/VII/2009 (IBSP 173210, IBSP 173211); Cave N4E_0045 (6°2'27"S, 50°9'42"W), 2♀ (IBSP 173224); Cave N4E_0046 (6°2'17"S, 50°9'38"W), 1♂ 2♀ (IBSP 173232–IBSP 173234); Cave N4E_0048 (6°2'17"S, 50°9'38"W), 2♀ (IBSP 173235, IBSP 173239); Cave N4E_0050 (6°2'11"S, 50°9'37"W), 1♀ (IBSP 173225); Cave N4E_0051 (6°2'24"S, 50°9'40"W), 2♀ (IBSP 173236); 1♂ 1♀, 24–30/VII/2009 (IBSP 173212); Cave N4E_0054 (6°2'2"S, 50°10'9"W), 2♀ (IBSP 173226, IBSP 173227); Cave N4E_0055 (6°1'56"S, 50°10'1"W), 1♀ (IBSP 173229); 1♀ 24–30/VII/2009 (IBSP 173213); Cave N4E_0057 (6°1'57"S, 50°9'50"W), 3♀ (IBSP 173230, IBSP 173231); 1♂ (IBSP 173214); Cave N4E_0059 (6°2'13"S, 50°10'5"W), 1♂ 1♀ (IBSP 173237, IBSP 173238); Cave N4E_0060 (6°2'13"S, 50°10'6"W), 1♀ (IBSP 173228); 1♀ (IBSP 173215); Cave N4E_0063 (6°2'3"S, 50°9'17"W), 2♀ (IBSP 173223); Cave N4E_0065 (6°1'59"S, 50°9'6"W), 3♀ (IBSP 173243, IBSP 173244); Cave N4E_0066 (6°1'53"S, 50°9'5"W), 1♂ 2♀ (IBSP 173245, IBSP 173246); Cave N4E_0067 (6°1'56"S, 50°9'4"W), 6♀ (IBSP 173247–IBSP 173250); 2♂ 3♀ (IBSP 173139, IBSP 173140); Cave N4E_0069 (6°1'56"S, 50°9'11"W), 3♀ (IBSP 173254); 4♀ (IBSP 173155–IBSP 173158); Cave N4E_0070 (6°1'57"S, 50°9'12"W), 1♂ 5♀ (IBSP 173255–IBSP 173258); 3♂ 15♀ (IBSP 173148–IBSP 173152); Cave N4E_0071 (6°1'58"S, 50°9'14"W), 1♀ (IBSP 173259); 1♂ 5♀ (IBSP 173141, IBSP 173142); Cave N4E_0072 (6°1'58"S, 50°9'14"W), 7♀ (IBSP 173260–IBSP 173263); 9♀ (IBSP 173162–IBSP 173164); Cave N4E_0073 (6°1'58"S, 50°9'15"W), 13♀ (IBSP 173264–IBSP 173268, IBSP 173273); 2♂ 3♀ (IBSP 173175–IBSP 173179); Cave N4E_0074 (6°2'0"S, 50°9'22"W), 12♀ (IBSP 173269–IBSP 173272); 7♀ (IBSP 173133–IBSP 173137); Cave N4E_0076 (6°2'1"S, 50°9'5"W), 2♀ (IBSP 173278, IBSP 173279); Cave N4E_0077 (6°1'59"S, 50°9'4"W), 1♂ 1♀ (IBSP 173280, IBSP 173281); 3♀ (IBSP 173131, IBSP 173132); Cave N4E_0078 (6°1'59"S, 50°9'6"W), 2♀ (IBSP 173282); 3♀ (IBSP 173159–IBSP 173161); Cave N4E_0079 (6°1'60"S, 50°9'7"W), 3♀ (IBSP 173284–IBSP 173286); 1♀ (IBSP 173165); Cave N4E_0081 (6°2'0"S, 50°9'22"W), 2♂ 4♀ (IBSP 173240–IBSP 173242); 2♂ 1♀ (IBSP 173170, IBSP 173171); Cave N4E_0083 (6°2'0"S, 50°9'24"W), 1♀ (IBSP 173251); Cave N4E_0084 (6°2'2"S, 50°9'15"W), 2♀ (IBSP 173287, IBSP 173288); 2♀ (IBSP 173145); Cave N4E_0088 (6°1'56"S, 50°9'4"W), 4♀ (IBSP 173290, IBSP 173291); 1♂ 4♀ (IBSP 173185, IBSP 173186); Cave N4E_0089 (6°2'0"S, 50°9'8"W), 2♂ 5♀ (IBSP 173292, IBSP 173293, IBSP 173294); 2♀ (IBSP 173153, IBSP 173154); Cave N4E_0090 (6°2'2"S, 50°9'6"W), 1♀ (IBSP 173295); Cave N4E_0091 (6°2'2"S, 50°9'16"W), 2♀ (IBSP 173296); Cave N4E_0093 (6°2'24"S, 50°9'32"W), 5♀ (IBSP 173301 IBSP 173302); 1♂ 6♀ (IBSP 173172–IBSP 173174) all collected on 24–30/VII/2009 by R. Andrade et al.; Cave N4E_0064 (6°1'57"S, 50°9'5"W), 1♂ 3♀ (IBSP 173306, IBSP 174319, IBSP 177221, IBSP 184741); 1♂ 1♀, 18/VIII–03/IX/2009 (IBSP 173198); Cave N4E_0086 (6°2'7"S, 50°9'39"W), 1♀ (IBSP 173307); 3♀, 18/VIII–03/IX/2009 (IBSP 173196) all collected on 14/III–04/IV/2010 by R. Andrade et al.; Cave N4E_0001 (6°2'26"S, 50°9'41"W), 3♀ (IBSP 173308, IBSP 173309); 1♂ 8♀ (IBSP 174935); Cave N4E_0002 (6°2'26"S, 50°9'40"W), 2♂ 2♀ (IBSP 173310, IBSP 177222, IBSP 184738, IBSP 174938); Cave N4E_0003 (6°2'26"S, 50°9'40"W), 1♀ (IBSP 173311); 2♀ (IBSP 174943); Cave N4E_0004 (6°2'27"S, 50°9'41"W), 2♀ (IBSP 173312); Cave N4E_0005 (6°2'24"S, 50°9'39"W), 1♂ 4♀ (IBSP 173313, IBSP 174317); 5♀ (IBSP 174948); Cave N4E_0006 (6°2'23"S, 50°9'37"W), 2♂ (IBSP 177223, IBSP 184740); Cave N4E_0007 (6°2'22"S, 50°9'37"W), 1♀ (IBSP 173314); 1♀ (IBSP 174951); Cave N4E_0008 (6°2'22"S, 50°9'37"W), 1♂ 3♀ (IBSP 173318–IBSP 173320); 1♂ 3♀ (IBSP 174963); Cave N4E_0009 (6°2'23"S, 50°9'38"W), 1♀ (IBSP 173322); Cave N4E_0011 (6°2'21"S, 50°9'40"W), 2♀ (IBSP 173325, IBSP 173326); 1♂ (IBSP 174973); Cave N4E_0012 (6°2'18"S, 50°9'39"W), 2♀ (IBSP 173327, IBSP 174318); Cave N4E_0013 (6°2'19"S, 50°9'39"W), 3♀ (IBSP 173328, IBSP 173329); 1♀ (IBSP 174977); Cave N4E_0014 (6°2'19"S, 50°9'39"W), 3♀ (IBSP 173330–IBSP 173332); 1♂ 2♀ (IBSP 174988); Cave N4E_0015 (6°2'11"S, 50°9'37"W), 4♀ (IBSP 173315, IBSP 173316, IBSP 173317); 2♀ (IBSP 174992); Cave N4E_0016 (6°2'7"S, 50°9'39"W), 2♀ (IBSP 173333, IBSP 173334); 1♂ (IBSP 174995); Cave N4E_0018 (6°2'6"S, 50°9'39"W), 1♂ (IBSP 177224, IBSP 184739); Cave N4E_0019 (6°2'5"S, 50°9'39"W), 1♀ (IBSP 173335); 5♀ (IBSP 175002); Cave N4E_0020 (6°2'3"S, 50°9'37"W), 1♂ 1♀ (IBSP 173336, IBSP 173337); Cave N4E_0021 (6°2'3"S, 50°9'38"W), 2♂ 6♀ (IBSP 173338–IBSP 173343); 4♀ (IBSP 175009); Cave N4E_0023 (6°2'3"S, 50°10'9"W), 1♂ 4♀ (IBSP 173348–IBSP 173350); 1♂ 3♀ (IBSP 175022); Cave N4E_0025 (6°2'3"S, 50°10'9"W), 1♀ (IBSP 173321), all collected on 20/X–01/XI/2006 by R. Andrade et al.; Cave N5S_0008 (6°6'21"S, 50°7'57"W), 2♀ (IBSP 174522, IBSP 174525); 3♂ 14♀, 14–23/X/2009 (IBSP 172714–IBSP 172718, IBSP 174502); Cave N5S_0010 (6°6'21"S, 50°7'53"W), 2♀ (IBSP 174527); 13♀, 14–23/X/2009 (IBSP 172722–IBSP 172728); Cave N5S_0011 (6°6'18"S, 50°7'47"W), 1♂ 1♀ (IBSP 174530); 1♂ 19♀, 14–23/X/2009 (IBSP 172729–IBSP 172736); Cave N5S_0021 (6°5'15"S, 50°7'34"W), 4♀ (IBSP 174535, IBSP 174539, IBSP 174540); 1♂ 9♀, 25/VIII–03/IX/2009 (IBSP 172659–IBSP 172664), all collected by R. Andrade et al.; Cave N5S_0050 (6°6'26"S, 50°8'1"W), 1♂ 1♀ (IBSP 172652); 1♀, 14/III-04/IV/2010 (IBSP 172769); Cave N5S_0017 (6°5'17"S, 50°7'12"W), 6♀ (IBSP 172665, IBSP 172666); Cave N5S_0018 (6°5'11"S, 50°7'39"W), 1♀ (IBSP 172673); Cave N5S_0019 (6°5'13"S, 50°7'37"W), 4♀ (IBSP 172671, IBSP 172672); Cave N5S_0020 (6°5'16"S, 50°7'37"W), 8♀ (IBSP 172654, IBSP 172655, IBSP 172656, IBSP 172658); Cave N5S_0022 (6°5'16"S, 50°7'33"W), 2♀ (IBSP 172643); Cave N5S_0047 (6°6'26"S, 50°8'2"W), 1♀ (IBSP 172642); Cave N5S_0059 (6°6'29"S, 50°7'58"W), 1♂ 4♀ (IBSP 172641, IBSP 172763, IBSP 172790); Cave N5S_0069 (6°6'4"S, 50°8'8"W), 2♀ (IBSP 172674), all collected on 25/VIII–03/IX/2009 by R. Andrade et al.; Cave N5S_0039 (6°6'21"S, 50°8'2"W), 1♀ (IBSP 174503); Cave N5S_0040 (6°6'20"S, 50°8'2"W), 1♀ (IBSP 172756); Cave N5S_0061 (6°6'20"S, 50°8'5"W), 6♀ (IBSP 172684, IBSP 172791, IBSP 172792); Cave N5S_0062 (6°6'19"S, 50°8'8"W), 1♀ (IBSP 172685); Cave N5S_0066 (6°6'13"S, 50°8'9"W), 5♀ (IBSP 172690, IBSP 174504); Cave N5S_0080 (6°6'9"S, 50°8'15"W), 2♂ 3♀ (IBSP 172688, IBSP 172689, IBSP 174505), all collected on 15–21/IX/2009 by R. Andrade & I. Cizauskas et al.; Cave N5S_0001 (6°5'27"S, 50°7'31"W), 1♀ (IBSP 172737); Cave N5S_0002 (6°5'33"S, 50°7'33"W), 3♀ (IBSP 172748); Cave N5S_0003 (6°6'18"S, 50°8'4"W), 3♀ (IBSP 172708–IBSP 172710); Cave N5S_0004 (6°6'21"S, 50°8'4"W), 3♀ (IBSP 172705–IBSP 172707); Cave N5S_0005 (6°6'21"S, 50°8'1"W), 1♀ (IBSP 172702); Cave N5S_0006 (6°6'21"S, 50°8'2"W), 3♂ 2♀, (IBSP 172703, IBSP 172704); 1♂ 5♀, 15–21/IX/2009 (IBSP 172691–IBSP 172694); Cave N5S_0007 (6°6'22"S, 50°8'1"W), 1♀ (IBSP 172701); Cave N5S_0009 (6°6'22"S, 50°7'53"W), 6♀ (IBSP 172749–IBSP 172752); Cave N5S_0012 (6°6'13"S, 50°7'33"W), 15♀ (IBSP 172738–IBSP 172743, IBSP 172745–IBSP 172747); Cave N5S_0013 (6°6'19"S, 50°8'2"W), 4♀ (IBSP 172711–IBSP 172713); Cave N5S_0014 (6°6'21"S, 50°8'2"W), 2♀ (IBSP 172720, IBSP 172721); Cave N5S_0016 (6°6'21"S, 50°8'1"W), 2♀ (IBSP 172719), all collected on 10–19/V/2011 by R. Andrade et al.; Cave N5S_0036 (6°6'24"S, 50°7'54"W), 1♀ (IBSP 172753); Cave N5S_0037 (6°6'23"S, 50°7'59"W), 3♀ (IBSP 172754); 2♀, 15–21/IX/2009 (IBSP 172755); Cave N5S_0041 (6°6'20"S, 50°8'3"W), 3♀ (IBSP 172758, IBSP 172759); 1♀, 15–21/IX/2009 (IBSP 172757); Cave N5S_0042 (6°6'22"S, 50°8'4"W), 4♀, 25/VIII–03/IX/2009 (IBSP 172760); 1♀ (IBSP 172761); Cave N5S_0044 (6°6'26"S, 50°8'3"W), 1♂ 1♀ (IBSP 172778); 1♀ (IBSP 172777); Cave N5S_0045 (6°6'25"S, 50°8'2"W), 2♀ (IBSP 172762, IBSP 172764); 3♀ (IBSP 172679); Cave N5S_0049 (6°6'27"S, 50°8'1"W), 3♀ (IBSP 172765, IBSP 172767 IBSP 172768); 2♀ (IBSP 172678, IBSP 172766); Cave N5S_0051 (6°6'28"S, 50°8'1"W), 2♀ (IBSP 172776); 1♀ (IBSP 172775); Cave N5S_0052 (6°6'29"S, 50°7'60"W), 7♀ (IBSP 172770–IBSP 172774); 1♀ (IBSP 172677); Cave N5S_0054 (6°6'29"S, 50°8'0"W), 1♂ (IBSP 172780); 2♀, 25/VIII–03/IX/2009 (IBSP 172779); Cave N5S_0055 (6°6'29"S, 50°7'58"W), 1♀ (IBSP 172781); 2♂ 1♀ (IBSP 172653, IBSP 172782); Cave N5S_0056 (6°6'29"S, 50°7'59"W), 2♀ (IBSP 172784, IBSP 172785); 1♂ 1♀ (IBSP 172636, IBSP 172783); Cave N5S_0057 (6°6'29"S, 50°7'59"W), 2♂ 5♀ (IBSP 172786, IBSP 172787); 1♂ 9♀ (IBSP 172637– IBSP 172640); Cave N5S_0058 (6°6'29"S, 50°7'59"W), 1♂ 4♀ (IBSP 172788, IBSP 172789); Cave N5S_0063 (6°6'14"S, 50°8'9"W), 13♀ (IBSP 172793–IBSP 172802); 1♂ 10♀, 15–21/IX/2009 (IBSP 172695–IBSP 172700); Cave N5S_0068 (6°6'5"S, 50°8'8"W), 1♀ (IBSP 172804); 1♂ 4♀, 25/VIII–03/IX/2009 (IBSP 172803, IBSP 174322); Cave N5S_0070 (6°6'7"S, 50°8'5"W), 2♀ (IBSP 172805, IBSP 172806); 4♀ 25/VIII–03/IX/2009 (IBSP 172657, IBSP 172667, IBSP 172668); Cave N5S_0071 (6°6'4"S, 50°8'8"W), 1♀ (IBSP 172808); 1♂, 25/VIII–03/IX/2009 (IBSP 172807); Cave N5S_0072 (6°6'4"S, 50°8'7"W), 1♀ (IBSP 172809); 1♀, 25/VIII–03/IX/2009 (IBSP 172644); Cave N5S_0073 (6°6'3"S, 50°8'6"W), 5♀ (IBSP 172812, IBSP 172813); 4♀, 25/VIII–03/IX/2009 (IBSP 172675, IBSP 172676); Cave N5S_0074 (6°6'3"S, 50°8'6"W), 1♂ (IBSP 172814); 2♀, 25/VIII–03/IX/2009 (IBSP 172669, IBSP 172670); Cave N5S_0075 (6°6'3"S, 50°8'2"W), 1♂ 2♀ (IBSP 172810, IBSP 172811); 2♂ 1♀, 25/VIII–03/IX/2009 (IBSP 172649–IBSP 172651); Cave N5S_0078 (6°6'9"S, 50°8'15"W), 4♀ (IBSP 172815, IBSP 172816, IBSP 174324); 1♀, 15–21/IX/2009 (IBSP 172686); Cave N5S_0079 (6°6'10"S, 50°8'14"W), 3♀ (IBSP 172817, IBSP 177562); 1♂ 6♀, 15–21/IX/2009 (IBSP 172680, IBSP 172681, IBSP 172682, IBSP 174320); Cave N5S_0081 (6°6'10"S, 50°8'14"W), 3♀ (IBSP 172818); Cave N5S_0082 (6°6'11"S, 50°8'14"W), 2♀ (IBSP 174506); Cave N5S_0083 (6°6'20"S, 50°8'13"W), 2♀ (IBSP 172819, IBSP 172820); 1♂ 2♀, 15–21/IX/2009 (IBSP 172687); Cave N5S_0085 (6°5'13"S, 50°7'36"W), 7♀ (IBSP 172821–IBSP 172825); 5♀, 25/VIII–03/IX/2009 (IBSP 172645–IBSP 172648), all collected on 14/III–04/IV/2010 by R. Andrade et al.; Cave N5S_0025 (6°5'13"S, 50°7'40"W), 1♀ (IBSP 172835); 2♀, 14–16/XII/2010 (IBSP 172826); Cave N5S_0028 (6°5'19"S, 50°7'33"W), 2♀ (IBSP 172836); 1♀ (IBSP 172827); Cave N5S_0030 (6°5'20"S, 50°7'12"W), 6♀ (IBSP 172838–IBSP 172841); 4♀ (IBSP 172829–IBSP 172831); Cave N5S_0032 (6°5'35"S, 50°7'10"W), 1♂ 6♀ (IBSP 172842–IBSP 172844); Cave N5S_0033 (6°6'14"S, 50°7'33"W), 4♀ (IBSP 172845, IBSP 172846); 1♀ (IBSP 172833); Cave N5S_0035 (6°6'21"S, 50°7'50"W), 1♀ (IBSP 172847); 1♀ (IBSP 172832); Cave N5PSE_01 (6°6'15"S, 50°9'6"W), 1♀ (IBSP 172851); Cave N5PSE_02 (6°6'14"S, 50°9'6"W), 2♀ (IBSP 172852); 1♀, 11–13/XII/2010 (IBSP 172848); Cave N5PSE_04 (6°6'21"S, 50°9'7"W), 1♀ (IBSP 177777); Cave N5PSE_05 (6°6'21"S, 50°9'8"W), 1♂ 7♀ (IBSP 172853–IBSP 172855); 2♂ 6♀ (IBSP 172849, IBSP 172850); Cave N5PSE_06 (6°6'4"S, 50°9'6"W), 1♂ (IBSP 172856); Cave N5PSL_01 (6°4'8"S, 50°8'41"W), 1♀ (IBSP 177778); Cave N5PSL_02 (6°3'45"S, 50°7'54"W), 1♀ (IBSP 172857); Cave N5PSL_03 (6°3'45"S, 50°7'54"W), 1♂ (IBSP 177779) all collected on 11–13/XII/2010 by I. Cizauskas et al.; Cave N4WS_0011 (6°4'9"S, 50°11'38"W), 3♂ 4♀ (IBSP 174772); Cave N4WS_0012 (6°4'0"S, 50°11'46"W), 2♂ (IBSP 174775); Cave N4WS_0014 (6°3'54"S, 50°11'21"W), 2♀ (IBSP 174784) all collected 20/X–01/XI/2006 by R. Andrade et al.; Cave N4WS_0008 (6°5'22"S, 50°11'41"W), 1♀ (IBSP 174800); 2♀, 10–19/V/2011 (IBSP 173497, IBSP 173498); 1♂ 3♀, 18/XI–01/XII/2010 (IBSP 173420–IBSP 173423); Cave N4WS_0015 (6°3'59"S, 50°11'22"W), 5♂ 6♀ (IBSP 174807, IBSP 174815); 1♂ 21♀, 20/IV–04/V/2010 (IBSP 173400–IBSP 173414); 1♂ 16♀, 20/X–01/XI/2006 (IBSP 174792), all collected by R. Andrade et al.; Cave N4WS_0050 (6°4'44"S, 50°11'36"W), 1♀ (IBSP 172769); 1♂ 1♀, 25/VIII–03/IX/2009 (IBSP 172652); Cave N4WS_0067 (6°4'23"S, 50°11'31"W), 1♀ (IBSP 177563); 1♀ 20/IV–04/V/2010 (IBSP 184745); Cave N4WS_0076 (6°4'29"S, 50°11'20"W), 1♂ (IBSP 174325) all collected 14/III–04/IV/2010 by F. P. Franco et al.; Cave N4WS_0013 (6°3'59"S, 50°11'23"W), 4♀ (IBSP 173398, IBSP 173399); 1♀ 20/X–01/XI/2006 (IBSP 174781); Cave N4WS_0017 (6°3'54"S, 50°11'44"W), 1♂ 2♀ (IBSP 173415, IBSP 173416); 1♂ 8♀ 20/X–01/XI/2006 (IBSP 174797), all collected by F. P. Franco et al.; Cave N4WS_0022 (6°3'48"S, 50°11'34"W), 2♂ 3♀ (IBSP 173478); Cave N4WS_0026 (6°3'56"S, 50°11'28"W), 2♀ (IBSP 173424); Cave N4WS_0027 (6°3'56"S, 50°11'29"W), 2♀ (IBSP 173445); Cave N4WS_0029 (6°3'49"S, 50°11'30"W), 1♀ (IBSP 173442); Cave N4WS_0042 (6°4'24"S, 50°11'41"W), 1♀ (IBSP 173449); Cave N4WS_0044 (6°4'23"S, 50°11'43"W), 1♀ (IBSP 173450); Cave N4WS_0046 (6°4'30"S, 50°11'40"W), 1♀ (IBSP 173451); Cave N4WS_0047 (6°4'32"S, 50°11'40"W), 2♀ (IBSP 173426, IBSP 173427); Cave N4WS_0054 (6°5'15"S, 50°11'42"W), 1♂ 1♀ (IBSP 173465); Cave N4WS_0056 (6°4'35"S, 50°11'29"W), 1♀ (IBSP 173428); Cave N4WS_0061 (6°4'37"S, 50°11'40"W), 1♂ 2♀ (IBSP 173479); Cave N4WS_0068 (6°4'24"S, 50°11'31"W), 1♀ (IBSP 173458); Cave N4WS_0074 (6°4'20"S, 50°11'23"W), 1♂ (IBSP 173460), all collected 18/XI–01/XII/2010 by F. P. Franco et al.; Cave N4WS_0001 (6°3'46"S, 50°11'32"W), 1♀ (IBSP 173480); 2♂ 2♀, (IBSP 173429, IBSP 173430, IBSP 173431); Cave N4WS_0002 (6°4'22"S, 50°11'40"W), 1♂ 3♀ (IBSP 173481, IBSP 173482); Cave N4WS_0003 (6°4'21"S, 50°11'41"W), 3♀ (IBSP 173483, IBSP 173484); 2♀ (IBSP 173417); Cave N4WS_0004 (6°4'21"S, 50°11'43"W), 10♀ (IBSP 173485–IBSP 173489, IBSP 177775, IBSP 177776); 8♀ (IBSP 173432–IBSP 173436, IBSP 177774); Cave N4WS_0005 (6°4'27"S, 50°11'40"W), 1♀ (IBSP 173490); 1♀ (IBSP 173437); Cave N4WS_0009 (6°5'22"S, 50°11'41"W), 1♂ 3♀ (IBSP 173499, IBSP 173500) 2♂ 9♀, (IBSP 173466–IBSP 173470); Cave N4WS_0010 (6°5'23"S, 50°11'42"W), 1♂ 8♀ (IBSP 173501–IBSP 173505); 7♀ (IBSP 173474–IBSP 173477); Cave N4WS_0020 (6°4'29"S, 50°11'0"W), 6♀ (IBSP 173507–IBSP 173509); 1♂ (IBSP 173473); Cave N4WS_0024 (6°3'49"S, 50°11'31"W), 1♂ 1♀ (IBSP 172834, IBSP 173511); Cave N4WS_0035 (6°5'7"S, 50°10'56"W), 2♀ (IBSP 173515, IBSP 173516); 1♂ 2♀, (IBSP 173443, IBSP 173444); Cave N4WS_0037 (6°6'45"S, 50°10'56"W), 1♂ 2♀ (IBSP 173517, IBSP 173518); 1♂ 1♀, (IBSP 173446, IBSP 173447); Cave N4WS_0041 (6°4'24"S, 50°11'42"W), 4♀ (IBSP 173519, IBSP 173520); 1♀ (IBSP 173448); Cave N4WS_0043 (6°4'23"S, 50°11'42"W), 3♀ (IBSP 173521); Cave N4WS_0058 (6°4'25"S, 50°11'41"W), 2♀ (IBSP 173529); Cave N4WS_0063 (6°4'23"S, 50°11'47"W), 3♂ 7♀ (IBSP 173530–IBSP 173532); 1♂ 6♀ (IBSP 173479, IBSP 173454–IBSP 173457); Cave N4WS_0064 (6°4'53"S, 50°11'45"W), 3♀ (IBSP 173533–IBSP 173535) 3♀ (IBSP 173471, IBSP 173472); Cave N4WS_0065 (6°5'55"S, 50°11'27"W), 3♀ (IBSP 173536); Cave N4WS_0069 (6°4'19"S, 50°11'40"W), 2♀ (IBSP 173537); 1♀ (IBSP 173459); Cave N4WS_0073 (6°4'26"S, 50°11'39"W), 1♂ 3♀ (IBSP 173538, IBSP 174378); 1♂ 2♀ (IBSP 173462, IBSP 173463); Cave N4WS_0075 (6°4'29"S, 50°11'23"W), 1♀ (IBSP 173541); 1♀ (IBSP 173464); Cave N4WS_0078 (6°4'22"S, 50°11'23"W), 1♀ (IBSP 173539); Cave N4WS_0079 (6°4'34"S, 50°11'28"W), 1♀ (IBSP 173540); Cave N4WS_0080 (6°4'34"S, 50°11'28"W), 1♀ (IBSP 173542); 1♀ (IBSP 173461), all collected 18/XI–01/XII/2010 by F. P. Franco et al.; Cave N4WS_0006 (6°4'36"S, 50°11'36"W), 3♀ (IBSP 173491, IBSP 173492); 3♀ (IBSP 173418, IBSP 173419); Cave N4WS_0007 (6°5'22"S, 50°11'41"W), 6♀ (IBSP 173493–IBSP 173496); 1♂ 3♀ (IBSP 173438–IBSP 173440); Cave N4WS_0018 (6°4'35"S, 50°11'38"W), 1♀ (IBSP 173506); 1♀ (IBSP 173441); Cave N4WS_0021 (6°3'59"S, 50°11'24"W), 1♀ (IBSP 173510); Cave N4WS_0031 (6°4'1"S, 50°11'26"W), 3♀ (IBSP 173512); Cave N4WS_0032 (6°4'5"S, 50°11'33"W), 2♀ (IBSP 173513, IBSP 173514) 1♀ (IBSP 173425); Cave N4WS_0049 (6°4'39"S, 50°11'39"W), 1♂ 1♀ (IBSP 173522, IBSP 173523); Cave N4WS_0055 (6°4'51"S, 50°11'46"W), 1♂ 5♀ (IBSP 173524–IBSP 173528) 1♂ 1♀ (IBSP 173452, IBSP 173453), all collected 18/XI–01/XII/2010 by F. P. Franco et al.; Cave N4WS_0040 (6°3'59"S, 50°11'37"W), 2♀ (IBSP 181381, IBSP 181382), Equipe Carste coll.; Cave N5SM1_0005 (6°6'42"S, 50°8'8"W), 1♂ 2♀ 01/IX/2010 (IBSP 176988); Cave N5SM1_0031 (6°6'20"S, 50°8'19"W), 2♀ 21/II/2011 (IBSP 177007); Cave N5SM1_0032 (6°6'19"S, 50°8'19"W), 2♀ 07–12/X/2008 (IBSP 176982, IBSP 177011); 1♂, 07/II/2011 (IBSP 184732), all collected by R. Zampaulo et al.; Cave N5SM1_0009 (6°6'27"S, 50°8'2"W), 6♀ (ISLA 14823); Cave N5SM2_0001 (6°8'32"S, 50°8'1"W), 1♂ 5♀ (ISLA 14691, ISLA 14718); Cave N5SM2_0002 (6°8'31"S, 50°8'3"W), 3♀ (ISLA 14654, ISLA 14759); Cave N5SM2_0003 (6°8'31"S, 50°8'6"W), 1♂ 3♀ (ISLA 14698, ISLA 14730); Cave N5SM2_0004 (6°8'28"S, 50°8'5"W), 5♀ (ISLA 14660, ISLA 14727); Cave N5SM2_0005 (6°8'27"S, 50°8'8"W), 8♀ (ISLA 14706, ISLA 14732); Cave N5SM2_0006 (6°8'28"S, 50°8'9"W), 6♀ (ISLA 14692); Cave N5SM2_0007 (6°8'27"S, 50°8'11"W), 1♂ 5♀ (ISLA 14657, ISLA 14724); Cave N5SM2_0008 (6°8'27"S, 50°8'9"W), 3♀ (ISLA 14758); Cave N5SM2_0009 (6°8'29"S, 50°8'14"W), 2♀ (ISLA 14734); Cave N5SM2_0010 (6°8'24"S, 50°8'17"W), 1♀ (ISLA 14668); Cave N5SM2_0011 (6°8'21"S, 50°8'17"W), 2♀ (ISLA 14714); Cave N5SM2_0012 (6°8'18"S, 50°8'18"W), 1♂ 2♀ (ISLA 14663); Cave N5SM2_0013 (6°8'16"S, 50°8'10"W), 1♂ 4♀ (ISLA 14666); Cave N5SM2_0014 (6°8'18"S, 50°8'0"W), 2♂ 1♀ (ISLA 14701); Cave N5SM2_0015 (6°8'17"S, 50°8'1"W), 2♀ (ISLA 14669, ISLA 14671); Cave N5SM2_0016 (6°8'17"S, 50°7'59"W), 3♀ (ISLA 14688); Cave N5SM2_0017 (6°8'19"S, 50°8'2"W), 2♀ (ISLA 14655, ISLA 14744); Cave N5SM2_0018 (6°8'18"S, 50°8'2"W), 2♀ (ISLA 14751); Cave N5SM2_0020 (6°7'59"S, 50°7'52"W), 1♀ (ISLA 14726); Cave N5SM2_0021 (6°7'58"S, 50°7'52"W), 7♀ (ISLA 14708, ISLA 14778); Cave N5SM2_0022 (6°8'8"S, 50°8'7"W), 3♀ (ISLA 14771); Cave N5SM2_0024 (6°8'8"S, 50°8'6"W), 1♂ 4♀ (ISLA 14680, ISLA 14763); Cave N5SM2_0025 (6°8'9"S, 50°8'6"W), 1♀ (ISLA 14746); Cave N5SM2_0026 (6°8'9"S, 50°8'6"W), 6♀ (ISLA 14681, ISLA 14749); Cave N5SM2_0027 (6°8'6"S, 50°8'12"W), 1♂ 9♀ (ISLA 14679, ISLA 14745); Cave N5SM2_0028 (6°8'4"S, 50°8'15"W), 1♀ (ISLA 14697); Cave N5SM2_0029 (6°8'5"S, 50°8'9"W), 1♂ (ISLA 14674); Cave N5SM2_0032 (6°8'3"S, 50°8'9"W), 1♀ (ISLA 14713); Cave N5SM2_0033 (6°8'3"S, 50°8'8"W), 1♂ (ISLA 14686); Cave N5SM2_0034 (6°8'2"S, 50°8'8"W), 2♀ (ISLA 14747); Cave N5SM2_0035 (6°8'2"S, 50°8'6"W), 1♂ 4♀ (ISLA 14650, ISLA 14752); Cave N5SM2_0036 (6°7'60"S, 50°8'4"W), 1♀ (ISLA 14757); Cave N5SM2_0037 (6°7'59"S, 50°8'5"W), 3♀ (ISLA 14651, ISLA 14753); Cave N5SM2_0038 (6°7'58"S, 50°8'5"W), 2♀ (ISLA 14725); Cave N5SM2_0039 (6°7'58"S, 50°8'6"W), 1♀ (ISLA 14768); Cave N5SM2_0040 (6°7'58"S, 50°8'12"W), 5♀ (ISLA 14659); Cave N5SM2_0041 (6°7'58"S, 50°8'12"W), 1♂ (ISLA 14777); Cave N5SM2_0042 (6°7'57"S, 50°8'11"W), 1♂ 4♀ (ISLA 14694); Cave N5SM2_0043 (6°7'56"S, 50°8'10"W), 1♂ 2♀ (ISLA 14682, ISLA 14779); Cave N5SM2_0044 (6°7'56"S, 50°8'6"W), 4♀ (ISLA 14723, ISLA 14770); Cave N5SM2_0045 (6°7'55"S, 50°8'6"W), 1♂ 7♀ (ISLA 14705, ISLA 14782); Cave N5SM2_0046 (6°7'54"S, 50°8'6"W), 8♀ (ISLA 14693, ISLA 14775); Cave N5SM2_0049 (6°7'52"S, 50°8'5"W), 1♂ 6♀ (ISLA 14658, ISLA 14739); Cave N5SM2_0050 (6°7'51"S, 50°8'6"W), 1♂ 1♀ (ISLA 14704, ISLA 14767); Cave N5SM2_0051 (6°7'51"S, 50°8'5"W), 1♂ 2♀ (ISLA 14675, ISLA 14743); Cave N5SM2_0052 (6°7'51"S, 50°8'5"W), 1♂ (ISLA 14750); Cave N5SM2_0053 (6°7'49"S, 50°8'5"W), 2♀ (ISLA 14703, ISLA 14719); Cave N5SM2_0054 (6°7'48"S, 50°8'4"W), 5♀ (ISLA 14667, ISLA 14738); Cave N5SM2_0056 (6°7'47"S, 50°8'5"W), 1♂ 5♀ (ISLA 14773); Cave N5SM2_0057 (6°7'47"S, 50°8'5"W), 1♂ 20♀ (ISLA 14687, ISLA 14760); Cave N5SM2_0058 (6°7'46"S, 50°8'5"W), 3♀ (ISLA 14656); Cave N5SM2_0059 (6°7'45"S, 50°8'5"W), 2♀ (ISLA 14672, ISLA 14699); Cave N5SM2_0060 (6°7'43"S, 50°8'9"W), 1♀ (ISLA 14676); Cave N5SM2_0061 (6°7'43"S, 50°8'6"W), 2♂ 4♀ (ISLA 14677, ISLA 14766); Cave N5SM2_0063 (6°7'42"S, 50°8'5"W), 2♀ (ISLA 14781); Cave N5SM2_0064 (6°7'43"S, 50°8'7"W), 5♀ (ISLA 14695, ISLA 14774); Cave N5SM2_0065 (6°7'41"S, 50°8'8"W), 1♀ (ISLA 14683); Cave N5SM2_0067 (6°7'39"S, 50°8'12"W), 1♂ 7♀ (ISLA 14664, ISLA 14772); Cave N5SM2_0068 (6°7'34"S, 50°8'15"W), 1♀ (ISLA 14685); Cave N5SM2_0069 (6°7'39"S, 50°7'55"W), 1♂ (ISLA 14649); Cave N5SM2_0070 (6°7'31"S, 50°7'55"W), 2♀ (ISLA 14769); Cave N5SM2_0071 (6°7'31"S, 50°7'55"W), 1♂ 1♀ (ISLA 14737); Cave N5SM2_0072 (6°7'31"S, 50°7'54"W), 1♂ (ISLA 14741); Cave N5SM2_0074 (6°7'32"S, 50°7'56"W), 2♀ (ISLA 14729); Cave N5SM2_0075 (6°7'32"S, 50°7'55"W), 1♂ 7♀ (ISLA 14670, ISLA 14712, ISLA 14755); Cave N5SM2_0076 (6°7'31"S, 50°7'54"W), 2♂ 6♀ (ISLA 14696, ISLA 14762); Cave N5SM2_0077 (6°7'28"S, 50°7'52"W), 2♀ (ISLA 14716); Cave N5SM2_0078 (6°7'23"S, 50°7'49"W), 3♂ 17♀ (ISLA 14673, ISLA 14756); Cave N5SM2_0079 (6°7'23"S, 50°7'50"W), 2♂ 1♀ (ISLA 14715); Cave N5SM2_0080 (6°7'20"S, 50°7'47"W), 1♂ 11♀ (ISLA 14689, ISLA 14711); Cave N5SM2_0081 (6°7'19"S, 50°7'44"W), 4♀ (ISLA 14662, ISLA 14740); Cave N5SM2_0082 (6°7'20"S, 50°7'43"W), 1♀ (ISLA 14731); Cave N5SM2_0084 (6°7'20"S, 50°7'41"W), 1♀ (ISLA 14710); Cave N5SM2_0086 (6°7'16"S, 50°7'47"W), 3♀ (ISLA 14653); Cave N5SM2_0087 (6°7'16"S, 50°7'43"W), 1♂ 3♀ (ISLA 14665); Cave N5SM2_0088 (6°7'15"S, 50°7'44"W), 5♂ 9♀ (ISLA 14690, ISLA 14733); Cave N5SM2_0089 (6°7'15"S, 50°7'44"W), 11♀ (ISLA 14684, ISLA 14754, ISLA 14761, ISLA 14765); Cave N5SM2_0090 (6°7'14"S, 50°7'45"W), 4♀ (ISLA 14700, ISLA 14721); Cave N5SM2_0091 (6°7'14"S, 50°7'46"W), 2♀ (ISLA 14717); Cave N5SM2_0092 (6°7'17"S, 50°7'55"W), 3♀ (ISLA 14722); Cave N5SM2_0093 (6°7'17"S, 50°7'56"W), 6♀ (ISLA 14678, ISLA 14735); Cave N5SM2_0094 (6°7'10"S, 50°7'54"W), 2♀ (ISLA 14702, ISLA 14776); Cave N5SM2_0095 (6°7'6"S, 50°7'54"W), 4♀ (ISLA 14764); Cave N5SM2_0096 (6°8'6"S, 50°8'12"W), 2♂ 5♀ (ISLA 14661, ISLA 14742); Cave N5SM2_0097 (6°7'42"S, 50°8'9"W), 1♀ (ISLA 14652); Cave N5SM2_0098 (6°8'28"S, 50°8'3"W), 2♂ 2♀ (ISLA 14736); Cave N5SM2_0100 (6°7'17"S, 50°7'55"W), 3♀ (ISLA 14707, ISLA 14728); Cave N5SM2_0101 (6°7'16"S, 50°7'54"W), 5♀ (ISLA 14780); Cave N5SM2_0102 (6°7'17"S, 50°7'52"W), 3♀ (ISLA 14720); Cave GEM-1747, 2♀ (ISLA 14709), all collected on 2010-2011 by Equipe Carste; Cave PPOR_0001 (6°3'53"S, 50°4'56"W), 5♀, 03–17/IV/2013 (IBSP 173903, IBSP 173904, IBSP 173905, IBSP 173906); 1♀, 26/IX–17/X/2012 (IBSP 173897); Cave PPOR_0002 (6°3'24"S, 50°4'49"W), 1♀, 26/IX–17/X/2012 (IBSP 173898); Cave PPOR_0003 (6°3'2"S, 50°5'57"W), 1♀, 26/IX–17/X/2012 (IBSP 173899) all collected by Equipe Carste; Cave N2_0005 (6°3'10"S, 50°14'32"W), 1♀ (IBSP 173357); Cave N2_0012 (6°3'10"S, 50°14'32"W), 1♀ (IBSP 173358); Cave N3_0008 (6°1'48"S, 50°12'4"W), 1♂ (IBSP 173704); Cave N3_0014 (6°1'59"S, 50°12'21"W), 1♂ 1♀ (IBSP 173711, IBSP 173712); Cave N3_0018 (6°1'44"S, 50°12'2"W), 3♀ (IBSP 173714, IBSP 173715); Cave N3_0025 (6°2'30"S, 50°13'6"W), 1♂ 1♀ (IBSP 173727, IBSP 173728); Cave N3_0058 (6°2'31"S, 50°13'24"W), 4♀ (IBSP 173731, IBSP 173757, IBSP 173758); Cave N3_0059 (6°2'30"S, 50°12'52"W), 1♀ (IBSP 173759); Cave N3_0060 (6°2'27"S, 50°13'8"W), 1♂ 2♀ (IBSP 173760, IBSP 173761) all collected on 26/IX–17/X/2012 by Equipe Carste coll.; Cave N3_0001 (6°1'47"S, 50°12'5"W), 2♂ (IBSP 173763, IBSP 173764); 2♀ (IBSP 173697); Cave N3_0002 (6°1'45"S, 50°12'4"W), 1♀ (IBSP 173698); 2♀ (IBSP 173765); Cave N3_0003 (6°1'45"S, 50°12'4"W), 5♀ (IBSP 173766–IBSP 173770); 1♂ 1♀ (IBSP 173699); Cave N3_0004 (6°1'46"S, 50°12'4"W), 1♀ (IBSP 173771); 3♀ (IBSP 173700–IBSP 173702); Cave N3_0005 (6°1'47"S, 50°12'5"W), 1♀ (IBSP 173772); Cave N3_0007 (6°1'48"S, 50°12'4"W), 2♀ (IBSP 173773); 1♀ (IBSP 173703); Cave N3_0009 (6°1'47"S, 50°12'4"W), 1♀ (IBSP 173774); Cave N3_0011 (6°1'49"S, 50°12'2"W), 3♀ (IBSP 173775, IBSP 173776); 4♀ (IBSP 173705–IBSP 173707); Cave N3_0013 (6°2'1"S, 50°12'5"W), 3♀ (IBSP 173777, IBSP 173778); 1♂ 2♀ (IBSP 173708–IBSP 173710); Cave N3_0016 (6°2'3"S, 50°12'42"W), 1♀ (IBSP 173780); Cave N3_0017 (6°2'2"S, 50°12'49"W), 3♀ (IBSP 173781–IBSP 173783); 1♀ (IBSP 173713); Cave N3_0019 (6°1'43"S, 50°12'2"W), 2♀ (IBSP 173784, IBSP 173785); 1♀ (IBSP 173716); Cave N3_0020 (6°2'6"S, 50°12'34"W), 1♂ 11♀ (IBSP 173786–IBSP 173792); 12♀ (IBSP 173717–IBSP 173726); Cave N3_0026 (6°2'33"S, 50°13'7"W), 4♀ (IBSP 173798–IBSP 173801); 2♀ (IBSP 173729, IBSP 173730); Cave N3_0027 (6°2'41"S, 50°13'14"W), 1♂ (IBSP 173802); Cave N3_0033 (6°2'43"S, 50°13'14"W), 2♀ (IBSP 173803, IBSP 173804); Cave N3_0036 (6°2'47"S, 50°13'15"W), 3♀ (IBSP 173805, IBSP 173806); 1♀ (IBSP 173732); Cave N3_0037 (6°2'46"S, 50°13'15"W), 2♀ (IBSP 173807, IBSP 173808); 1♂ 11♀ (IBSP 173733–IBSP 173739); Cave N3_0039 (6°2'25"S, 50°13'23"W), 1♀ (IBSP 173809); 2♀ (IBSP 173740, IBSP 173741); Cave N3_0041 (6°2'24"S, 50°13'16"W), 1♂ 4♀ (IBSP 173810–IBSP 173813); 1♂ 7♀ (IBSP 173742–IBSP 173748); Cave N3_0042 (6°2'23"S, 50°13'16"W), 10♀ (IBSP 173814–IBSP 173818); 8♀ (IBSP 173749–IBSP 173753); Cave N3_0043 (6°2'14"S, 50°13'6"W), 3♀ (IBSP 173819–IBSP 173821); 1♀ (IBSP 173754); Cave N3_0045 (6°2'1"S, 50°12'22"W), 1♀ (IBSP 173822); Cave N3_0057 (6°2'33"S, 50°13'23"W), 2♂ 2♀ (IBSP 173824, IBSP 178965); Cave N3_0055 (6°2'30"S, 50°13'8"W), 1♂ (IBSP 173823); 1♂ 1♀ (IBSP 173755, IBSP 173756), all collected on 26/IX–17/X/2012 by Equipe Carste; Cave N2_0001 (6°3'17"S, 50°14'25"W), 2♀ (IBSP 173373); 2♀ (IBSP 173352, IBSP 173353); Cave N2_0003 (6°3'17"S, 50°14'41"W), 7♀ (IBSP 173374–IBSP 173379); 3♀ (IBSP 173354, IBSP 173355); Cave N2_0004 (6°3'10"S, 50°14'35"W), 3♀ (IBSP 173380, IBSP 173381); 1♀ (IBSP 173356); Cave N2_0007 (6°3'15"S, 50°14'23"W), 3♀ (IBSP 173382–IBSP 173384); Cave N2_0008 (6°3'15"S, 50°14'23"W), 1♀ (IBSP 173385); Cave N2_0009 (6°3'15"S, 50°14'23"W), 1♀ (IBSP 173386); Cave N2_0013 (6°3'10"S, 50°14'34"W), 3♀ (IBSP 173387, IBSP 173359); Cave N2_0014 (6°3'2"S, 50°14'57"W), 1♀ (IBSP 173388); Cave N2_0015 (6°3'3"S, 50°14'56"W), 1♀ (IBSP 173389); Cave N2_0016 (6°3'10"S, 50°14'35"W), 1♀ (IBSP 173390); 1♂ 3♀ (IBSP 173360–IBSP 173363); Cave N2_0017 (6°3'7"S, 50°14'40"W), 1♂ 1♀ (IBSP 173391); Cave N2_0022 (6°3'1"S, 50°15'3"W), 2♀ (IBSP 173392); 2♂ 3♀ (IBSP 173364–IBSP 173367); Cave N2_0026 (6°2'5"S, 50°12'34"W), 7♀ (IBSP 173393–IBSP 173397); 1♂ 7♀ (IBSP 173368–IBSP 173372); Cave N3_0015 (6°2'7"S, 50°12'29"W), 1♀ (IBSP 173779); Cave N3_0032 (6°2'39"S, 50°13'12"W), 1♀ (IBSP 173827); Cave N3_0049 (6°2'26"S, 50°13'38"W), 1♀ (IBSP 173836); Cave N3_0050 (6°2'25"S, 50°13'40"W), 1♀ (IBSP 173842); Cave N3_0071 (6°2'38"S, 50°13'52"W), 1♂ 1♀ (IBSP 173864, IBSP 173865); Cave N3_0073 (6°2'38"S, 50°13'47"W), 2♀ (IBSP 173868, IBSP 173869); Cave N3_0072 (6°2'38"S, 50°13'51"W), 2♀ (IBSP 173866, IBSP 173867); Cave N3_0078 (6°2'37"S, 50°13'44"W), 2♀ (IBSP 173872); Cave N3_0067 (6°2'31"S, 50°13'36"W), 1♂ 3♀ (IBSP 173855–IBSP 173857); 1♀ (IBSP 173762); Cave N3_0068 (6°2'30"S, 50°13'35"W), 2♂ 7♀ (IBSP 173858–IBSP 173862); Cave N3_0064 (6°2'29"S, 50°13'37"W), 1♀ (IBSP 173853); Cave N3_0065 (6°2'28"S, 50°13'37"W), 1♂ (IBSP 173854); Cave N6_0003 (6°8'9"S, 50°10'5"W), 1♂ 1♀ (IBSP 173900, IBSP 173901); Cave N6_0004 (6°7'31"S, 50°10'12"W), 1♀ (IBSP 173902), all collected on 26/IX–17/X/2012 by Equipe Carste; Cave N3_0024 (6°2'28"S, 50°13'7"W), 2♀ (IBSP 173873, IBSP 173874) 6♀ 05–17/III/2013 (IBSP 173793–IBSP 173797); Cave N3_0038 (6°2'22"S, 50°13'29"W), 1♂ 1♀ (IBSP 173875, IBSP 173876); 1♂ 03–17/IV/2013 (IBSP 173828); Cave N3_0070 (6°2'40"S, 50°13'50"W), 1♂ (IBSP 173889); 3♀ 03–17/IV/2013 (IBSP 173863); Cave N3_0051 (6°2'39"S, 50°13'33"W), 1♀ (IBSP 173882); Cave N3_0056 (6°2'38"S, 50°13'32"W), 1♂ (IBSP 173887); 2♂ 4♀, 03–17/IV/2013 (IBSP 173845–IBSP 173848); Cave N3_0074 (6°2'37"S, 50°13'51"W), 3♀ (IBSP 173890–IBSP 173892); 2♀, 05–17/III/2013 (IBSP 173825, IBSP 173826); Cave N3_0063 (6°2'33"S, 50°13'36"W), 1♂ 2♀ (IBSP 173888); 6♀, 03–17/IV/2013 (IBSP 173849, IBSP 173850–IBSP 173852); Cave N3_0076 (6°2'30"S, 50°13'37"W), 1♂ 3♀ (IBSP 173893–IBSP 173896); 3♀, 03–17/IV/2013 (IBSP 173870, IBSP 173871); Cave N3_0047 (6°2'28"S, 50°13'41"W), 5♀ (IBSP 173877–IBSP 173881); 2♂ 1♀, 03–17/IV/2013 (IBSP 173829–IBSP 173835); Cave N3_0052 (6°2'27"S, 50°13'44"W), 1♂ 4♀ (IBSP 173883–IBSP 173885); 6♀, 03–17/IV/2013 (IBSP 173837–IBSP 173841); Cave N3_0054 (6°2'27"S, 50°13'43"W), 1♀ (IBSP 173886); 1♂ 3♀, 03–17/IV/2013 (IBSP 173843, IBSP 173844); Cave N5W_0001 (6°4'47"S, 50°8'0"W), 5♀ (IBSP 172858–IBSP 172860); Cave N5W_0002 (6°4'48"S, 50°8'1"W), 2♀ (IBSP 172861); Cave N5W_0003 (6°4'52"S, 50°8'4"W), 1♂ 7♀ (IBSP 172862–IBSP 172868); Cave N5W_0007 (6°4'52"S, 50°8'4"W), 1♀ (IBSP 172869); Cave N5W_0008 (6°4'54"S, 50°8'4"W), 1♂ 4♀ (IBSP 172870–IBSP 172874) all collected on 02–23/VIII/2013 by Equipe Carste; Cave N6_0005 (6°7'22"S, 50°10'28"W), 1♀ (IBSP 181310); Cave N6_0006 (6°8'8"S, 50°10'3"W), 1♀ (IBSP 181311); Cave N8_0028 (6°10'33"S, 50°9'29"W), 2♀ (IBSP 181329, IBSP 181330); Cave N8_0021 (6°10'7"S, 50°9'28"W), 1♀ (IBSP 181328); Cave N8_0012 (6°10'7"S, 50°9'29"W), 1♀ (IBSP 181327); 1♀ 24/II–13/III/2015 (IBSP 181547); Cave N8_0002 (6°10'5"S, 50°9'35"W), 1♀ (IBSP 181312); Cave N8_0031 (6°9'54"S, 50°9'30"W), 6♀ (IBSP 181331–IBSP 181334), all collected on 16/VII–06/VIII/2014 by Equipe Carste; Cave N8_0017 (6°10'7"S, 50°9'27"W), 2♀ (IBSP 181386, IBSP 181387); Cave N8_0004 (6°10'7"S, 50°9'29"W), 1♀ (IBSP 181472), collected by Equipe Carste; Cave N8_0019 (6°10'11"S, 50°9'27"W), 1♀ (IBSP 181544); Cave N8_0010 (6°10'10"S, 50°9'35"W), 2♀ (IBSP 181545, IBSP 181546); 3♀, 16/VII–06/VIII/2014 (IBSP 181324–IBSP 181326); Cave N8_0018 (6°10'8"S, 50°9'28"W), 1♂ 1♀ (IBSP 181551, IBSP 181552); Cave N8_0011 (6°10'8"S, 50°9'29"W), 1♀ (IBSP 181547); Cave N8_0009 (6°10'8"S, 50°9'36"W), 3♀ (IBSP 181541, IBSP 181542, IBSP 181543); 2♂ 7♀, 16/VII–06/VIII/2014 (IBSP 181316–IBSP 181323); Cave N8_0023 (6°10'8"S, 50°9'30"W), 2♀ (IBSP 181555); Cave N8_0008 (6°10'7"S, 50°9'34"W), 1 (IBSP 181532–IBSP 181540); 2♀, 16/VII–06/VIII/2014 (IBSP 181314, IBSP 181315); Cave N8_0014 (6°10'7"S, 50°9'31"W), 1♀ (IBSP 181550); Cave N8_0022 (6°10'6"S, 50°9'30"W), 2♀ (IBSP 181553, IBSP 181554); Cave N8_0007 (6°10'6"S, 50°9'36"W), 1♂ 3♀ (IBSP 181528–IBSP 181531); Cave N8_0003 (6°10'6"S, 50°9'32"W), 1♀ (IBSP 181525); 1♂ 1♀, 04/IX–06/X/2014 (IBSP 181383, IBSP 181384); Cave N8_0005 (6°10'6"S, 50°9'32"W), 1♀ (IBSP 181526); Cave N8_0013 (6°10'6"S, 50°9'31"W), 2♀ (IBSP 181548, IBSP 181549); 1♀, 04/IX–06/X/2014 (IBSP 181385); Cave N8_0006 (6°10'5"S, 50°9'38"W), 1♂ (IBSP 181527); 1♀, 16/VII–06/VIII/2014 (IBSP 181313); Cave N8_0036 (6°10'1"S, 50°9'13"W), 1♂ 1♀ (IBSP 181559, IBSP 181560); Cave N8_0033 (6°10'0"S, 50°9'16"W), 2♀ (IBSP 181556, IBSP 181557); Cave N8_0035 (6°9'59"S, 50°9'14"W), 3♀ (IBSP 181558); 1♀, 16/VII–06/VIII/2014 (IBSP 181335), all collected by Equipe Carste; Cave N1_0002 (6°2'26"S, 50°16'13"W), 2♀ (IBSP 174650); Cave N1_0004 (6°2'25"S, 50°16'14"W), 3♂ 7♀ (IBSP 174653, IBSP 174654); 4♀, 16/VII–06/VIII/2014 (IBSP 181276–IBSP 181279); Cave N1_0008 (6°2'21"S, 50°16'15"W), 2♀ (IBSP 174658, IBSP 174659); 3♀, 11/VI–02/VII/2014 (IBSP 181240–IBSP 181242); 1♀, 24/II–13/III/2015 (IBSP 181445); Cave N1_0014 (6°2'4"S, 50°16'21"W), 3♀ (IBSP 174661); 1♀, 03–17/XII/2014 (IBSP 181388); 2♀, 11/VI–02/VII/2014 (IBSP 181243); Cave N1_0015 (6°2'3"S, 50°16'17"W), 5♀ (IBSP 174666); 3♀, 11/VI–02/VII/2014 (IBSP 181244, IBSP 181245); 3♀, 24/II–13/III/2015 (IBSP 181446–IBSP 181448); Cave N1_0018 (6°2'3"S, 50°16'19"W), 3♀ (IBSP 174670); 2♀, 07–28/I/2015 (IBSP 181420, IBSP 181421); 1♂ 1♀, 11/VI–02/VII/2014 (IBSP 181246, IBSP 181247); Cave N1_0022 (6°1'58"S, 50°16'20"W), 5♀ (IBSP 174736); 1♀, 07–28/I/2015 (IBSP 181429); 1♀, 16/VII–06/VIII/2014 (IBSP 181283); Cave N1_0025 (6°1'54"S, 50°16'21"W), 1♂ 5♀ (IBSP 174684, IBSP 174686); 2♂ 1♀, 02–29/IV/2015 (IBSP 181481, IBSP 181482); 1♂ 2♀, 04/IX–06/X/2014 (IBSP 181337, IBSP 181338); Cave N1_0037 (6°1'51"S, 50°16'29"W), 2♀ (IBSP 174687); Cave N1_0039 (6°1'48"S, 50°16'16"W), 3♀ (IBSP 174689); 1♀, 02–29/IV/2015 (IBSP 181484); 1♀, 04/IX–06/X/2014 (IBSP 181344); Cave N1_0072 (6°1'15"S, 50°17'19"W), 8♀ (IBSP 174693, IBSP 174696); Cave N1_0075 (6°1'16"S, 50°16'50"W), 2♂ 5♀ (IBSP 174701, IBSP 174703); Cave N1_0116 (6°0'41"S, 50°18'52"W), 3♀ (IBSP 174710); Cave N1_0119 (6°1'17"S, 50°18'8"W), 5♀ (IBSP 174713); 1♀, 02–29/IV/2015 (IBSP 181503); 2♀, 16/VII–06/VIII/2014 (IBSP 181294, IBSP 181295); Cave N1_0143 (6°1'38"S, 50°17'29"W), 1 (IBSP 174717, IBSP 174719); Cave N1_0170 (6°1'25"S, 50°17'60"W), 1♂ 6♀ (IBSP 174722, IBSP 174723); 1♀, 03–17/XII/2014 (IBSP 181402); Cave N1_0173 (6°1'29"S, 50°17'57"W), 3♀ (IBSP 174725); 1♀, 07–28/I/2015 (IBSP 181440); 1♀, 16/VII–06/VIII/2014 (IBSP 181307); Cave N1_0176 (6°1'30"S, 50°18'4"W), 2♀ (IBSP 174729); Cave N1_0180 (6°2'35"S, 50°16'26"W), 1♀ (IBSP 174732); Cave N1_0212 (6°1'49"S, 50°18'3"W), 1♀ (IBSP 174735), all collected by R. Andrade et al.; Cave N1_0074 (6°1'18"S, 50°16'51"W), 1♀ (IBSP 181257); Cave N1_0084 (6°1'8"S, 50°17'2"W), 1♀ (IBSP 181258); Cave N1_0104 (6°0'34"S, 50°18'3"W), 1♀ (IBSP 181259); Cave N1_0114 (6°0'49"S, 50°18'24"W), 1♂ 2♀ (IBSP 181260, IBSP 181261); Cave N1_0149 (6°2'32"S, 50°16'30"W), 2♀ (IBSP 181264, IBSP 181265); 1♀, 24/II–13/III/2015 (IBSP 181462); Cave N1_0156 (6°2'42"S, 50°16'23"W), 1♀ (IBSP 181267); Cave N1_0237 (6°1'17"S, 50°16'27"W), 1♀ (IBSP 181271); Cave N1_0228 (6°1'11"S, 50°17'3"W), 1♀ (IBSP 181270), all collected on 11/VI–02/VII/2014 by Equipe Carste; Cave N1_0186 (6°2'38"S, 50°16'35"W), 1♀ (IBSP 181309); 1♀, 24/II–13/III/2015 (IBSP 181464), all collected by Equipe Carste; Cave N1_0016 (6°1'11"S, 50°16'42"W), 1♂ (IBSP 181336); Cave N1_0031 (6°1'47"S, 50°16'21"W), 1♂ (IBSP 181339); Cave N1_0033 (6°1'52"S, 50°16'30"W), 1♀ (IBSP 181340); Cave N1_0035 (6°1'51"S, 50°16'30"W), 1♀ (IBSP 181341); Cave N1_0043 (6°1'51"S, 50°16'34"W), 1♀ (IBSP 181346); Cave N1_0044 (6°1'14"S, 50°16'43"W), 1♀ (IBSP 181347); 1♀ (IBSP 181451); Cave N1_0045 (6°1'45"S, 50°16'33"W), 1♀ (IBSP 181348); 1♀ (IBSP 181452); Cave N1_0049 (6°1'42"S, 50°16'33"W), 1♀ (IBSP 181349); Cave N1_0052 (6°1'38"S, 50°16'34"W), 1♀ (IBSP 181350); 1♀ (IBSP 181454); Cave N1_0067 (6°1'44"S, 50°17'22"W), 2♀ (IBSP 181352, IBSP 181353); Cave N1_0088 (6°1'3"S, 50°17'6"W), 1♀ (IBSP 181354); Cave N1_0138 (6°1'34"S, 50°16'32"W), 1♀ (IBSP 181357); Cave N1_0160 (6°2'36"S, 50°16'25"W), 1♀ (IBSP 181358); Cave N1_0178 (6°2'24"S, 50°17'29"W), 2♀ (IBSP 181360, IBSP 181361); 1♀ (IBSP 181463); Cave N1_0198 (6°2'48"S, 50°17'27"W), 1♀ (IBSP 181362); 2♀ (IBSP 181467); Cave N1_0199 (6°2'54"S, 50°17'29"W), 1♀ (IBSP 181363); 1♀ (IBSP 181468); Cave N1_0203 (6°2'43"S, 50°16'43"W), 3♀ (IBSP 181364, IBSP 181365); Cave N1_0204 (6°2'43"S, 50°16'37"W), 1♀ (IBSP 181366); 1♀ (IBSP 181469); Cave N1_0205 (6°2'43"S, 50°16'37"W), 1♀ (IBSP 181367); 1♀ (IBSP 181470); Cave N1_0214 (6°2'27"S, 50°17'51"W), 1♀ (IBSP 181368); Cave N1_0230 (6°2'25"S, 50°17'32"W), 1♂ 1♀ (IBSP 181373, IBSP 181374); 1♀ (IBSP 181471); Cave N1_0218 (6°2'9"S, 50°17'23"W), 1♀ (IBSP 181369); Cave N1_0221 (6°1'50"S, 50°18'3"W), 1♀ (IBSP 181370); Cave N1_0240 (6°1'20"S, 50°16'28"W), 6♀ (IBSP 181376, IBSP 181377, IBSP 181378, IBSP 181379), all collected on 24/II–13/III/2015 by Equipe Carste coll.; Cave N1_0017 (6°2'3"S, 50°16'23"W), 2♀ (IBSP 181389, IBSP 181390); Cave N1_0154 (6°2'43"S, 50°16'22"W), 1♀ (IBSP 181391); 2♀, 11/VI–02/VII/2014 (IBSP 181266); Cave N1_0158 (6°2'40"S, 50°16'24"W), 1♀ (IBSP 181392); 2♀, 11/VI–02/VII/2014 (IBSP 181268, IBSP 181269); Cave N1_0168 (6°1'18"S, 50°18'6"W), 2♂ 6♀ (IBSP 181393–IBSP 181399); Cave N1_0169 (6°1'25"S, 50°18'1"W), 1♂ 1♀ (IBSP 181400, IBSP 181401); Cave N1_0171 (6°1'19"S, 50°18'5"W), 1♂ 6♀ (IBSP 181403–IBSP 181409); 1♂, 4♀, 16/VII–06/VIII/2014 (IBSP 181303–IBSP 181306); Cave N1_0239 (6°1'20"S, 50°16'28"W), 1♀ (IBSP 181416); Cave N1_0236 (6°1'17"S, 50°16'26"W), 2♀ (IBSP 181412, IBSP 181413); Cave N1_0238 (6°1'16"S, 50°16'26"W), 2♀ (IBSP 181414, IBSP 181415); 2♀, 11/VI–02/VII/2014 (IBSP 181272, IBSP 181273); Cave N1_0232 (6°1'16"S, 50°16'23"W), 1♂ (IBSP 181411); Cave N1_0247 (6°1'15"S, 50°16'24"W), 2♀ (IBSP 181417, IBSP 181418); 1♀ (IBSP 181275); Cave N1_0231 (6°1'15"S, 50°16'23"W), 1♀ (IBSP 181410), all collected by Equipe Carste coll.; Cave N1_0013 (6°2'4"S, 50°16'17"W), 1♀ (IBSP 181419); Cave N1_0019 (6°2'2"S, 50°16'19"W), 2♀ (IBSP 181422, IBSP 181423); Cave N1_0020 (6°1'59"S, 50°16'19"W), 2♂ 2♀ (IBSP 181424, IBSP 181425, IBSP 181426, IBSP 181427); 1♀ ,16/VII–06/VIII/2014 (IBSP 181281); Cave N1_0021 (6°1'59"S, 50°16'20"W), 1♀ (IBSP 181428); 1♀, 16/VII–06/VIII/2014 (IBSP 181282); Cave N1_0054 (6°1'15"S, 50°17'12"W), 1♀ (IBSP 181430); Cave N1_0055 (6°1'13"S, 50°16'47"W), 1♀ (IBSP 181431); Cave N1_0056 (6°1'13"S, 50°16'45"W), 1♀ (IBSP 181432); Cave N1_0060 (6°1'14"S, 50°16'43"W), (IBSP 181433); 1♀, 11/VI–02/VII/2014 (IBSP 181256); Cave N1_0096 (6°1'10"S, 50°17'1"W), 1♀ (IBSP 181434); Cave N1_0118 (6°0'42"S, 50°18'54"W), 1♀ (IBSP 181435); Cave N1_0125 (6°0'16"S, 50°17'17"W), 2♂ (IBSP 181436, IBSP 181437); 1♂, 11/VI–02/VII/2014 (IBSP 181262); Cave N1_0141 (6°2'36"S, 50°16'34"W), 1♀ (IBSP 181438); 1♂ 7♀, 16/VII–06/VIII/2014 (IBSP 181296–IBSP 181300); Cave N1_0145 (6°2'35"S, 50°16'30"W), 1♀ (IBSP 181439); 1♂ 1♀, 16/VII–06/VIII/2014 (IBSP 181301, IBSP 181302); Cave N1_0174 (6°1'29"S, 50°17'55"W), 3♀ (IBSP 181441, IBSP 181442); 1♀ 16/VII–06/VIII/2014 (IBSP 181308); Cave N1_0210 (6°1'30"S, 50°17'52"W), 1♀ (IBSP 181443) all collected by Equipe Carste coll.; Cave N1_0005 (6°2'24"S, 50°16'12"W), 1♀ (IBSP 181444); Cave N1_0029 (6°1'49"S, 50°16'21"W), 2♀ (IBSP 181449); Cave N1_0032 (6°1'52"S, 50°16'30"W), 1♀ (IBSP 181450); Cave N1_0046 (6°1'44"S, 50°16'33"W), 1♂ (IBSP 181453); Cave N1_0076 (6°1'16"S, 50°16'50"W), 2♀ (IBSP 181456, IBSP 181457); Cave N1_0077 (6°1'16"S, 50°16'54"W), 1♀ (IBSP 181455); Cave N1_0085 (6°1'8"S, 50°17'2"W), 1♀ (IBSP 181458); Cave N1_0098 (6°1'11"S, 50°17'7"W), 1♀ (IBSP 181459); Cave N1_0101 (6°1'9"S, 50°16'48"W), 2♀ (IBSP 181460, IBSP 181461); Cave N1_0187 (6°2'39"S, 50°16'35"W), 3♀ (IBSP 181465); Cave N1_0188 (6°2'43"S, 50°16'34"W), 1♀ (IBSP 181466), all collected on 24/II–13/III/2015 by Equipe Carste coll.; Cave N1_0010 (6°1'12"S, 50°16'44"W), 1♀ (IBSP 181474); 1♀, 16/VII–06/VIII/2014 (IBSP 181280); Cave N1_0024 (6°1'54"S, 50°16'22"W), 1♂ 5♀ (IBSP 181475–IBSP 181480); 1♀, 11/VI–02/VII/2014 (IBSP 181248–IBSP 181255); Cave N1_0038 (6°1'50"S, 50°16'19"W), 1♀ (IBSP 181483); 2♀, 04/IX–06/X/2014 (IBSP 181342, IBSP 181343); Cave N1_0041 (6°1'46"S, 50°16'10"W), 1♀ (IBSP 181485); 1♀, 04/IX–06/X/2014 (IBSP 181345); Cave N1_0051 (6°1'50"S, 50°16'14"W), 2♀ (IBSP 181486); Cave N1_0059 (6°1'12"S, 50°16'46"W), 1♂ 1♀ (IBSP 181487); Cave N1_0062 (6°1'11"S, 50°16'46"W), 2♀ (IBSP 181488, IBSP 181489) 1♀ 04/IX–06/X/2014 (IBSP 181351); Cave N1_0073 (6°1'10"S, 50°16'47"W), 9♀ (IBSP 181490–IBSP 181497); 1♂ 9♀, 16/VII–06/VIII/2014 (IBSP 181284–IBSP 181293); Cave N1_0091 (5°59'57"S, 50°17'58"W), 2♀ (IBSP 181498, IBSP 181499); Cave N1_0092 (6°0'43"S, 50°18'16"W), 1♀ (IBSP 181500); Cave N1_0105 (6°0'37"S, 50°18'10"W), 2♀ (IBSP 181501, IBSP 181502); Cave N1_0129 (6°0'37"S, 50°17'30"W), 2♀ (IBSP 181504, IBSP 181505); 1♀, 11/VI–02/VII/2014 (IBSP 181263); Cave N1_0134 (6°1'5"S, 50°17'30"W), 1♀ (IBSP 181506); Cave N1_0137 (6°1'33"S, 50°16'31"W), 1♂ 2♀ (IBSP 181507, IBSP 181508); 2♀, 04/IX–06/X/2014 (IBSP 181355, IBSP 181356); Cave N1_0147 (6°2'34"S, 50°16'28"W), 2♀ (IBSP 181509, IBSP 181510); Cave N1_0148 (6°2'33"S, 50°16'27"W), 1♀ (IBSP 181511); Cave N1_0165 (6°1'10"S, 50°18'28"W), 1♀ (IBSP 181512); 2♀, 04/IX–06/X/2014 (IBSP 181359); Cave N1_0181 (6°2'24"S, 50°17'29"W), 1♀ (IBSP 181513); Cave N1_0213 (6°2'4"S, 50°17'55"W), 1♀ (IBSP 181514); Cave N1_0225 (6°2'18"S, 50°16'4"W), 1♀ (IBSP 181516); 1♀, 04/IX–06/X/2014 (IBSP 181372); Cave N1_0242 (6°1'52"S, 50°16'12"W), 2♀ (IBSP 181519, IBSP 181520); 1♀, 04/IX–06/X/2014 (IBSP 181380); Cave N1_0233 (6°1'16"S, 50°16'24"W), 3♀ (IBSP 181517, IBSP 181518); 1♀, 04/IX–06/X/2014 (IBSP 181375); Cave N1_0222 (6°0'36"S, 50°17'28"W), 1♀ (IBSP 181515); 1♀, 04/IX–06/X/2014 (IBSP 181371); Cave N1_0245 (6°0'32"S, 50°18'8"W), 1♀ (IBSP 181522); 1♂, 11/VI–02/VII/2014 (IBSP 181274); Cave N1_0246 (6°0'31"S, 50°18'7"W), 2♀ (IBSP 181523, IBSP 181524); Cave N1_0244 (6°0'26"S, 50°18'17"W), 1♀ (IBSP 181521), all collected by Equipe Carste coll.; Canaã dos Carajás, Cave CRIS_002 (6°27'35"S, 49°41'3"W), 1♀ (IBSP 174552); Cave CRIS_007 (6°27'9"S, 49°40'43"W), 3♀(IBSP 174561); Cave CRIS_010 (6°26'56"S, 49°41'9"W), 2♀ (IBSP 174571); Cave CRIS_014 (6°26'51"S, 49°40'59"W), 1♀ (IBSP 174583); Cave CRIS_015 (6°26'51"S, 49°40'58"W), 1♂ (IBSP 174585); Cave CRIS_018 (6°26'4"S, 49°41'13"W), 1♂ (IBSP 174593); Cave CRIS_024 (6°27'33"S, 49°42'44"W), 1♀ (IBSP 174604); Cave CRIS_025 (6°27'35"S, 49°42'40"W), 5♀ (IBSP 174606); Cave CRIS_026 (6°27'35"S, 49°42'40"W), 1♀ (IBSP 174616); Cave CRIS_028 (6°27'33"S, 49°42'35"W), 1♀ (IBSP 174623); Cave CRIS-033 (6°27'38"S, 49°42'26"W), 3♀ (IBSP 174636); Cave CRIS_035 (6°27'34"S, 49°42'18"W), 1♂ (IBSP 174639); Cave CRIS_036 (6°27'34"S, 49°42'18"W), 2♂ 4♀ (IBSP 174641); Cave CRIS_038 (6°27'33"S, 49°42'14"W), 2♀ (IBSP 174646), all collected on 29/VII–06/VIII/2008 by R. Andrade et al.; Cave CRIS_016 (6°26'21"S, 49°40'57"W), 2♂ 6♀ 22/II/2011 (IBSP 175226, IBSP 175227, IBSP 175951, IBSP 175952); Cave CRIS_017 (6°26'22"S, 49°40'56"W), 4♀ 22/II/2011 (IBSP 175228–IBSP 175230); 4♀, 23/II/2011 (IBSP 175953–IBSP 175955) collected by R. Andrade & V. Felice; Cave SB_0021 (6°18'36"S, 50°0'11"W), 1♂ (IBSP 173682); 1♀, 29/VIII–27/IX/2012 (IBSP 173601); Cave SB_0022 (6°18'26"S, 50°0'7"W), 1♀ (IBSP 173683); 1♀, 10–31/I/2013 (IBSP 173638) collected by C. A. R. Souza & J. Mascarenhas, Cave SB_0159 (6°20'44"S, 49°50'29"W), 1♀ (IBSP 173592); Cave SB_0147 (6°18'5"S, 49°49'40"W), 1♀ (IBSP 173595); Cave SB_0158 (6°20'44"S, 49°50'29"W), 2♀ (IBSP 173593); Cave SB_0002 (6°19'1"S, 49°59'36"W), 1♀ (IBSP 173603); Cave SB_0005 (6°17'9"S, 49°55'39"W), 1♀ (IBSP 173604); Cave SB_0042 (6°18'56"S, 49°53'41"W), 1♀ (IBSP 173607); Cave SB_0087 (6°17'36"S, 49°56'32"W), 1♂ 1♀ (IBSP 174379); Cave SB_0094 (6°18'2"S, 49°57'29"W), 1♂ (IBSP 173606) all collected on 29/VIII–27/IX/2012 by C. A. R. Souza et al.; Cave SB_0034 (6°19'0"S, 49°53'45"W), 1♀ (IBSP 173545); 1♀, 29/VIII–27/IX/2012 (IBSP 173596); Cave SB_0035 (6°18'59"S, 49°53'48"W), 1♀ (IBSP 173546); Cave SB_0036 (6°19'1"S, 49°53'42"W), 1♀ (IBSP 173547); Cave SB_0038 (6°18'52"S, 49°53'1"W), 1♀ (IBSP 173548); Cave SB_0041 (6°18'56"S, 49°52'55"W), 1♂ 6♀ (IBSP 173549–IBSP 173552); 3♀, 29/VIII–27/IX/2012 (IBSP 173618); Cave SB_044 (6°18'60"S, 49°53'42"W), 1♀ (IBSP 173553); 2♀, 20/IX–01/X/2011 (IBSP 173590, IBSP 173591); Cave SB_0046 (6°18'59"S, 49°53'42"W), 2♀ (IBSP 173554); 1♂ 2♀, 29/VIII–27/IX/2012 (IBSP 173612–IBSP 173614); Cave SB_0060 (6°18'45"S, 49°52'59"W), 1♀ (IBSP 173555); Cave SB_0062 (6°18'45"S, 49°52'59"W), 1♀ (IBSP 173556); Cave SB_0063 (6°16'38"S, 49°55'5"W), 1♀ (IBSP 173557); 1♂, 29/VIII–27/IX/2012 (IBSP 173610); Cave SB_0067 (6°16'35"S, 49°55'5"W), 1♀ (IBSP 173558); Cave SB_0071 (6°16'35"S, 49°55'7"W), 4♀ (IBSP 173578, IBSP 173579); 2♀, 29/VIII–27/IX/2012 (IBSP 173631, IBSP 173632); Cave SB_0074 (6°18'41"S, 49°52'6"W), 1♀ (IBSP 173559); 1♂, 29/VIII–27/IX/2012 (IBSP 173630); Cave SB_0085 (6°18'40"S, 49°54'43"W), 1♀ (IBSP 173560); 1♀, 29/VIII–27/IX/2012 (IBSP 173615); Cave SB_0086 (6°18'40"S, 49°54'43"W), 1♀ (IBSP 173562); Cave SB_0107 (6°18'53"S, 49°56'17"W), 2♀ (IBSP 173561) all collected on 17/I–02/II/2012 by C. A. R. Souza et al.; Cave SB_0007 (6°18'44"S, 50°0'0"W), 1♀ (IBSP 173568); Cave SB_0008 (6°18'47"S, 50°0'3"W), 1♀ (IBSP 173569); Cave SB_0009 (6°18'47"S, 50°0'3"W), 1♀ (IBSP 173577); Cave SB_0040 (6°18'59"S, 49°52'52"W), 2♀ (IBSP 173574, IBSP 173575); 2♀ (IBSP 173616, IBSP 173617); Cave SB_0047 (6°18'48"S, 49°52'51"W), 1♂ (IBSP 173566); Cave SB_0048 (6°18'51"S, 49°52'51"W), 1♂ (IBSP 173567); 1♀ (IBSP 173609); Cave SB_0050 (6°18'52"S, 49°52'51"W), 2♀ (IBSP 173571); Cave SB_0054 (6°18'35"S, 49°53'8"W), 1♂ 1♀ (IBSP 173580, IBSP 173581); Cave SB_0055 (6°16'36"S, 49°54'53"W), 1♀ (IBSP 183456); 2♀ (IBSP 173619, IBSP 173620); Cave SB_0056 (6°16'37"S, 49°54'53"W), 1♀ (IBSP 174326); Cave SB_0065 (6°18'6"S, 49°53'11"W), 1♀ (IBSP 173589); Cave SB_0066 (6°18'5"S, 49°53'10"W), 2♀ (IBSP 173587, IBSP 173588); Cave SB_0069 (6°16'36"S, 49°55'5"W), 6♀ (IBSP 173583–IBSP 173585); Cave SB_0070 (6°16'35"S, 49°55'6"W ), 1♀ (IBSP 173563); Cave SB_0072 (6°16'53"S, 49°55'40"W), 3♀ (IBSP 173572, IBSP 173573, IBSP 173586); 1♂ 2♀ (IBSP 173598–IBSP 173600); Cave SB_0073 (6°16'55"S, 49°55'41"W), 1♀ (IBSP 173582); 1♀ (IBSP 173636); Cave SB_0075 (6°17'47"S, 49°53'59"W), 2♀ (IBSP 173564); Cave SB_0082 (6°18'6"S, 49°55'27"W), 1♀ (IBSP 173576); 1♀ (IBSP 173635); Cave SB_0083 (6°18'5"S, 49°55'26"W), 1♂ (IBSP 173565); 1♀ (IBSP 173611); Cave SB_0100 (6°16'3"S, 49°57'4"W), 1♀ (IBSP 173570); 1♀ (IBSP 173597) all collected on 29/VIII–27/IX/2012 by C. A. R. Souza et al.; Cave SB_0161 (6°20'10"S, 49°51'0"W), 3♀ (IBSP 173629); 1♀, 28/I–05/II2013 (IBSP 173677); Cave SB_0138 (6°19'13"S, 49°49'54"W), 2♀ (IBSP 173634); Cave SB_0023 (6°19'24"S, 49°59'3"W), 1♀ (IBSP 173621); all collected on 18–26/X/2012 by C. A. R. Souza et al.; Cave SB_0152 (6°20'54"S, 49°50'30"W), 1♀ (IBSP 173657); Cave SB_0155 (6°20'46"S, 49°50'29"W), 2♀ (IBSP 173652, IBSP 173653); 1♀, 29/VIII–27/IX/2012 (IBSP 173594); Cave SB_0156 (6°20'45"S, 49°50'29"W), 1♀ (IBSP 173651); Cave SB_0135 (6°19'25"S, 49°49'58"W), 1♀ (IBSP 173656); 1♀, 18–26/X/2012 (IBSP 173633); Cave SB_0137 (6°19'13"S, 49°49'54"W), 3♀ (IBSP 173658, IBSP 173659, IBSP 173660); 2♀, 18–26/X/2012 (IBSP 173622, IBSP 173623); Cave SB_0139 (6°19'13"S, 49°49'53"W), 4♀ (IBSP 173670–IBSP 173672); 2♀, 29/VIII–27/IX/2012 (IBSP 173624); Cave SB_0001 (6°18'47"S, 49°59'47"W), 2♀ (IBSP 173679); 1♀, 29/VIII–27/IX/2012 (IBSP 173605); Cave SB_0006 (6°17'9"S, 49°55'37"W), 1♀ (IBSP 173675); Cave SB_0010 (6°18'42"S, 50°0'8"W), 1♀ (IBSP 173665); Cave SB_0011 (6°19'0"S, 49°59'37"W), 1♂ 1♀ (IBSP 173666, IBSP 173681); Cave SB_0013 (6°19'36"S, 49°58'26"W), 1♀ (IBSP 173667); 1♂, 28/I–05/II2013 (IBSP 173678); Cave SB_0017 (6°18'47"S, 49°59'56"W), 1♀ (IBSP 173668); 6♀, 28/I–05/II2013 (IBSP 173661–IBSP 173664); 1♀, 29/VIII–27/IX/2012 (IBSP 173602); Cave SB_0018 (6°18'45"S, 49°59'59"W), 2♀ (IBSP 173637); 1♀, 28/I–05/II2013 (IBSP 173640); Cave SB_0026 (6°18'49"S, 49°53'35"W), 1♀ (IBSP 173676) 1♀, 17/I–02/II/2012 (IBSP 173543); 5♀ 29/VIII–27/IX/2012 (IBSP 173625–IBSP 173628); Cave SB_0030 (6°18'51"S, 49°53'34"W), 1♀ (IBSP 173639); 1♀, 17/I–02/II/2012 (IBSP 173544); Cave SB_0080 (6°18'23"S, 49°56'46"W), 1♀ (IBSP 173642); Cave SB_0090 (6°18'5"S, 49°57'28"W), 1♂ 3♀ (IBSP 173647–IBSP 173650); Cave SB_0091 (6°18'5"S, 49°57'51"W), 1♀ (IBSP 173644); 1♀, 29/VIII–27/IX/2012 (IBSP 173608); Cave SB_0093 (6°18'43"S, 49°57'23"W), 1♀ (IBSP 173654); Cave SB_0096 (6°18'20"S, 49°56'47"W), 1♀ (IBSP 173643); Cave SB_0097 (6°18'18"S, 49°56'49"W), 1♀ (IBSP 173645); Cave SB_0106 (6°15'50"S, 49°58'38"W), 1♀ (IBSP 173646); Cave SB_0115 (6°21'14"S, 49°58'39"W), 1♀ (IBSP 173641); Cave SB_0117 (6°21'13"S, 49°58'43"W), 1♀ (IBSP 173655) all collected on 10–31/I/2013 by C. A. R. Souza & J. Mascarenhas et al.; Cave SB_0160 (6°20'12"S, 49°51'4"W), 1♀ (IBSP 173673); Cave SB_0134 (6°19'59"S, 49°50'55"W), 1♀ (IBSP 173680); Cave SB_0133 (6°18'54"S, 49°50'14"W), 1♀ (IBSP 173674); Cave SB_0039 (6°18'49"S, 49°52'56"W), 2♀ (IBSP 173669); all collected on 28/I–05/II2013 by C. A. R. Souza et al.; Cave SB_0227 (6°21'33"S, 49°59'35"W), 2♀ (IBSP 185237); Cave SB_0224 (6°21'32"S, 49°59'35"W), 1♀ (IBSP 185236); 1♀, 12–22/X/2013 (IBSP 181588); Cave SB_0222 (6°21'30"S, 49°59'36"W), 1♂ 1♀ (IBSP 185235); Cave SB_0187 (6°20'43"S, 49°58'22"W), 1♀ (IBSP 185223); Cave SB_0217 (6°20'43"S, 49°58'30"W), 1♀ (IBSP 185234); 1♀, 12–22/X/2013 (IBSP 181586); Cave SB_0186 (6°20'41"S, 49°58'18"W), 2♀ (IBSP 174381, IBSP 185222); Cave SB_0212 (6°20'25"S, 49°57'39"W), 1♂ 2♀ (IBSP 185231); 8♀ (IBSP 181578–IBSP 181582); Cave SB_0210 (6°20'22"S, 49°57'36"W), 1♀ (IBSP 185229); 2♀ (IBSP 181576, IBSP 181577); Cave SB_0208 (6°20'21"S, 49°57'36"W), 1♀ (IBSP 185227); Cave SB_0209 (6°20'21"S, 49°57'36"W), 1♀ (IBSP 185228); Cave SB_0193 (6°20'8"S, 49°57'28"W), 2♀ (IBSP 185225, IBSP 185226); 3♀ (IBSP 181574); Cave SB_0192 (6°20'3"S, 49°57'29"W), 2♀ (IBSP 185224); Cave SB_0183 (6°19'4"S, 49°57'55"W), 1♂ (IBSP 185221); Cave SB_0164 (6°19'3"S, 49°58'45"W), 3♀ (IBSP 185206, IBSP 185207); Cave SB_0169 (6°19'3"S, 49°58'44"W), 3♀ (IBSP 185211, IBSP 185212); 1♂ 2♀ (IBSP 181569, IBSP 181570); Cave SB_0168 (6°19'2"S, 49°58'45"W), 1♀ (IBSP 185210); 1♂ 4♀ (IBSP 181565–IBSP 181568); Cave SB_0167 (6°19'1"S, 49°58'45"W), 4♀ (IBSP 185208, IBSP 185209); 4♀ (IBSP 181563, IBSP 181564); Cave SB_0173 (6°18'52"S, 49°52'47"W), 1♀ (IBSP 185213); 1♀ (IBSP 181572); Cave SB_0175 (6°18'52"S, 49°52'49"W), 3♀ (IBSP 185215, IBSP 185216); Cave SB_0176 (6°18'51"S, 49°52'50"W), 5♀ (IBSP 185217, IBSP 185218); 1♀ (IBSP 181573); Cave SB_0174 (6°18'51"S, 49°52'48"W), 1♀ (IBSP 185214); Cave SB_0178 (6°18'49"S, 49°52'50"W), 1♀ (IBSP 185219); Cave SB_0179 (6°18'45"S, 49°52'50"W), 3♀ (IBSP 185220); Cave SB_0163 (6°18'44"S, 49°58'43"W), 1♀ (IBSP 185205); 1♂ (IBSP 181561); Cave SB_0216 (6°18'14"S, 49°58'33"W), 1♀ (IBSP 185233); Cave SB_0214 (6°18'14"S, 49°58'33"W), 1♂ 1♀ (IBSP 185232) all collected on 10–20/IX/2013 by C. A. R. Souza et al.; Cave SB_0112 (6°21'12"S, 49°58'37"W), 2♀ (IBSP 185238); Cave SB_0114 (6°21'12"S, 49°58'39"W), 1♀ (IBSP 185239); Cave SB_0125 (6°21'17"S, 49°59'11"W), 1♀ (IBSP 185240); Cave SB_0127 (6°21'16"S, 49°59'12"W), 1♀ (IBSP 185241) all collected on 20–26/VI/2013 by C. A. R. Souza et al.; Cave SB_0200 (6°20'43"S, 49°57'50"W), 1♀ (IBSP 181575); 1♂, 13–23/II/2014 (IBSP 181597); Cave SB_0166 (6°18'60"S, 49°58'45"W), 1♂ (IBSP 181562) 2♀ 13–23/II/2014 (IBSP 181593); Cave SB_0172 (6°18'50"S, 49°52'46"W), 1♀ (IBSP 181571) all collected on 10–20/IX/2013 by C. A. R. Souza et al.; Cave SB_0199 (6°20'43"S, 49°56'57"W), 1♀ (IBSP 181584); Cave SB_0218 (6°20'43"S, 49°58'31"W), 1♀ (IBSP 181587); Cave SB_0241 (6°20'41"S, 49°54'14"W), 1♀ (IBSP 181591); 2♀ (IBSP 181609, IBSP 181610); Cave SB_0239 (6°20'40"S, 49°54'13"W), 1♂ 1♀ (IBSP 181589, IBSP 181590); 1♂ 3♀ (IBSP 181604–IBSP 181607); Cave SB_0197 (6°20'37"S, 49°57'10"W), 1♀ (IBSP 181583); Cave SB_0243 (6°20'2"S, 49°54'20"W), 1♀ (IBSP 181592); Cave SB_0206 (6°18'26"S, 49°57'23"W), 1♀ (IBSP 181585); 2♀ (IBSP 181599); Cave SB_0205 (6°19'39"S, 49°59'3"W), 3♀ (IBSP 181611, IBSP 181612) all collected on 13–23/II/2014 by C. A. R. Souza et al.; Cave SB_0240 (6°20'40"S, 49°54'13"W), 2♀ (IBSP 181608); Cave SB_0238 (6°20'36"S, 49°54'16"W), 1♀ (IBSP 185243); Cave SB_0230 (6°20'36"S, 49°59'44"W), 1♀ (IBSP 174380); Cave SB_0235 (6°20'33"S, 49°54'18"W), 1♀ (IBSP 181603); Cave SB_0194 (6°20'31"S, 49°57'13"W), 2♂ 1♀ (IBSP 174323, IBSP 181595, IBSP 181596); Cave SB_0233 (6°20'29"S, 49°54'19"W), 3♀ (IBSP 181602, IBSP 185242); Cave SB_0213 (6°20'27"S, 49°57'40"W), 1♀ (IBSP 181601); Cave SB_0211 (6°20'25"S, 49°57'37"W), 1♀ (IBSP 181600); Cave SB_0188 (6°20'25"S, 49°57'55"W), 1♀ (IBSP 181594); Cave SB_0201 (6°20'25"S, 49°57'59"W), 1♀ (IBSP 181598) all collected on 13–23/II/2014 by C. A. R. Souza et al.; Cave CAV_02 (6°29'40"S, 51°9'46"W), 2♀ (IBSP 173908); 1♀, 20–29/VI/2012 (IBSP 173929); Cave CAV-03 (6°29'39"S, 51°9'48"W), 1♀ (IBSP 173911); Cave CAV_04 (6°29'39"S, 51°9'47"W), 6♀ (IBSP 173918, IBSP 174382); 6♀, 20–29/VI/2012 (IBSP 173930); Cave CAV_05 (6°29'58"S, 51°9'44"W), 2♀ (IBSP 173921); Cave CAV_07 (6°30'2"S, 51°9'41"W), 4♀, 08–15/III/2012 (IBSP 173913, IBSP 173923); 3♀, 20–29/VI/2012 (IBSP 173933); Cave CAV_09 (6°30'2"S, 51°9'41"W), 1♂ 5♀ (IBSP 173909, IBSP 173910, IBSP 173912); 1♀, 20–29/VI/2012 (IBSP 173928); Cave CAV_10 (6°30'8"S, 51°9'40"W), 1♀, 08–15/III/2012 (IBSP 173919); Cave CAV_13 (6°29'53"S, 51°9'44"W), 2♂ (IBSP 173907); 2♀, 20–29/VI/2012 (IBSP 173925); Cave CAV_14 (6°30'2"S, 51°9'41"W), 1♀ (IBSP 173922); 1♀, 20–29/VI/2012 (IBSP 173934); Cave CAV_15 (6°29'50"S, 51°9'32"W), 1♀(IBSP 173914); Cave CAV_16 (6°29'56"S, 51°9'43"W), 1♂ 4♀ (IBSP 173916); 1♂ 7♀, 20–29/VI/2012 (IBSP 173927); Cave CAV_17 (6°29'50"S, 51°9'32"W), 1♀ 08–15/III/2012 (IBSP 173915); 2♀, 20–29/VI/2012 (IBSP 173932); Cave CAV_18 (6°29'50"S, 51°9'31"W), 3♀ (IBSP 173920); 3♀, 20–29/VI/2012 (IBSP 173926); Cave CAV_20 (6°30'1"S, 51°9'41"W), 1♂ (IBSP 173917); 2♀, 20–29/VI/2012 (IBSP 173935); Cave CAV_21 (6°29'60"S, 51°9'41"W), 1♀ (IBSP 173924) all collected on 08–15/III/2012 by Equipe Carste coll.; Cave CAV_08 (6°29'52"S, 51°9'43"W), 1♀ 20–29/VI/2012 (IBSP 173931); collected by Equipe Carste; Cave S11A_0003 (6°21'1"S, 50°27'4"W), 1♀ (IBSP 174402); Cave S11A_0007 (6°21'7"S, 50°26'37"W), 3♀ (IBSP 174409); Cave S11A_0012 (6°19'54"S, 50°27'6"W), 2♀ (IBSP 174412); Cave S11A_0026 (6°18'28"S, 50°26'57"W), 2♂ 4♀(IBSP 174419, IBSP 174422); Cave S11B_0013 (6°21'17"S, 50°24'42"W), 1♀ (IBSP 174438); Cave S11B_0023 (6°20'45"S, 50°24'36"W), 2♀ (IBSP 174444); Cave S11B_0024 (6°20'45"S, 50°24'35"W), 1♀(IBSP 174447), all collected on 23/VIII–02/IX/2007 by R. Andrade et al.; Cave S11D_0048 (6°24'40"S, 50°18'56"W), 2♀ (IBSP 172483, IBSP 172484); Cave S11D_0005 (6°24'4"S, 50°21'1"W), 1♀ (IBSP 172520); Cave S11D_0100 (6°23'46"S, 50°20'28"W), 2♀ (IBSP 172466, IBSP 172467); Cave S11D_0101 (6°23'24"S, 50°21'50"W), 1♂ 2♀ (IBSP 172459, IBSP 172460, IBSP 172461); Cave S11D_0015 (6°23'47"S, 50°21'26"W), 1♀ (IBSP 172486); Cave S11D_0018 (6°24'14"S, 50°22'28"W), 1♀ (IBSP 172475); Cave S11D_0019 (6°24'17"S, 50°22'16"W), 1♀ (IBSP 172487); Cave S11D_0034 (6°24'42"S, 50°20'37"W), 1♂ 2♀ (IBSP 172510, IBSP 172511); Cave S11D_0035 (6°24'41"S, 50°20'36"W), 3♀ (IBSP 172514, IBSP 172515, IBSP 172516); Cave S11D_0036 (6°24'41"S, 50°20'36"W), 2♀ (IBSP 172505); Cave S11D_0038 (6°23'51"S, 50°20'27"W), 4♀ (IBSP 172500); Cave S11D_0040 (6°24'40"S, 50°19'31"W), 1♂ 2♀ (IBSP 172512, IBSP 172513); 3♀, 23/VIII–02/IX/2007 (IBSP 174477); Cave S11D_0044 (6°25'3"S, 50°18'56"W), 1♀ (IBSP 172485); Cave S11D_0046 (6°24'55"S, 50°19'0"W), 2♀ (IBSP 172506, IBSP 172507); Cave S11D_0051 (6°24'26"S, 50°19'16"W), 1♀ (IBSP 172482); Cave S11D_0059 (6°24'28"S, 50°18'47"W), 1♀ (IBSP 172481); Cave S11D_0062 (6°23'33"S, 50°18'49"W), 1♀ (IBSP 172494); Cave S11D_0065 (6°23'27"S, 50°18'50"W), 2♀ (IBSP 172491); Cave S11D_0068 (6°23'35"S, 50°19'9"W), 2♀ (IBSP 172497, IBSP 172498); Cave S11D_0070 (6°23'35"S, 50°19'10"W), 1♀ (IBSP 172499); Cave S11D_0073 (6°23'34"S, 50°19'8"W), 1♂ 5♀ (IBSP 172451, IBSP 173684); Cave S11D_0076 (6°23'34"S, 50°19'1"W), 2♀ (IBSP 172452, IBSP 172453); Cave S11D_0078 (6°23'34"S, 50°18'59"W), 1♂ 2♀ (IBSP 172457, IBSP 173685); 6♀, 23/VIII–02/IX/2007 (IBSP 174492, IBSP 174493); Cave S11D_0081 (6°23'35"S, 50°18'54"W), 3♀ (IBSP 172462–IBSP 172464); Cave S11D_0084 (6°23'50"S, 50°19'26"W), 3♀ (IBSP 172473, IBSP 172474); Cave S11D_0087 (6°23'47"S, 50°19'24"W), 1♀ (IBSP 172458); Cave S11D_0091 (6°23'45"S, 50°19'20"W), 1♀ (IBSP 172472); Cave S11D_0094 (6°23'42"S, 50°19'19"W), 2♀ (IBSP 172454, IBSP 172455); Cave S11D_0099 (6°23'46"S, 50°20'28"W), 1♂ 1♀ (IBSP 172469), all collected on 13–30/I/2010 by R. Andrade et al.; Cave S11D_0003 (6°24'3"S, 50°21'1"W), 1♂ (IBSP 173686); Cave S11D_0020 (6°24'45"S, 50°21'38"W), 1♀ (IBSP 172526); Cave S11D_0021 (6°24'45"S, 50°21'36"W), 1♂ 1♀ (IBSP 173687); Cave S11D_0023 (6°24'48"S, 50°21'34"W), 2♀ (IBSP 172529); Cave S11D_0025 (6°24'50"S, 50°21'45"W), 1♀ (IBSP 172530); Cave S11D_0028 (6°24'41"S, 50°21'7"W), 1♀ (IBSP 172539); Cave S11D_0029 (6°24'42"S, 50°20'45"W), 4♀ (IBSP 172540, IBSP 172541); Cave S11D_0037 (6°24'48"S, 50°21'32"W), 3♀ (IBSP 172542, IBSP 172543); Cave S11D_0027 (6°24'44"S, 50°21'11"W), 1♂ 6♀ (IBSP 172536, IBSP 172537, IBSP 172538), all collected on 19–22/II/2010 by R. Andrade et al.; Cave S11_0006 (6°26'21"S, 50°17'34"W), 2♀ (IBSP 172559); Cave S11_0019 (6°26'37"S, 50°17'31"W), 1♀ (IBSP 172560); Cave S11_0020 (6°26'39"S, 50°17'31"W), 1♀ (IBSP 172561); Cave S11_0029 (6°26'33"S, 50°17'34"W), 1♀ (IBSP 172564), all collected on 19–22/III/2010 by R. Andrade et al.; Cave S11_0001 (6°24'28"S, 50°14'54"W), 3♀ (IBSP 172544, IBSP 172545); Cave S11_0012 (6°25'12"S, 50°15'1"W), 1♀ (IBSP 172551); Cave S11_0013 (6°25'13"S, 50°15'2"W), 2♀ (IBSP 172550); Cave S11_0014 (6°25'13"S, 50°15'2"W), 1♀ (IBSP 172552); Cave S11_0018 (6°26'10"S, 50°17'45"W), 1♂ 1♀ (IBSP 172555, IBSP 172556); Cave S11_0023 (6°25'25"S, 50°18'0"W), 2♀ (IBSP 172546, IBSP 172547); Cave S11_0024 (6°25'23"S, 50°18'5"W), 1♀ (IBSP 172548), all collected on 24/II–04/III/2010 by R. Andrade & I. Cizauskas; Cave CAV_0002 (6°24'46"S, 50°20'9"W), 1♀ (IBSP 172568); Cave CAV_0003 (6°24'43"S, 50°20'6"W), 1♂ 3♀ (IBSP 172569–IBSP 172571); Cave CAV_0007 (6°24'41"S, 50°19'59"W), 1♀ (IBSP 172575); Cave CAV_0023 (6°24'56"S, 50°21'41"W), 3♀ (IBSP 172583); Cave CAV_0024 (6°24'22"S, 50°21'58"W), 1♂ (IBSP 172584); Cave CAV_0025 (6°24'56"S, 50°21'41"W), 1♀ (IBSP 172585); Cave CAV_0022 (6°24'24"S, 50°22'11"W), 1♀ (IBSP 172582); Cave CAV_0032 (6°25'37"S, 50°19'27"W), 1♀ (IBSP 172586); Cave CAV_0040 (6°24'57"S, 50°21'50"W), 1♂ 3♀ (IBSP 172590); Cave CAV_0041 (6°24'57"S, 50°21'52"W), 3♀ (IBSP 172591, IBSP 172592); Cave CAV-08B (6°24'43"S, 50°19'52"W), 2♀ (IBSP 172576), all collected on 22–31/V/2010 by R. Andrade et al.; Cave S11D_0047 (6°24'40"S, 50°18'56"W), 1♀ (IBSP 172521) 1♀ 13–30/I/2010 (IBSP 172614); Cave S11D_0055 (6°24'24"S, 50°19'14"W), 1♀ (IBSP 172488–IBSP 172490); 1♂ 3♀, 13–30/I/2010 (IBSP 172615); 1♀, 23/VIII–02/IX/2007 (IBSP 174483, IBSP 174486); Cave S11D_0060 (6°23'35"S, 50°18'43"W), 1♀ (IBSP 172492, IBSP 172493); 3♀, 13–30/I/2010 (IBSP 172616); Cave S11D_0069 (6°23'36"S, 50°19'9"W), 2♀ (IBSP 172617); 2♀, 13–30/I/2010 (IBSP 172495, IBSP 172496); Cave S11D_0071 (6°23'35"S, 50°19'10"W), 1♀ (IBSP 172593); 1♀, 13–30/I/2010 (IBSP 172450); Cave S11D_0077 (6°23'34"S, 50°19'0"W), 1♀ (IBSP 172456); 3♀, 13–30/I/2010 (IBSP 172594); Cave S11D_0080 (6°23'35"S, 50°18'58"W), 1♀ (IBSP 174376); 2♀, 13–30/I/2010 (IBSP 172465); Cave S11D_0088 (6°23'46"S, 50°19'24"W), 3♀ (IBSP 172595, IBSP 172596); 1♀, 13–30/I/2010 (IBSP 172468); 1♀, 23/VIII–02/IX/2007 (IBSP 174496); Cave S11D_0089 (6°23'46"S, 50°19'22"W), 1♀ (IBSP 172597); 2♀, 13–30/I/2010 (IBSP 172470, IBSP 172471); Cave S11D_0096 (6°23'39"S, 50°19'28"W), 6♀ (IBSP 172598–IBSP 172601); 8♀, 13–30/I/2010 (IBSP 172476–IBSP 172480); 7♀, 23/VIII–02/IX/2007 (IBSP 174497), all collected on 01–14/VII/2010 by R. Andrade et al.; Cave S11_0007 (6°27'22"S, 50°14'31"W), 1♀ (IBSP 172557, IBSP 172558); 3♀, 24/II–04/III/2010 (IBSP 172631); Cave S11_0008 (6°25'14"S, 50°17'12"W), 1♀ (IBSP 172549); 2♀, 24/II–04/III/2010 (IBSP 172632); Cave S11_0015 (6°25'12"S, 50°15'2"W), 1♀ (IBSP 172553, IBSP 172554); 4♀, 24/II–04/III/2010 (IBSP 172633); Cave S11_0021 (6°26'41"S, 50°17'30"W), 1♀ (IBSP 172562, IBSP 172563); 3♀ 19–22/III/2010 (IBSP 172634); Cave S11_0025 (6°25'19"S, 50°18'20"W), 1♀ (IBSP 172635); Cave S11D_0022 (6°24'47"S, 50°21'35"W), 3♀ (IBSP 172531, IBSP 172532, IBSP 172533, IBSP 172534); 5♀, 19–22/II/2010 (IBSP 172602); Cave S11D_0024 (6°24'48"S, 50°21'17"W), 2♀ (IBSP 172527, IBSP 172528); 3♀, 19–22/II/2010 (IBSP 172603 IBSP 172604); Cave S11D_0026 (6°24'50"S, 50°21'19"W), 1♀ (IBSP 172535, IBSP 172536, IBSP 172537, IBSP 172538); 2♂ 6♀, 19–22/II/2010 (IBSP 172605); Cave S11D_0033 (6°24'41"S, 50°20'38"W), 1♂ 4♀ (IBSP 172501–IBSP 172504); 6♀ (IBSP 172606, IBSP 172607, IBSP 172608); 2♀, 23/VIII–02/IX/2007 (IBSP 174468, IBSP 174470); Cave S11D_0039 (6°23'48"S, 50°20'28"W), 2♀ (IBSP 172522–IBSP 172525); 4♀ (IBSP 173688); Cave S11D_0041 (6°23'33"S, 50°19'10"W), 2♀ (IBSP 172609); Cave S11D_0043 (6°24'49"S, 50°19'19"W), 1♂ 2♀ (IBSP 172517, IBSP 172518, IBSP 172519); 5♀, (IBSP 172610–IBSP 172612) 4♀ 23/VIII–02/IX/2007 (IBSP 174479); Cave S11D_0045 (6°24'57"S, 50°19'0"W), 3♀ (IBSP 172508, IBSP 172509); 5♀ (IBSP 172613) all collected on 13–30/I/2010 by R. Andrade et al.; Cave CAV_0001 (6°24'43"S, 50°20'7"W), 4♀ (IBSP 172565, IBSP 172566, IBSP 172567) 1♂ 5♀, 22–31/V/2010 (IBSP 172618–IBSP 172620); Cave CAV_0005 (6°24'42"S, 50°20'3"W), 1♀ (IBSP 172621); 2♀, 22–31/V/2010 (IBSP 172572, IBSP 172573); Cave CAV_0006 (6°24'41"S, 50°19'59"W), 1♀ (IBSP 172622); 1♀, 22–31/V/2010 (IBSP 172574); Cave CAV_0010 (6°24'40"S, 50°19'42"W), 1♀ (IBSP 172625); 1♂, 22–31/V/2010 (IBSP 172579); Cave CAV_0012 (6°24'40"S, 50°19'40"W), 1♀ (IBSP 172626); 1♂ 2♀, 22–31/V/2010 (IBSP 172580, IBSP 172581); Cave CAV_0028 (6°24'32"S, 50°22'9"W), 1♂ 1♀ (IBSP 174321); Cave CAV_0034 (6°24'10"S, 50°22'58"W), 1♀ (IBSP 172628); Cave CAV_0036 (6°24'33"S, 50°22'10"W), 1♀ (IBSP 172630); Cave CAV_0009 (6°25'21"S, 50°19'33"W), 2♀ (IBSP 172623, IBSP 172624); 4♀, 22–31/V/2010 (IBSP 172577, IBSP 172578); Cave CAV_0018 (6°24'25"S, 50°22'10"W), 2♀ (IBSP 174377); Cave CAV_0019 (6°24'22"S, 50°22'9"W), 1♀ (IBSP 172627); Cave CAV_0035 (6°24'24"S, 50°23'8"W), 2♂ (IBSP 172629); 2♂ 4♀, 22–31/V/2010 (IBSP 72587–IBSP 172589), all collected by R. Andrade et al.; Cave S11D_0116 (6°25'19"S, 50°19'0"W), 1♀ (IBSP 173696); 3♀, 30/VII–02/IX/2011 (IBSP 173690, IBSP 173691); Cave S11D_0112 (6°24'47"S, 50°21'16"W), 1♀ (IBSP 173695); 1♀, 30/VII–02/IX/2011 (IBSP 173689); Cave S11D_0104 (6°23'51"S, 50°22'0"W), 2♀ (IBSP 173693); 5♀, 30/VII–02/IX/2011 (IBSP 173692); Cave S11D_0111 (6°23'50"S, 50°20'29"W), 1♀ (IBSP 173694) all collected by R. Andrade et al.; Curionópolis, Cave SL_0074 (5°57'58"S, 49°37'56"W), 1♀ (IBSP 174771); Cave SL_0075 (5°57'56"S, 49°37'57"W), 4♀ (IBSP 174749); Cave SL_0089 (5°57'33"S, 49°38'9"W), 2♀ (IBSP 174768) all collected on 17–24/X/2008 by R. Andrade et al.; Cave GEM-2076, 2♀ 2010–11 (ISLA 14818); Cave SL_0002 (5°57'59"S, 49°38'59"W), 3♀ (ISLA 14806, ISLA 14797); Cave SL_0003 (5°57'51"S, 49°38'59"W), 1♀ (ISLA 14811); Cave SL_0004 (5°57'49"S, 49°38'59"W), 8♀ (ISLA 14786, ISLA 14783); Cave SL_0005 (5°57'49"S, 49°38'59"W), 1♀ (ISLA 14785); Cave SL_0009 (5°58'4"S, 49°38'59"W), 1♂ 1♀ (ISLA 14801); Cave SL_0016 (5°58'13"S, 49°38'52"W), 2♀ (ISLA 14804); Cave SL_0024 (5°58'16"S, 49°38'43"W), 1♂ 3♀ (ISLA 14787, ISLA 14798); Cave SL_0026 (5°58'21"S, 49°38'41"W), 5♀ (ISLA 14816); Cave SL_0029 (5°58'21"S, 49°38'37"W), 1♂ (ISLA 14810); Cave SL_0030 (5°58'21"S, 49°38'38"W), 5♀ (ISLA 14795, ISLA 148190); Cave SL_0033 (5°58'11"S, 49°38'37"W),, 1♀ (ISLA 14814); Cave SL_0035 (5°58'33"S, 49°38'17"W), 4♀ (ISLA 14822); Cave SL_0041 (5°58'37"S, 49°37'58"W), 1♀ (ISLA 14790); Cave SL_0042 (5°58'42"S, 49°37'57"W), 5♀ (ISLA 14807, ISLA 14796); Cave SL_0043 (5°58'36"S, 49°37'55"W), 2♀ (ISLA 14799); Cave SL_0045 (5°58'53"S, 49°38'9"W), 3♀ (ISLA 14820); Cave SL_0046 (5°58'56"S, 49°38'7"W), 1♀ (ISLA 14805); Cave SL_0050 (5°58'51"S, 49°37'52"W), 5♀ (ISLA 14794); Cave SL_0057 (5°58'36"S, 49°37'32"W), 8♀ (ISLA 14808); Cave SL_0060 (5°58'46"S, 49°37'22"W), 1♀ (ISLA 14803); Cave SL_0062 (5°58'46"S, 49°37'19"W), 3♀ (ISLA 14817, ISLA 14813); Cave SL_0067 (5°58'53"S, 49°37'14"W), 1♀ (ISLA 14793); Cave SL_0069 (5°59'3"S, 49°37'14"W), 3♀ (ISLA 14800); Cave SL_0071 (5°58'19"S, 49°37'29"W), 2♀ (ISLA 14809, ISLA 14802); Cave SL_0075 (5°57'56"S, 49°37'57"W), 1♂ 8♀ (ISLA 14788, ISLA 14789, ISLA 14784, ISLA 14791); Cave SL_0077 (5°58'0"S, 49°38'33"W), 1♀ (ISLA 14812); Cave SL_0079 (5°57'52"S, 49°38'18"W), 3♂ 5♀ (ISLA 14792); Cave SL_0086 (5°57'32"S, 49°38'10"W), 1♂ 1♀ (ISLA 14815); Cave SL_0092 (5°57'34"S, 49°38'39"W), 2♀ (ISLA 14821), all collected on 2010-2011 by Equipe Carste; Altamira, Cave Abrigo do Sismógrafo (3°18'0"S, 52°13'39"W), 1♀, 11/IV/2009 (IBSP 151442); Cave Abrigo Queda d’Água (3°15'18"S, 52°8'60"W), 8♂ 24♀, IV/2011 (IBSP 151391) collected by M. E. Bichuette coll.; Vitória do Xingu, Cave Abrigo Kararaô (3°8'33"S, 51°49'8"W), 2♀, XII/2010 (IBSP 151419); Cave Gruta do China (3°8'32"S, 51°49'8"W), 4♀, XII/2010 (IBSP 151404, IBSP 151407, IBSP 151409), all collected by M. E. Bichuette; São Geraldo do Araguaia, Cave SI-05 (4°58'35"S, 48°39'40"W), 1♂, 22/II–02/III/2011 (IBSP 175231); Cave SI-07 (6°22'2"S, 48°23'38"W), 1♂ 2♀, 22/II–02/III/2011 (IBSP 179480, IBSP 179481); Cave SI-08 (6°22'2"S, 48°23'38"W), 1♀, 22/II–02/III/2011 (IBSP 179482), all collected on 2010–2011 by F. P. Franco & B. F. Takano.

###### Etymology.

The specific name is a masculine noun in apposition and refers to the word *ferro*, which means iron in Portuguese. This name describes the type of lithology where the species is found in abundance.

###### Diagnosis.

Male resembles *P. novalima* sp. n. in the rounded tegulum, posterior portion of conductor hyaline and partially overlapping the embolus, and longer than larger bulb; it can be distinguished by the bifid conductor with a long and digitiform distal projection and rounded basal projection, and median apophysis with a single small tip (Fig. 1C–F). Female resembles *P. novalima* sp. n. in the large epigynal plate (Fig. 2B–C) and canoe-shaped sclerotized internal plate surrounding the spermathecae (Fig. 2C) but differs in the ample and rounded curvature of the copulatory ducts (Fig. 2C).

###### Description.

Male (Holotype, IBSP 173283). Cephalothorax brown, wider than longer. Sternum brown. Endite and labium slightly lighter than sternum. Chelicerae brown. Legs brown. Abdomen grayish, covered by dispersed bristles (Fig. 1A). Total length 1.85. Carapace 0.91 long. Clypeus 0.21 high. Sternum 0.45 long, 0.43 wide. Eye measurements: AME 0.07; ALE-PLE contiguous, 0.06 each; PME-PME separated by diameter of PME (0.07 each). Legs: I femur 0.99/ patella 0.39/ tibia 0.9/ metatarsus 0.6/ tarsus 0.25/ total 3.13. II 0.8/ 0.36/ 0.72/ 0.54/ 0.51/ 2.93. III 0.62/ 0.3/ 0.51/ 0.39/ 0.37/ 2.19. IV 0.7/ 0.3/ 0.57/ 0.42/ 0.37/ 2.36. Palp: pointed cymbium. Conductor robust, hyaline in the distal region, covering embolus tip. Embolus large, with two mesal embolic apophyses (see Coddington 1986: 12) pointing down, usually partially covered by hyaline membrane (Fig. 1C–G). Abdomen: 0.9 long.

Female (paratype IBSP 173283). Color and body as in male, except for the slightly longer than wide cephalothorax (Fig. 1B). Total length: 2.4. Carapace. 0.96 long. Sternum 0.56 long, 0.52 wide. Abdomen 1.5 long. Eye measurements: AME 0.08; ALE-PLE contiguous, 0.07 each; PME-PME separated by the diameter of PME (0.07 each). Clypeus 0.2 high. Legs I femur 1.2/ patella 0.45/ tibia 0.9/ metatarsus 0.68/ tarsus 0.51/ total 3.74. II 1/ 0.4/ 0.74/ 0.56/ 0.54/ 3.24. III 0.65/ 0.33/ 0.54/ 0.43/ 0.44/ 2.39. IV 0.95/ 0.35/ 0.7/ 0.53/ 0.44/ 2.97. Epigynal plate wider than long, posterior margin very sclerotized, with wrinkles near the posterior margin, covered by disperse bristles. Internally with glandular ducts in the middle of the curve of ducts. Ovoid spermathecae (Fig. 2A–C).

###### Variation.

Ten males: total length 1.75–1.95; carapace 0.75–0.91; femur I 0.9–1.15. 10 females: total length 2–2.8; carapace 0.9–1; femur I 1–1.25.

###### Distribution.

Canaã dos Carajás, Parauapebas, Curionópolis, Altamira and São Geraldo do Araguaia, Pará, Brazil (Fig. 16).

###### Natural history.

All specimens were collected in caves in iron formations. Most specimens were collected in the middle of orbicular webs, usually in the twilight zone (Fig. 3A–D). Egg sacs had a cubic shape and were fixed on cave walls (Fig. 3D). In some cases, more than four egg sacs were found in a single guide wire (Fig. 3C). The number of eggs per egg sac varied from eight to seventeen. They were recorded on different types of prey, and the most common prey items were winged species of orders Diptera (Fig. 3B) and Lepidoptera.

##### Plato striatus sp. n.

Taxon classificationAnimaliaAraneaeTheridiosomatidae

http://zoobank.org/9372DF43-4C6D-44A4-A2A4-D7A2C17C0E75

Figures 4 , 16

###### Types.

Male holotype from Cave GEM_1786 (6°06'19"S, 44°08'18"W), Parauapebas, Pará, Brazil, 07/II/2011, R. Zampaulo et al., deposited in IBSP 176982.

###### Etymology.

The specific name is a masculine noun in apposition and means “striped” in Latin, referring to the darkened stripes on the male`s fourth pair of legs.

###### Diagnosis.

**Figure 4.**
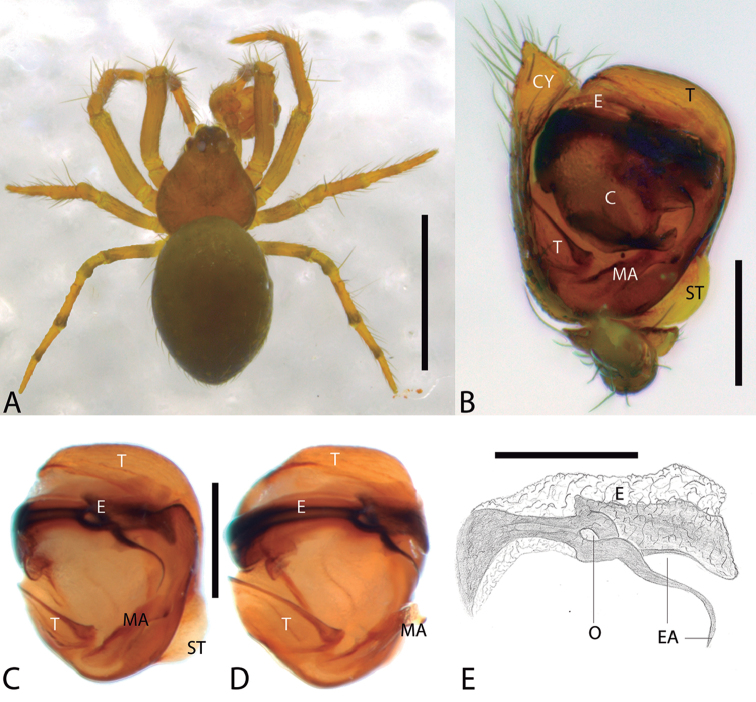
*Plato striatus* sp. n. **A** male, dorsal view **B** male palp, ventral view **C, D** male palp, ventral view with conductor removed **E** embolus, illustration. Abbreviations: **C** conductor; **CY** cymbium; **E** embolus; **EA** mesal embolic apophysis; **MA** median apophysis; **O** embolic opening; **ST** subtegulum; **T** tegulum. Scale bars: **A**, 0.8; **B–D** 0.1; **E** 0.05 mm.

*Plato striatus* sp. n. resembles *P. guacharo* in the twisted projection of the conductor (see Brignoli 1972: fig. 22), and *P. novalima* sp. n. and *P. ferriferus* sp. n. in the rounded tegulum, posterior portion of conductor hyaline and partially overlapping the embolus, and longer than large bulb. It differs from these three species in the proximal portion of the conductor simple, arrow-shaped, twisted and pointing upwards, embolus with proximal apophysis elongated, distal apophysis fused to the membrane (Fig. 4C–E) and fourth pair of legs with dark stripes on the articulations (Fig. 4A).

###### Description.

Male (Holotype, IBSP 176982). Cephalothorax brown, longer than wide. Sternum brown with darkened edges. Endite, labium, and chelicerae brown. Legs yellowish, stripes on articulations of the fourth pair. Abdomen dark grayish, longer than wide. Total length: 1.5. Carapace 0.6 long. Clypeus height: 0.2. Sternum: 0.3 long; 0.3 wide. Eye measurements: AME 0.1; ALE-PLE: grouped, 0.05 each; PME-PME: separated by 1 PME (0.07 each). Legs: I femur 0.6/ patella 0.21/ tibia 0.4/ metatarsus 0.3/ tarsus 0.28/ total 1.79; II 0.45/ 0.2/ 0.35/ 0.22/ 0.25/ 1.47; III 0.32/ 0.15/ 0.2/ 0.17/ 0.2/ 1.04; IIII 0.4/ 0.17/ 0.3/ 0.2/ 0.2/ 1.27. Palp: cymbium pointed; conductor covering most of the embolus. Embolus large, with rounded embolic opening between the mesal embolic apophysis (Fig. 4B–E; Coddington 1986: 12). Abdomen: 0.8 long.

Female. Unknown.

###### Distribution.

Known only from the type locality, a cave in Parauapebas, state of Pará, Brazil (Fig. 16).

##### Plato novalima sp. n.

Taxon classificationAnimaliaAraneaeTheridiosomatidae

http://zoobank.org/1A1653F3-0C7D-4C07-A000-FFE0F2DEDA7F

**Figure 5.**
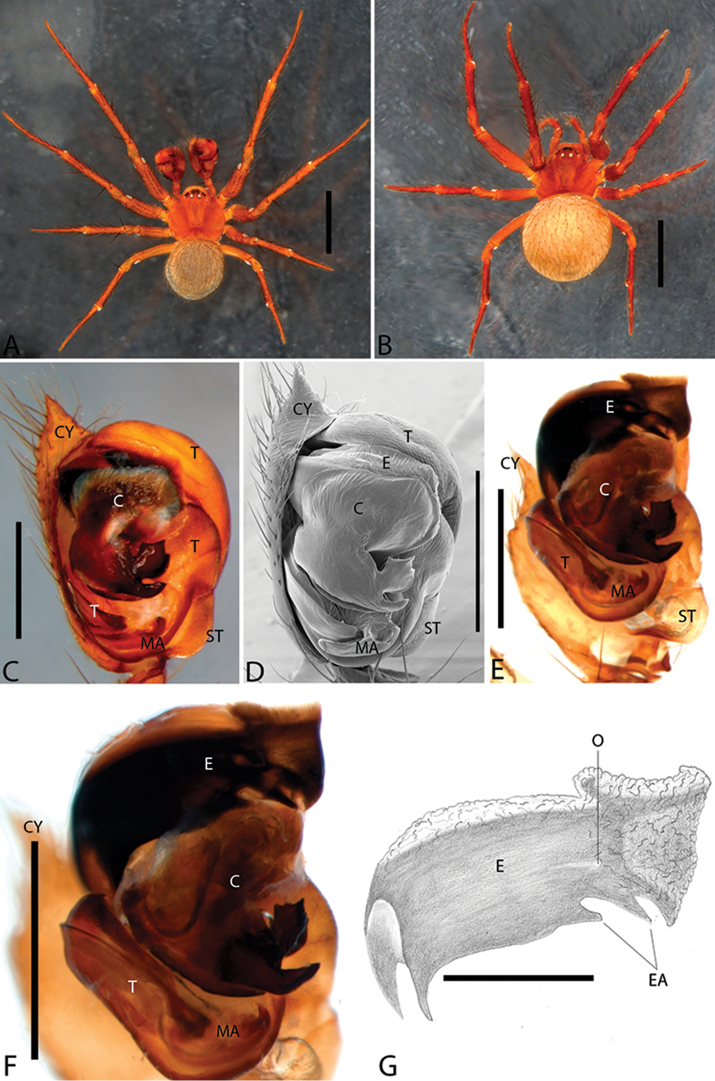
*Plato novalima* sp. n. **A** male, dorsal view **B** female, dorsal view **C** male palp, ventral view **D** male palp, ventral view (SEM) **E, F** male palp, ventral view with conductor removed **G** embolus, illustration. Abbreviations: **C** conductor; **CY** cymbium; **E** embolus; **EA** mesal embolic apophysis; **MA** median apophysis; **O** embolic opening; **ST** subtegulum; **T** tegulum. Scale bars **A, B** 1.8; **C–F** 0.45; **G** 0.21 mm.

**Figure 6.**
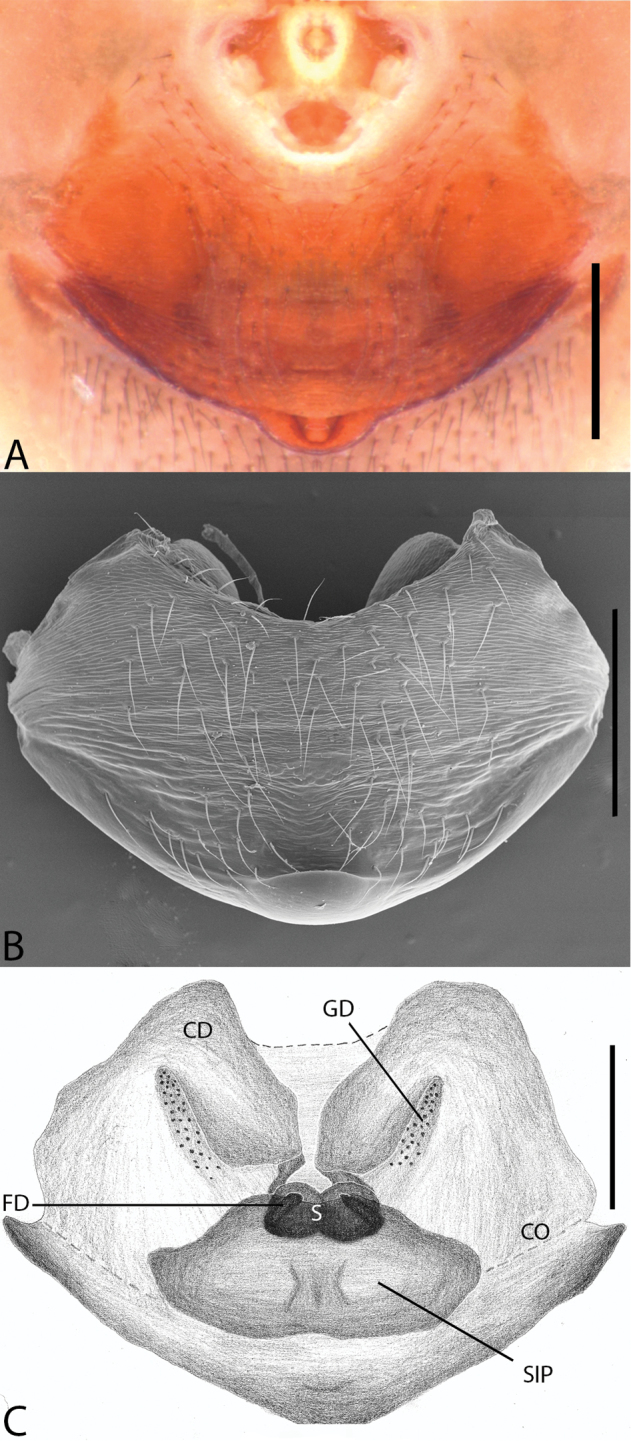
*Plato novalima* sp. n. **A**, female epigynum, ventral view **B** female epigynum, ventral view (SEM) **C** female epigynum, dorsal view, illustration. Abbreviations: **CD** copulatory ducts; **CO** copulatory opening; **FD** fertilization ducts; **GD** glandular ducts; **S** spermathecae; **SIP** sclerotized internal plate. Scale bars 0.3 mm.

Figures 3E , 5 , 6 , 16

Plato sp.: Pinto-da-Rocha, 1995: 74 (examined, now in IBSP 56068, IBSP 56075, and IBSP 56081).

###### Types.

Holotype: male from Cave VG_28 (20°07'00"S, 43°53'57"W), Nova Lima, Minas Gerais, Brazil, 29/III-1/IV/2011, V. Felice coll., deposited in IBSP 175196. Paratypes: male and female from Cave RF_15 (19°55'19"S, 43°29'43"W), Barão de Cocais, Minas Gerais, Brazil, 10–21/III/2009, R. Andrade coll. (MZSP70927); Male from Cave VG_28 (20°07'00"S, 43°53'57"W), Nova Lima, Minas Gerais, Brazil, 02-10/VIII/2011, Andrade et al. coll. (MZSP70926); Female from Cave VG_26 (20°07'00"S, 43°53'57"W), Nova Lima, Minas Gerais, Brazil, 29/III-01/IV/2011, G.P. Perroni coll. (MPEG 32024); Male from Cave VL_10 (20°17'9"S, 43°56'44"W), Itabirito, Minas Gerais, Brazil, 18-25/IV/2007, R. Andrade coll. (MPEG 32023); Female from Cave VL_09 (20°17'9"S, 43°56'47"W), Itabirito, Minas Gerais, Brazil, 18-25/IV/2007, R. Andrade coll. (MPEG 32025).

###### Other material examined.

BRAZIL. **Minas Gerais**: Barão de Cocais, Cave Mina Gongo Soco, 4♀ 02–24/XI/2007 (IBSP 127615, IBSP 127618) collected by R. Andrade coll.; Cave RF_05 (19°55'3"S, 43°29'10"W), 2♀ (IBSP 181157); 3♀, 10–21/III/2009 (IBSP 181144); Cave RF_07 (19°55'5"S, 43°29'13"W), 1♀ (IBSP 181134); Cave RF_09 (19°55'6"S, 43°29'13"W), 1♀ (IBSP 181137); Cave RF_13 (19°55'18"S, 43°29'28"W), 1♂ 1♀ (IBSP 181136); 4♀ (IBSP 181140); Cave RF_14 (19°55'19"S, 43°29'41"W), 1♀ (IBSP 181147); 2♀ (IBSP 181135); Cave RF_19 (19°55'19"S, 43°29'48"W), 1♀ (IBSP 181139, IBSP 181155); Cave RF_20 (19°55'21"S, 43°29'50"W), 1♀ 10–21/III/2009 (IBSP 181149); Cave RF_25 (19°55'26"S, 43°29'57"W), 1♀ (IBSP 181133); Cave RF_38 (19°55'42"S, 43°30'30"W), 3♂ 14♀ (IBSP 181146, IBSP 181132, IBSP 181138, IBSP 181142); Cave RF_46 (19°55'46"S, 43°30'36"W), 3♂ 8♀ (IBSP 181156, IBSP 181141); 2♀ (IBSP 181150); Cave RF_52 (19°56'5"S, 43°31'8"W), 1♂ 1♀ (IBSP 181145); Cave RF_55 (19°56'10"S, 43°31'23"W), 1♀ (IBSP 181151); Cave RF_57 (20°1'34"S, 43°41'8"W), 1♀ (IBSP 181153); Cave RF_59 (19°56'2"S, 43°31'58"W), 2♀ (IBSP 181152, IBSP 181158); Cave RF_72 (19°55'8"S, 43°28'19"W), 1♂ 14♀ (IBSP 181154, IBSP 181148), all collected on 10–21/III/2009 by R. Bessi et al.; Caeté; Cave AP_46 (20°2'28"S, 43°40'44"W), 1♀ (IBSP 175143); 3♀ (IBSP 175150, IBSP 175151); Cave AP_47 (20°1'39"S, 43°40'52"W), 3♀ (IBSP 175144–IBSP 175146) 4♀ (IBSP 175152–IBSP 175154); Cave AP_49 (20°2'8"S, 43°41'29"W), 1♀ (IBSP 175149); 1♂ 2♀ (IBSP 175175); Cave AP_55 (20°1'35"S, 43°40'47"W), 2♀ (IBSP 175148); 1♀ (IBSP 175156); Cave E_47 (20°2'10"S, 43_41'31"W), 1♀ (IBSP 175147); Cave E_71, 1♀ (IBSP 175142), all collected on 13–17/IV/2010 by R. Bessi et al.; Cave APOL_12 (20°0'11"S, 43°40'8"W), 1♀, 21–25/XI/2011 (IBSP 175169); 1♀, 30/VI–15/VII/2011 (IBSP 175166); Cave APOL_18 (20°3'7"S, 43°42'10"W), 1♀, 02–07/I/2012 (IBSP 175171); 1♀, 30/VI–15/VII/2011 (IBSP 175160); Cave APOL_29 (20°2'36"S, 43°41'56"W), 1♀, 30/VI–15/VII/2011 (IBSP 175161); Cave APOL_31 (20°0'11"S, 43°40'8"W), 1♀ 30/VI–15/VII/2011 (IBSP 175162); 2♀, 21–25/XI/2011 (IBSP 175170), all collected by G. P. Perroni et al.; Cordisburgo, Cave Morena (19°10'5"S, 44°20'20"W), 2♀ IV/1986, F. Chaimowicz coll. (IBSP 56075); 1♂ 12♀, 12–13/X/2007 (IBSP 132151, IBSP 132152, IBSP 132160, IBSP 132157, IBSP 132161), all collected by G. R.S. Ruiz & E. O. Machado; Curvelo, Cave Lapa do Mosquito (18°37'59"S, 44°24'37"W), 1♀, IV/1986 (IBSP 56068); 1♀, 30/XI/1992 (IBSP 56081) collected by F. Chaimowicz coll.; Itabirito, Cave VL_10 (20°17'9"S, 43°56'44"W), 1♂ 1♀, 18–25/IV/2007 (IBSP 115744,); Cave VL_12 (20°17'46"S, 43°56'47"W), 2♀, 18–25/IV/2007 (IBSP 115737); 3♀, 18–25/IV/2007 (IBSP 115740, IBSP 115756); 1♂ 12♀, 03–20/XI/2007 (IBSP 128821, IBSP 128844, IBSP 128822); 2♂ 2♀, IV/2008 (IBSP 128841, IBSP 128842), all collected by R. Andrade et al.; Lima Duarte, Parque Estadual de Ibitipoca, Cave Gruta dos Coelhos (19°5'11"S, 43°56'12"W), 3♀, 08/X/2002, F. Tulio coll. (IBSP 39723); Cave A, 3♀, 07/III/2006 (IBSP 117707); Cave B, 2♀, 07/III/2007 (IBSP 117702, IBSP 117698), all collected by M. E. Bichuette & F. P. Franco; Mariana, Cave CH_07 (20°14'35"S, 43°31'1"W), 4♀, 28–30/IV/2009 (IBSP 145929); Cave CH_20 (20°13'43"S, 43°31'4"W), 1♀, 28–30/IV/2009 (IBSP 146001); Cave CH_25 (20°13'37"S, 43°31'4"W), 1♀, 23–30/IX/2008 (IBSP 146031), all collected by F. P. Franco et al.; Cave FN_01 (20°13'39"S, 43°25'49"W), 9♀, 05–09/V/2008 (IBSP 146108, IBSP 146134, IBSP 146142); Cave FN_04 (20°13'18"S, 43°26'3"W), 1♀, 05–09/V/2009 (IBSP 146127); Cave FN_06 (20°13'7"S, 43°25'50"W), 4♂ 11♀, 05–09/V/2009 (IBSP 146154, IBSP 146156, IBSP 146165, IBSP 146171, IBSP 146161, IBSP 146169); Cave FN_09 (20°12'27"S, 43°26'19"W), 3♀, 16–21/I/2009 (IBSP 146185, IBSP 146188); Cave FN_10 (20°12'27"S, 43°26'19"W), 1♂ 2♀, 05–09/V/2009 (IBSP 146197); Cave FN_12 (20°12'29"S, 43°26'19"W), 1♂ 4♀, 16–21/I/2009 (IBSP 146218); Cave FN_13 (20°12'26"S, 43°26'18"W), 8♀, 05–09/V/2009 (IBSP 146225); Cave FN_14 (20°12'27"S, 43°25'58"W), 1♀, 05–09/V/2009 (IBSP 146247); Cave FN_23 (20°12'30"S, 43°26'22"W), 1♀, 01–02/IX/2010 (IBSP 175209); Cave FN_27 (20°13'25"S, 43°26'15"W), 1♀, 01–02/IX/2010 (IBSP 175210); 1♀, 03–07/II/2011 (IBSP 175211), all collected by R. Andrade et al.; Cave GS-12 (20°10'53"S, 43°31'8"W), 3♀ (IBSP 175177, IBSP 175178); Cave GS_19 (20°11'17"S, 43°30'31"W), 1♀ (IBSP 175176); Cave GS_22 (20°14'46"S, 43°28'48"W), 1♀ 06–16/VI/2011 (IBSP 175183); Cave GS_26 (20°13'0"S, 43°29'34"W), 1♀ (IBSP 175181); Cave GS_33 (20°12'31"S, 43°29'45"W), 1♀ (IBSP 175182); Cave GS_35 (20°10'47"S, 43°30'39"W), 1♂ 3♀ (IBSP 175179, IBSP 175180), all collected on 16/I–11/II/2011 by R. Bessi et al.; Matozinhos, Cave Gruta dos Irmãos Piriá (19°10'58"S, 44°6'35"W), 2♀, 30/XI/1992, F. Chaimowicz coll. (IBSP 56082); Cave MOC_70/71 (19°32'47"S, 44°0'50"W), 2♀, 28/VI–01/VII/2011, F. P. Franco & C. A. R. Souza et al. (IBSP 181131); Nova Lima, Cave VG_02 (20°9'30"S, 43°49'8"W), 1♀, 29/III–01/IV/2011 (IBSP 175187); Cave VG_07 (20°6'6"S, 43°53'44"W), 1♀, 29/III–01/IV/2011 (IBSP 175188); 2♀, 02–10/VIII/2011 (IBSP 175199); Cave VG_20 (20°7'24"S, 43°54'4"W), 1♀ 02–10/VIII/2011 (IBSP 175200); 2♀, 29/III–01/IV/2011 (IBSP 175189); Cave VG_26 (20°6'60"S, 43°53'55"W), 7♀, 02–10/VIII/2011 (IBSP 175203); 2♀, 29/III–01/IV/2011 (IBSP 175194, IBSP 175201, IBSP 175202); 6♀, 29/III–01/IV/2011 (IBSP 175191–IBSP 175193); Cave VG_28 (20°6'59"S, 43°53'56"W), 6♀, 02–10/VIII/2011 (IBSP 175206, IBSP 181130, IBSP 175205); 1♂ 2♀, 29/III–01/IV/2011 (IBSP 175195, IBSP 175197); Cave VG-40 (20°8'60"S, 43°52'28"W), 1♀, 02–10/VIII/2011 (IBSP 175208); 1♀, 29/III–01/IV/2011 (IBSP 175198), all collected by R. Andrade et al.; Pedro Leopoldo, Cave Gruta do Nei (19°37'45"S, 44°0'30"W), 4♀, 05–06/X/2009 (IBSP 175249, IBSP 175250); 2♀, 12–15/I/2009 (IBSP 175239, IBSP 175240), F. P. Franco et al.; Cave Gruta do Sufoco (19°37'46"S, 44°0'35"W), 10♀, 05–06/X/2009 (IBSP 175241–IBSP 175246, IBSP 175248); 2♂ 28♀, 12–15/I/2009 (IBSP 175235–IBSP 175238), all collected by F. P. Franco et al.; Rio Acima, Cave AP_09 (20°1'33"S, 43°40'54"W), 1♂ 4♀, 19–23/VII/2008 (IBSP 175215–IBSP 175217); Cave AP_10 (20°1'33"S, 43°40'55"W), 2♀, 19–23/VII/2008 (IBSP 175222); 1♂ 3♀, 14–21/XI/2008 (IBSP 175220); Cave AP_13 (20°1'40"S, 43°40'51"W), 2♀, 19–23/VII/2008 (IBSP 175218); Cave AP_15 (20°1'40"S, 43°40'52"W), 1♀, 19–23/VII/2008 (IBSP 175219); Cave AP_19 (20°1'43"S, 43°40'57"W), 1♀, 14–21/XI/2008 (IBSP 175221); Cave AP_21 (20°1'41"S, 43°40'53"W), 2♀ 14–21/XI/2008 (IBSP 175223, IBSP 175224); 1♂, 19–23/VII/2008 (IBSP 175214); Cave AP_36 (20°2'28"S, 43°40'44"W), 1♀, 14–21/XI/2008 (IBSP 175213); 2♀, 19–23/VII/2008 (IBSP 175212) all collected by R. Bessi et al.; Santa Bárbara, Cave APOL_10 (19°59'58"S, 43°39'55"W), 4♀, 21–25/XI/2011 (IBSP 175167, IBSP 175168); 9♀, 30/VI–15/VII/2011 (IBSP 175164, IBSP 175163); Cave APOL_11 (20°0'3"S, 43°39'57"W), 1♀, 30/VI–15/VII/2011 (IBSP 175165); Cave APOL_17 (20°3'9"S, 43°42'4"W), 1♀, 09–13/I/2012 (IBSP 175172); 3♀, 30/VI–15/VII/2011 (IBSP 175157–IBSP 175159); Cave SG_10 (20°3'17"S, 43°41'8"W), 2♀, 02–07/I/2012 (IBSP 175155, IBSP 175174); 3♀, 26–30/IX/2011 (IBSP 175173), all collected by G. P. Perroni et al.; Vazante, Cave P_04 (17°55'13"S, 46°48'37"W), 1♀ (IBSP 175185); 1♀ (IBSP 175184); Cave V_02 (17°55'36"S, 46°49'34"W), 1♀ (IBSP 175186), all collected on 16–19/VIII/2012 by A.P. Bueno.

###### Etymology.

The specific name is a noun in apposition from the type locality.

###### Diagnosis.

Male resembles *P. ferriferus* sp. n. and *P. striatus* sp. n. in the rounded tegulum, posterior portion of conductor hyaline and partially overlapping the embolus, and longer than larger bulb. It can be distinguished by the proximal portion of the conductor with an elongated tip and a prickly projection (Fig. 5C–F) and median apophysis with a single long rounded tip (Figs 5D). The female resembles *P. ferriferus* sp. n. in the larger than long epigynal plate, with a sclerotized canoe-shaped internal plate surrounding the spermathecae, but is distinguished by the smoothly projected posterior edge (Fig. 6A–B) and straight curvature of copulatory ducts (Fig. 6C).

###### Description.

Male (holotype IBSP 175196). Cephalothorax brown. Sternum dark brown, slightly longer than wide. Endite, labium, and chelicerae slightly darker than sternum. Legs brown. Abdomen ovoid, grayish, covered with bristles (Fig. 5A). Total length 2.1. Carapace 1.24 long. Clypeus: height 0.27. Sternum 0.66 long, 0.59 wide. Eye measurements: AME 0.09; ALE-PLE: grouped, 0.08 each; PME-PME: separated by diameter of 1 PME (0.08 each). Legs: I femur 1.55/ patella 0.65/ tibia 1.3/ metatarsus 1/ tarsus 0.78/ total 5.28. II 1.42/ 0.55/ 1.15/ 0.8/ 0.72/ 4.64. III 0.88/ 0.45/ 0.81/ 0.61/ 0.59/ 3.34. IV 1.15/ 0.45/ 0.94/ 0.64/ 0.52/ 3.7. Palp: cymbium pointed. Robust and sclerotized conductor, except posterior region, hyaline, covering the embolus. Embolus covered by the conductor. Flattened and darkened embolus, with rounded opening and two small mesal embolic apophyses (Fig. 5C–G; Coddington 1986:,12). Abdomen: 1.2 long.

Female (paratype IBSP 175204). Color as in male, except for lightly brown sternum and yellowish abdomen (Fig. 5B). Total length 2.79. Carapace 0.96 long. Clypeus: height: 0.28. Sternum 0.74 long, 0.68 wide. Eye measurements: AME 0.1. ALE-PLE: grouped, 0.09 each. PME-PME: separated by 0.1 (PME: 0.1 each). Legs: I femur 1.84/ patella 0.67/ tibia 1.39/ metatarsus 1.02/ tarsus 0.9/ total 5.82. II 1.6/ 0.68/ 1.25/ 0.9/ 0.86/ 5.29. III 1.25/ 0.45/ 0.88/ 0.65/ 0.67/ 3.9. IV 1.42/ 0.54/ 1.02/ 0.78/ 0.61/ 4.37. Epigynal plate large. Sclerotized posterior border, bristles disperse but concentrated in the median region. Internally, spermathecae ovoid, covered by sclerotized internal plate. Glandular ducts in the middle of copulatory duct (Fig. 6A–C). Abdomen: 1.75 long.

###### Variation.

10 males: total length: 2.1–2.5; carapace: 1.15–1.28; femur I: 1.55–1.75. 10 females: total length: 2.5–3.1; carapace: 0.96–1.45; femur I: 1.25–1.84.

###### Distribution.

Common in the Iron Quadrangle, state of Minas Gerais, Brazil (Fig. 16).

###### Natural history.

Most specimens were collected in caves in iron formations, in the middle of their orbicular webs and usually in the twilight zone (Fig. 3E). Egg sacs had a cubic shape and were fixed on the walls of caves. In some cases, more than one egg sac was observed on a single guide wire.

##### Cuacuba gen. n.

Taxon classificationAnimaliaAraneaeTheridiosomatidae

http://zoobank.org/3F30135D-3C0A-40D7-B90C-F848CAF87BA8

###### Type species.

*Cuacuba mariana* sp. n.

###### Etymology.

The generic name “*Cuacuba*” means *hidden* in the native language of the South American indigenous tribe Tupi. It refers to the embolus of the male palp.

###### Monophyly.

This genus has at least three putative synapomorphies: C-shaped conductor with a posterior apophysis covering the embolus (Fig. 11C–E), anterior apophysis with filamentous structures (Fig. 9D), and cuneiform tegulum (Fig. 9B).

###### Diagnosis.

Males of *Cuacuba* gen. n. can be distinguished from the other males in the family by the C-shaped conductor, split into anterior and posterior apophysis (Fig. 9B–C). The anterior apophysis has distal filamentous ornaments (Figs 9D, 14E) and the posterior one covers the embolus (Figs 9B, 11C–E). Resembles the genus *Sinoalaria* Zhao & Li, 2012 in the morphology of the conductor covering the embolus and in not having an embolic division (see Lin et al. 2014: fig. 6A). Females of *Cuacuba* gen. n. are similar to females of *Sinoalaria* in the curves of the copulatory ducts, and in position and shape of spermathecae (see Lin et al. 2014: figs 10A–B), but can be distinguished by the lack of scapus, and by having epigynum with salient posterior margin, distally notched in the transversal groove (Figs 10B, 15C–D).

###### Description.

**Figure 7.**
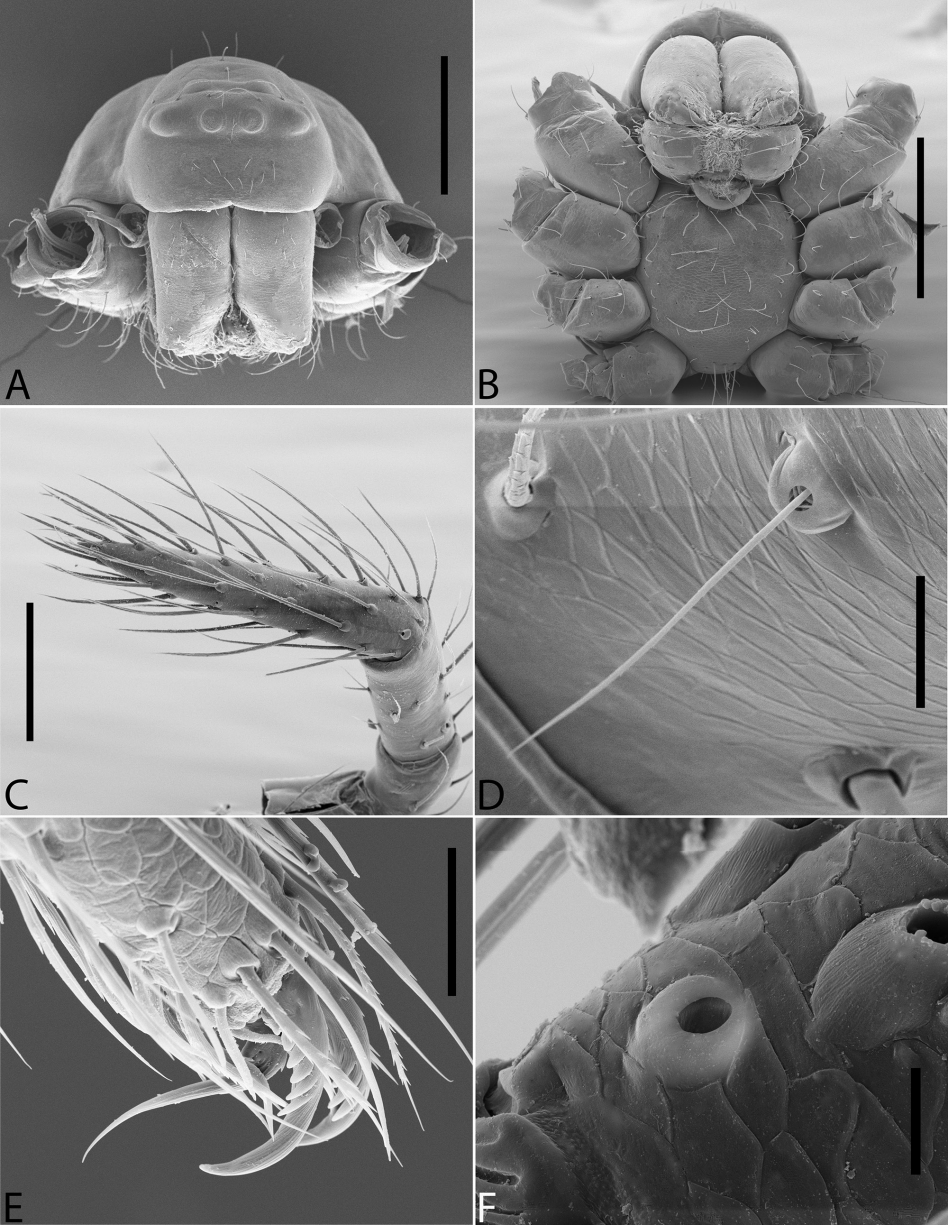
*Cuacuba mariana* sp. n. (IBSP 181212, 181214). **A** female prosoma, frontal view **B** female prosoma, ventral view **C** female left palp **D** detail of tibial trichobothria, female **E** detail of tarsal claws on leg 2, female **F** tarsal organ on leg 1, female. All SEM images. Scale bars: **A** 0.4; **B** 0.5; **C** 0.2; **D** 0.05; **E** 0.03; **F** 0.01 mm.

**Figure 8.**
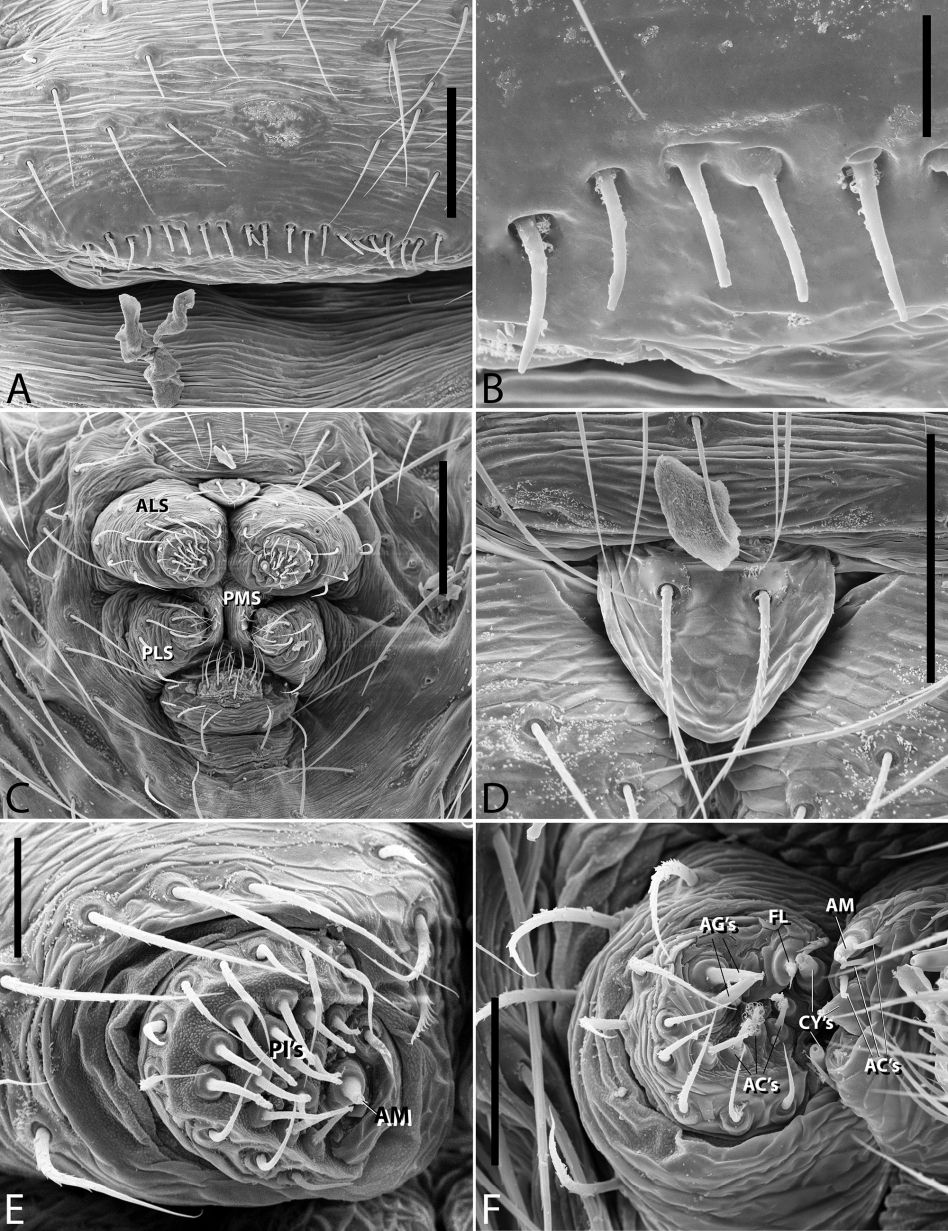
*Cuacuba mariana* sp. n. (IBSP 18118, 174911). **A** male epiandrous **B** male epiandrous, detail **C** male spinnerets **D** male colulus **E** male anterior lateral spinneret, detail **F** male posterior lateral spinneret and posterior median spinneret, detail. Abbreviations: **AC** aciniform spigots; **AG** aggregate gland spigots; **ALS** anterior lateral spinnerets; **AM** ampullate spigot; **CY** cylindrical gland spigots; **FL** flagelliform gland spigot; **PI** piriform gland spigots; **PLS** posterior lateral spinneret; **PMS** posterior median spinneret. Scale bars: **A** 0.05; **B** 0.01; **C** 0.1; **D** 0.05; **E** 0.02; **F** 0.03 mm.

**Figure 9.**
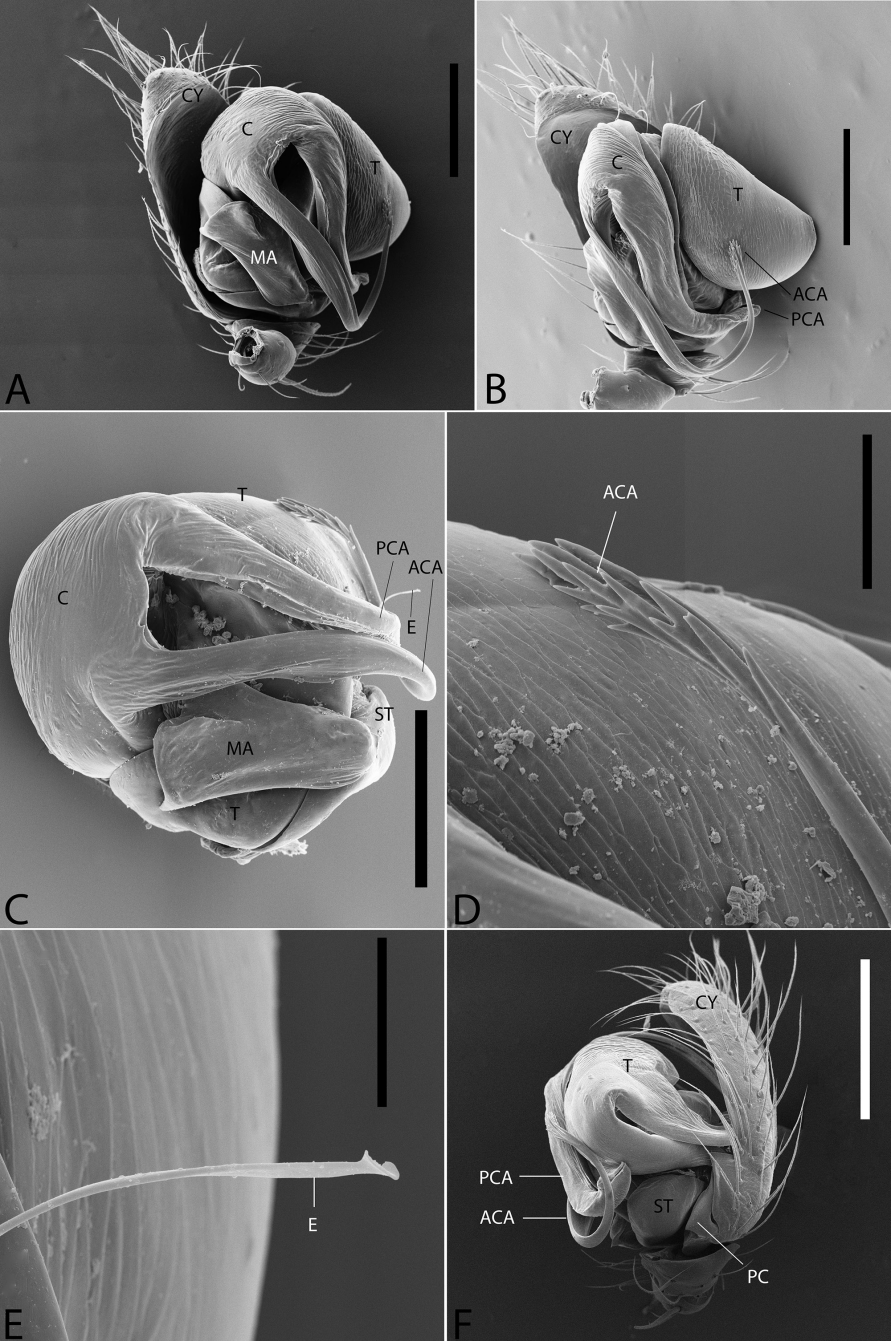
*Cuacuba mariana* sp. n. (IBSP 18118, 174911). **A** male palp, ventral view **B** male palp, ventral view **C** male palp, ventral view **D** anterior conductor apophysis tip, detail **E** posterior conductor apophysis tip, detail **F** male palp, retrolateral view. Abbreviations: **ACA** anterior conductor apophysis; **C** conductor; **CY** cymbium; **MA** median apophysis; **PC** paracymbium; **PCA** posterior conductor apophysis; **ST** subtegulum; **T** tegulum. Scale bars: **A–C**, 0.2; **D** 0.05; **E** 0.02; **F** 0.3 mm.

Total length 1.3–3 mm. Cephalothorax longer than wide, thoracic groove inconspicuous. Clypeus 3 times AME diameter. Eyes: anterior row recurved, posterior row straight. PME separated by its diameter. Sternum: rounded posteriorly. Legs long, formula: 1243. Tibia with a single row of 4-5 trichobothria, relatively short when compared to other genera (not as short as in *Wendilgarda*) (Fig. 7D). Tarsi with three claws. Paired claws with approximately five teeth. Unpaired claw long (Fig. 7E). Abdomen ovoid, larger than wide, with disperse and short bristles. Colulus a single flattened plate with a pair of bristles (Fig. 8D). Six spinnerets. Anterior lateral spinnerets larger than posterior lateral ones. Posterior median spinnerets smaller than others (Fig. 8C, E, F). Male palp with rounded cymbium (Fig. 9A–B). Paracymbium wing-shaped (Fig. 9F). Subtegulum transparent over fundus (Fig. 11D). Tegulum massive, scaled, and cuneiform, almost half the size of the bulb (Fig. 11C). Median apophysis large, conical, with dark tip (Figs 11D, 14F). Conductor C-shaped with posterior apophysis large, covering the embolus, and anterior apophysis narrow, generally with ornament-like accessory structures on the tip (Fig. 11C). Embolus long, flageliform, with large base, pars pendula, mesal embolic apophysis (Fig. 12A–B), and filamentous distal tip. Embolus covered by conductor up to the embolic opening (Fig. 12D), showing only tip of the embolus (Figs 11D–E, 14F–H). Female with epigynal plate sclerotized, without central pit, covered by short bristles, posterior margin triangular or rounded, distally notched (Figs 13A, 15A). Internally with long and coiled copulatory ducts, distally sclerotized, inserts laterally into the spermathecae, basal area with glandular ducts (Figs 13C–D, 15B, E). Two basal oval and connate spermathecae, longer than fertilization ducts (Figs 13C–D, 15B, D, E).

**Figure 10.**
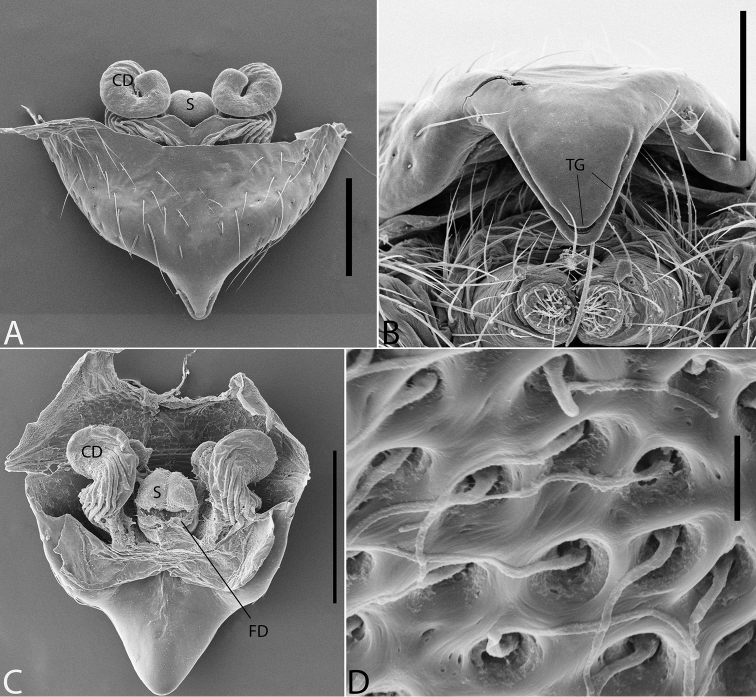
*Cuacuba mariana* sp. n. (IBSP 181212, 181214) **A** female epigynum, ventral view **B** female epigynum, detail of posterior margin **C** female epigynum, dorsal view **D** detail of glandular ducts. Abbreviations: **CD** copulatory ducts; **TG** transversal groove; **FD** fertilization ducts; **S** spermathecae. Scale bars: **A** 0.2; **B** 0.15; **C** 0.3; **D** 0.01 mm.

**Figure 11.**
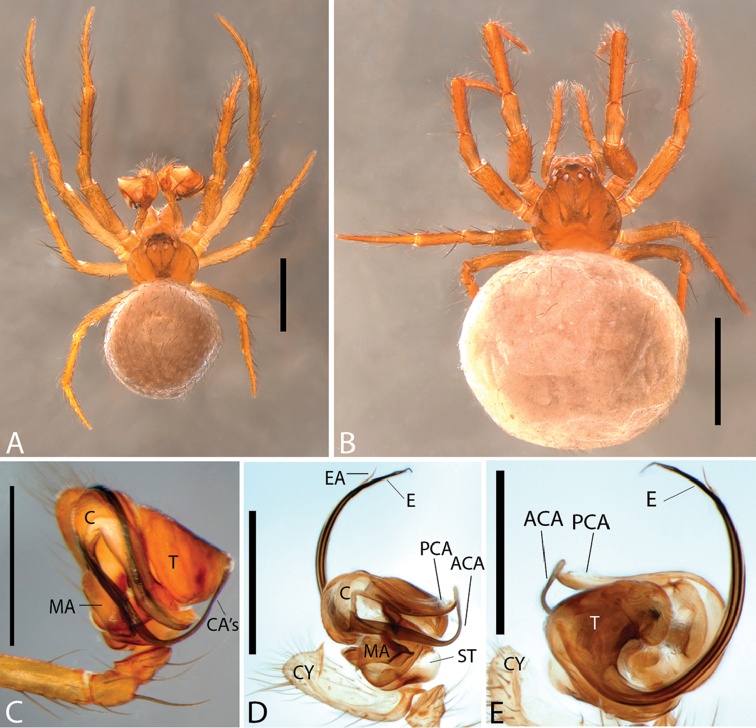
*Cuacuba mariana* sp. n. **A** male, dorsal view **B** female, dorsal view **C** male palp, ventral view **D** male palp, expanded, retrolateral view **E** male palp, expanded, dorsal view. Abbreviations: **ACA** anterior conductor apophysis; **C** conductor; **CA** conductor apophysis; **CY** cymbium; **E** embolus; **EA** mesal embolic apophysis; **MA** median apophysis; **PCA** posterior conductor apophysis; **ST** subtegulum; **T** tegulum. Scale bars: **A** 0.9; **B** 1.1; **C–E** 0.2 mm.

**Figure 12.**
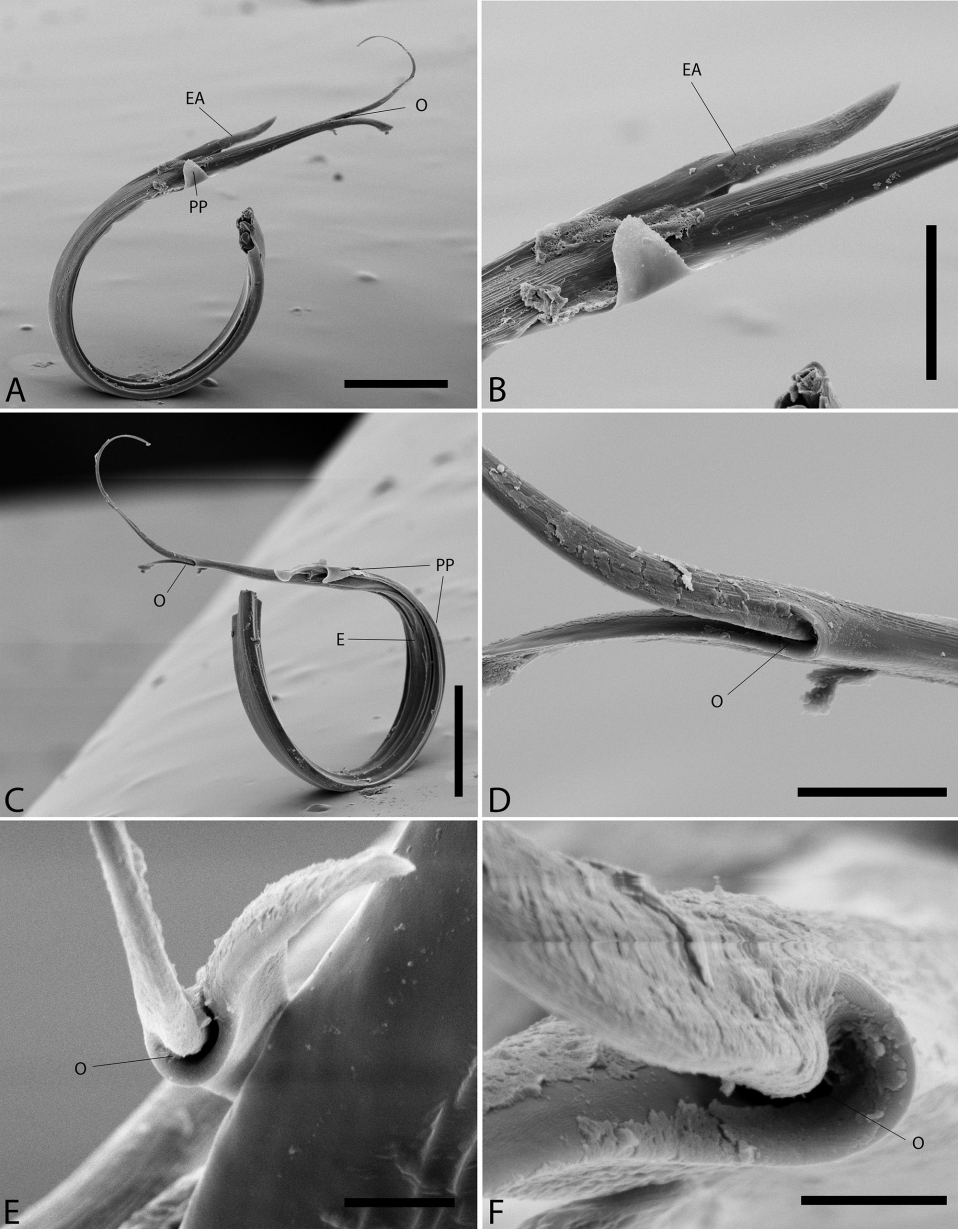
*Cuacuba mariana* sp. n. **A** embolus, cut out **B** same, detail of embolic apophysis **C** same, cut out **D** same, detail of embolic opening **E** same, detail of embolic opening **F** same, detail of embolic opening. Abbreviations: **E** embolus; **EA** embolic apophysis; **O** embolic opening; **PP** pars pendula. Scale bars: **A** 0.1; **B** 0.05; **C** 0,1 **D** 0.02; **E** 0.01; **F** 0.005 mm.

**Figure 13.**
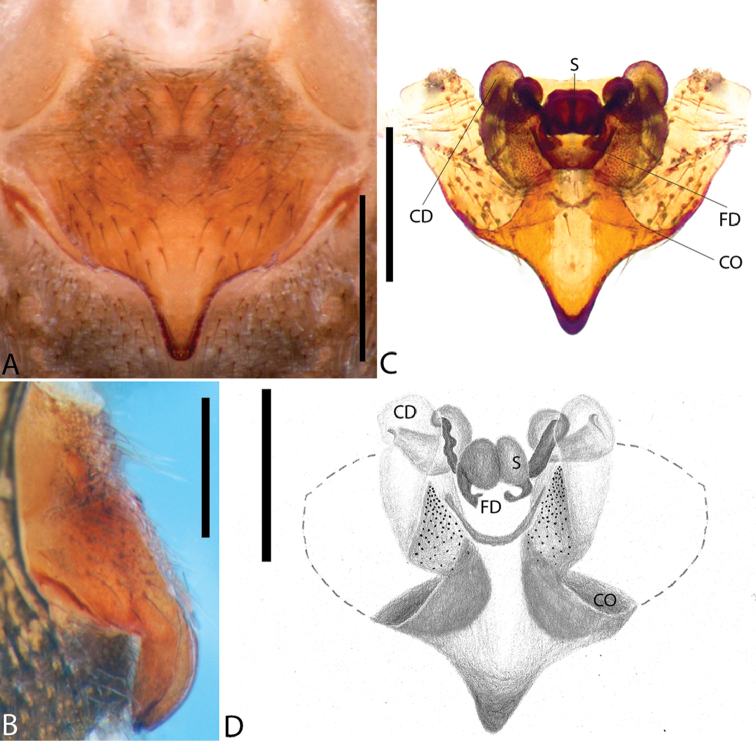
*Cuacuba mariana* sp. n. **A** female epigynum, ventral view **B** female epigynum, lateral view **C** female epigynum, dorsal view, clarified **D** female epigynum, dorsal view, illustration. Abbreviations: **CD** copulatory ducts; **CO** copulatory opening; **FD** fertilization ducts; **S** spermathecae. Scale bars: **A, C, D** 0.27; **B** 0.22 mm.

###### Natural history.

*Cuacuba* gen. n. was found only in caves. The species is a troglophile, as its specimens do not lack pigmentation nor have modified eyes (Fig. 3F).

##### Cuacuba mariana sp. n.

Taxon classificationAnimaliaAraneaeTheridiosomatidae

http://zoobank.org/E7F94F33-4051-4788-B113-59E7D00A3C34

Figures 3F , 7 , 8 , 10 , 11 , 12 , 13 , 17

Plato sp.: Pinto-da-Rocha 1995: 74 (now in IBSP 56084).

###### Types.

Male holotype from Cave SG_07 (20°02'58"S, 43°41'4"W), Santa Bárbara, Minas Gerais, Brazil, 26-30/IX/2011, Equipe Carste, deposited in IBSP 175141. Paratypes: male and female from Cave ICPA_697 (20°22'20"S, 45°36'14"W), Pains, Minas Gerais, Brazil, 26-30/IX/2011, A. P. Bueno et al coll. (MZSP 70925); male from Cave MOC_10 (19°33'6"S, 44°1'11"W), Matozinhos, Minas Gerais, Brazil, 28/VI–01/VII/2011, C.A.R. Souza et al. coll. (MPEG 32022); female from Cave MOCN_10 (19°33'19"S, 44°00'56"W), Matozinhos, Minas Gerais, Brazil, 04–15/IV/2011, C.A.R. Souza et al. coll. (MPEG 32021).

###### Other material examined.

BRAZIL. **Minas Gerais**: Mirabela, Cave Gruta Vaca Voadora, 1♀, VII/1984, F. Chaimowicz coll. (IBSP 3810); Montes Claros, Cave Lapa Encantada (16°19'48"S, 43°59'24"W), 1♀, X/1985 (IBSP 56084), F. Chaimowicz coll.; Lima Duarte, Cave Gruta dos Manequins, 3♀, X–XI/2000 (IBSP 39768), F. Tulio coll.; Cordisburgo, Cave Morena (19°10'7"S, 44°20'21"W), 1♀, 12/IX/2001 (IBSP 71835), R. L. Ferreira coll.; Mariana, Cave CH_07 (20°14'35"S, 43°31'1"W), 1♂ 5♀, 23–30/IX/2008 (IBSP 145930); Cave CH_17 (20°14'30"S, 43°31'6"W), 1♀, 28–30/IV/2009 (IBSP 145977); Cave CH_18 (20°14'30"S, 43°31'7"W), 1♂ 2♀, 23–30/IX/2008 (IBSP 145988); Cave CH_19 (20°14'32"S, 43°30'59"W), 1♀, 28–30/IV/2009 (IBSP 145994); 4♀, 23–30/IX/2008 (IBSP 145997); Cave CH_20 (20°13'43"S, 43°31'4"W), 2♀, 23–30/IX/2008 (IBSP 146002); Cave CH_21 (20°13'55"S, 43°31'5"W), 1♂ 2♀, 23–30/IX/2008 (IBSP 146009, IBSP 146010); 3♀, 28–30/IV/2009 (IBSP 146012); Cave CH_22 (20°13'55"S, 43°31'5"W), 1♀, 28–30/IV/2009 (IBSP 146023), all collected by F. P. Franco *et al.*; Cave GS_03 (20°9'16"S, 43°30'58"W), 2♀, 05/I/2011 (IBSP 174909, IBSP 174910); Cave GS_06 (20°10'10"S, 43°31'0"W), 1♀, 16/I–11/II/2011 (IBSP 174911); 1♀, 06–16/VI/2011 (IBSP 174912); Cave GS_09 (20°10'10"S, 43°31'0"W), 1♀, 06–16/VI/2011 (IBSP 174913); Cave GS_10 (20°10'6"S, 43°30'57"W), 2♀, 06–16/VI/2011 (IBSP 174914) all collected by R. Bessi *et al.*; Cave Gruta Furnas I (20°12'36"S, 43°12'36"W), 2♀, IX/2002 (IBSP 39733); 1♀, IX/2002 (IBSP 39734); 1♀, 14/VII/2002 (IBSP 39772), all collected by F. Tulio; Piumhi, Cave ICCA_014 (20°20'44"S, 45°50'53"W), 1♂ (IBSP 181216); Cave ICCA_018 (20°20'44"S, 45°50'54"W), 1♂ 3♀ (IBSP 181201, IBSP 181212, IBSP 181231); Cave ICCA_020 (20°20'44"S, 45°50'56"W), 1♂, 13–21/I/2014 (IBSP 181217); Cave ICCA_022 (20°20'42"S, 45°50'55"W), 1♂ 2♀ (IBSP 181227, IBSP 181230); Cave ICCA_026 (20°20'44"S, 45°50'56"W), 1♂ (IBSP 181205); Cave ICCA_035 (20°20'45"S, 45°50'55"W), 1♂ 2♀ (IBSP 181199, IBSP 181229); Cave ICCA_037 (20°20'45"S, 45°50'55"W), 1♀ (IBSP 181226); Cave ICCA_041 (20°20'46"S, 45°50'53"W), 1♂ 2♀, 4–22/XI/2013 (IBSP 181208, IBSP 181233); Cave ICCA_046 (20°20'50"S, 45°50'54"W), 1♀ (IBSP 181200); Cave ICCA_052 (20°20'48"S, 45°50'57"W), 2♀ (IBSP 181228, IBSP 181235); Cave ICCA_053 (20°20'49"S, 45°50'56"W), 1♀ (IBSP 181223); Cave ICCA–068 (20°20'50"S, 45°50'52"W), 1♀ (IBSP 181237); Cave ICCA_072 (20°20'51"S, 45°50'54"W), 1♂ (IBSP 181209); Cave ICCA–079 (20°20'52"S, 45°50'52"W), 1♂ 1♀ (IBSP 181220); Cave ICCA_081 (20°20'52"S, 45°50'52"W), 2♀ (IBSP 181211, IBSP 181213); Cave ICCA_082 (20°20'51"S, 45°50'55"W), 1♀ (IBSP 181210); Cave ICCA_103 (20°20'54"S, 45°50'46"W), 1♂ (IBSP 181196); Cave ICCA_129 (20°21'3"S, 45°50'35"W), 3♀, 4–22/XI/2013 (IBSP 181203, IBSP 181219, IBSP 181232); Cave ICCA_139 (20°20'56"S, 45°50'42"W), 1♂ 3♀, 4–22/XI/2013 (IBSP 181198, IBSP 181207, IBSP 181224); Cave ICCA–141 (20°20'53"S, 45°50'42"W), 2♀ (IBSP 181202); Cave ICCA_142 (20°20'54"S, 45°50'36"W), 1♂ 1♀ (IBSP 181214, IBSP 181236); Cave ICCA_158 (20°20'59"S, 45°50'35"W), 1♀, 13–21/I/2014 (IBSP 181218); Cave ICCA_187 (20°21'3"S, 45°50'34"W), 1♀ (IBSP 181239); Cave ICCA_208 (20°20'59"S, 45°50'41"W), 1♂ (IBSP 181234); Cave ICCA–209 (20°21'3"S, 45°50'27"W), 1♂ 1♀ (IBSP 181225); Cave ICCA_211 (20°21'2"S, 45°50'27"W), 2♀ (IBSP 181222); Cave ICCA_247 (20°21'3"S, 45°50'23"W), 1♀, 13–21/I/2014 (IBSP 181206); Cave ICCA_353 (20°21'5"S, 45°50'18"W), 1♀ (IBSP 181221); Cave ICCA_355 (20°21'6"S, 45°50'21"W), 1♂ (IBSP 181238); Cave ICCA–531 (20°20'58"S, 45°50'26"W), 1♂ (IBSP 181215); Cave ICCA_533 (20°20'58"S, 45°50'26"W), 2♀ (IBSP 181195); Cave ICCA_566 (20°20'42"S, 45°50'55"W), 1♀, 13–21/I/2014 (IBSP 181194); Cave ICCA_568 (20°20'50"S, 45°50'56"W), 1♀, 13–21/I/2014 (IBSP 181197); Cave ICCA_574 (20°21'6"S, 45°50'20"W), 1♀ (IBSP 181204), all collected between 4–22/XI/2013 by A. P. Bueno *et al.*; Pains, Cave Ressurgência da Loca D’Água (20°25'48"S, 45°41'24"W), 9♀, 07/IX/2001 (IBSP 71854, IBSP 71856), P. Gnaspini coll.; Cave Gruta do Topo (20°21'36"S, 45°40'12"W), 1♀, 01/XI/2005 (IBSP 71901) M.E. Bichuette coll.; Cave Gruta Arcaica, 2♀, 25/I/2008 (IBSP 118679); Cave Gruta Sem Fim, 7♂ 10♀, 24–25/I/2008 (IBSP 118670, IBSP 118673, IBSP 118693, IBSP 118682, IBSP 118698, IBSP 118705), E.O. Machado & J.P.P.P. Barbosa coll.; Cave ICPA_031 (20°22'37"S, 45°36'32"W), 1♂ (IBSP 181163); Cave ICPA_041 (20°22'12"S, 45°36'23"W), 1♀ (IBSP 181159); Cave ICPA_144 (20°22'17"S, 45°36'39"W), 2♀, 24–27/IV/2012 (IBSP 174926); 1♀, 28–29/XI/2012 (IBSP 174928); Cave ICPA_146 (20°22'16"S, 45°36'42"W), 1♀, 28–29/XI/2012 (IBSP 174927); Cave ICPA_631 (20°22'25"S, 45°36'16"W), 1♀ (IBSP 181160); Cave ICPA_636 (20°22'14"S, 45°36'10"W), 1♀ (IBSP 181189); Cave ICPA_639 (20°22'14"S, 45°36'7"W), 2♀ (IBSP 181167); Cave ICPA_665 (20°22'25"S, 45°36'17"W), 1♀ (IBSP 181188); Cave ICPA_671 (20°22'15"S, 45°36'18"W), 1♀ (IBSP 181179); Cave ICPA_685 (20°22'39"S, 45°36'29"W), 1♀ (IBSP 181170); Cave ICPA_692 (20°22'11"S, 45°36'17"W), 2♀ (IBSP 181171, IBSP 181184); Cave ICPA_693 (20°22'14"S, 45°36'10"W), 1♀ (IBSP 181191); Cave ICPA_697 (20°22'20"S, 45°36'14"W), 2♀ (IBSP 181172, IBSP 181175); Cave ICPA_699 (20°22'25"S, 45°36'11"W), 3♀(IBSP 181177, IBSP 181175); Cave ICPA_700 (20°22'24"S, 45°36'29"W), 1♂ (IBSP 181193); Cave ICPA_710 (20°22'37"S, 45°36'15"W), 1♂ (IBSP 181161); Cave ICPA_711 (20°22'27"S, 45°36'19"W), 1♀ (IBSP 181176); Cave ICPA_731 (20°22'29"S, 45°36'31"W), 3♀ (IBSP 181162, IBSP 181165); Cave ICPA_731 (20°22'29"S, 45°36'31"W), 1♀ (IBSP 181165); Cave MV_014 (20°22'22"S, 45°36'11"W), 2♀ (IBSP 181185, IBSP 181186); Cave MV_018 (20°22'26"S, 45°36'14"W), 1♂ (IBSP 181180); Cave MV_019 (20°22'27"S, 45°36'14"W), 1♀ (IBSP 181168); Cave MV_025 (20°22'22"S, 45°36'14"W), 1♀ (IBSP 181169); Cave MV_027 (20°22'25"S, 45°36'13"W), 1♀ (IBSP 181187); Cave MV_036 (20°22'24"S, 45°36'11"W), 1♀ (IBSP 181192); Cave MV_037 (20°22'25"S, 45°36'12"W), 1♀ (IBSP 181181); Cave MV–044 (20°22'17"S, 45°36'9"W), 2♀ (IBSP 181174); Cave MV_090 (20°22'28"S, 45°36'27"W), 1♀ (IBSP 181166); Cave MV_16 (20°22'25"S, 45°36'11"W), 1♀ (IBSP 181190); Cave MV_27 (20°22'25"S, 45°36'13"W), 1♂ (IBSP 181178); Cave MV_33 (20°22'17"S, 45°36'16"W), 2♂ (IBSP 181183); Cave MV_44 (20°22'17"S, 45°36'9"W), 1♀ (IBSP 181164), all collected on 18/II–9/III/2013 by A. P. Bueno *et al.*; Matozinhos, Cave MOC_113/114 (19°33'18"S, 44°1'13"W), 2♀, 04–15/IV/2011 (IBSP 174831, IBSP 174832); Cave MOC_118 (19°32'53"S, 44°1'4"W), 2♀, 04–15/IV/2011 (IBSP 174835, IBSP 174836); Cave MOC_120 (19°33'5"S, 44°1'7"W), 2♀, 04–15/IV/2011 (IBSP 174839); 1♀, 29–30/VIII/2011 (IBSP 174852); Cave MOC_123 (19°33'13"S, 44°1'9"W), 2♀, 04–15/IV/2011 (IBSP 174840); Cave MOC_131 (19°33'2"S, 44°0'49"W), 2♀, 04–15/IV/2011 (IBSP 174833, IBSP 174834); Cave MOC_133 (19°33'3"S, 44°0'49"W), 2♀, 04–15/IV/2011 (IBSP 174841); Cave MOC_135 (19°33'16"S, 44°0'20"W), 2♂ 3♀, 04–15/IV/2011 (IBSP 174842, IBSP 174843, IBSP 174853); Cave MOC_16 (19°33'9"S, 44°1'5"W), 2♀, 08–18/II/2011 (IBSP 174818); Cave MOC_17 (19°33'9"S, 44°1'6"W), 1♀, 08–18/II/2011 (IBSP 174819); Cave MOC_18 (19°33'10"S, 44°1'6"W), 1♀, 08–18/II/2011 (IBSP 174820); Cave MOC_19 (19°33'7"S, 44°1'13"W), 16♀, 08–18/II/2011 (IBSP 174821–IBSP 174825); 1♂ 5♀, 28/VI–01/VII/2011 (IBSP 174846–IBSP 174848); Cave MOC_30 (19°33'2"S, 44°1'6"W), 1♂ 1♀, 08–18/II/2011 (IBSP 174829, IBSP 174830); Cave MOC_32 (19°33'12"S, 44°1'4"W), 1♂ 6♀, 08–18/II/2011 (IBSP 174826–IBSP 174828); 4♀, 28/VI–01/VII/2011 (IBSP 174849, IBSP 174850); Cave MOC_N8 (19°33'18"S, 44°0'59"W), 3♀, 04–15/IV/2011 (IBSP 174837, IBSP 174838); 1♀, 01–08/VIII/2011 (IBSP 174851), all collected by C. A. R. Souza *et al.*; Cave Gruta Periperi II (19°31'12"S, 44°3'36"W), 2♀, 26/XI/1992 (IBSP 56071); Cave Gruta do Tombo (19°30'0"S, 44°0'36"W), 1♂ 3♀, 29/XI/1992 (IBSP 56067), F. Chaimowicz coll.; Morro do Pilar, Cave MP_01A (19°9'15"S, 43°24'13"W), 14♀, 12–24/IX/2011 (IBSP 174864–IBSP 174867, IBSP 174869–IBSP 174873); 2♂ 8♀, 28/II/2012 (IBSP 174891–IBSP 174898,); Cave MP_01B (19°9'15"S, 43°24'13"W), 16♀, 28/II/2012 (IBSP 174899–IBSP 174908); 1♂ 9♀, 12–24/IX/2011 (IBSP 174868, IBSP 174874–IBSP 174876, IBSP 174878, IBSP 174879, IBSP 174881); 4♀, 12–24/IX/2011 (IBSP 174877, IBSP 174880); Cave MP_10 (19°9'28"S, 43°23'29"W), 2♀, 13–17/II/2012 (IBSP 174882); Cave MP_13 (19°7'5"S, 43°26'1"W), 4♀, 12–24/IX/2011 (IBSP 174854–IBSP 174856); 1♀, 13–17/II/2012 (IBSP 174883); Cave MP_14 (19°8'15"S, 43°24'19"W), 5♀, 13–17/II/2012 (IBSP 174885–IBSP 174887); 7♀, 12–24/IX/2011 (IBSP 174857–IBSP 174861); Cave MP_18 (19°10'14"S, 43°23'47"W), 1♀, 13–17/II/2012 (IBSP 174888); Cave MP_19 (19°9'45"S, 43°23'29"W), 3♀, 13–17/II/2012 (IBSP 174889); Cave MP_20 (19°10'2"S, 43°23'37"W), 3♀, 12–24/IX/2011 (IBSP 174862, IBSP 174863); Cave MP_21 (19°8'56"S, 43°25'45"W), 1♀, 13–17/II/2012 (IBSP 174890); Santa Barbara, Cave SG_02 (20°2'56"S, 43°40'56"W), 9♀, 05–09/XII/2011 (IBSP 174920–IBSP 174923); 9♀, 26–30/IX/2011 (IBSP 174916–IBSP 174918, IBSP 175225); Cave SG_07 (20°2'58"S, 43°41'4"W), 3♀, 05–09/XII/2011, (IBSP 174924, IBSP 174925), all collected by I. Cizauskas *et al*.; Cave SPD_38 (20°3'43"S, 43°40'14"W), 1♀, 09–13/I/2012, J. Mascarenhas coll. (IBSP 174915).

###### Etymology.

The specific name is a feminine noun in apposition and refers to one of the cities where the species is found. This city suffered one of the worst mining accidents in Brazilian history, in 2015.

###### Diagnosis.

Males of *Cuacuba mariana* sp. n. differ from *C. morrodopilar* sp. n. in the distal area of the anterior conductor apophysis with a group of thorns (Fig. 9B, D) and median apophysis without protruding tips (Figs 9A, C, 11D). Females can be distinguished by the conical salient posterior margin of the epigynal plate, with accentuate curvature in the distal region (Fig. 13A–B).

###### Description.

Male (holotype IBSP 175141). Cephalothorax yellowish brown. Chelicerae, endites, labium, and sternum red to brown. Legs orange. Abdomen grayish. Total length 2.25. Carapace 0.82 long. Clypeus high 0.26. Sternum 0.47 long, 0.48 wide. Eye measurements: AME 0.07; ALE-PLE: grouped, 0.07 each; PME-PME: separated by diameter of 1 PME (0.06 each). Legs: I femur 0.92/ patella 0.39/ tibia 0.82/ metatarsus 0.64/ tarsus 0.47/ total 3.25. II 0.9/ 0.35/ 0.72/ 0.52/ 0.45/ 2.94. III 0.63/ 0.27/ 0.45/ 0.38/ 0.35/ 2.08. IV 0.75/ 0.3/ 0.59/ 0.43/ 0.34/ 2.41. Palp: short median apophysis (Fig. 11D), anterior conductor apophysis longer and thinner than posterior one (Fig. 11C). Abdomen: 1.4 long.

Female (paratype IBSP 174919). Coloration as in male, except for brown cephalothorax and legs. Total length 3. Carapace 0.94 long. Clypeus high 0.21. Sternum 0.56 long, 0.58 wide. Eye measurements: AME 0.07; ALE-PLE: grouped, 0.07 each; PME-PME: separated by 0.07 (PME: 0.07 each). Legs: I femur 1.16/ patella 0.46/ tibia 0.82/ metatarsus 0.64/ tarsus 0.57/ total 3.65. II 1.15/ 0.45/ 0.81/ 0.59/ 0.55/ 3.55. III 0.68/ 0.32/ 0.52/ 0.43/ 0.44/ 2.39. IV 0.92/ 0.37/ 0.68/ 0.52/ 0.47/ 2.96. Epigynal plate with posterior margin darkened at the tip (Fig. 13A, C). Epigynum with glandular ducts in the basal third of the copulatory ducts, spermathecae connate, and fertilization ducts smaller than spermathecae (Fig. 13C–D). Abdomen: 2.57 long.

###### Variation.

Five males: total length 1.8–2.25; carapace 0.82–1; femur I: 0.92–1.2. 8 females: total length 2–3; carapace 0.8–1.15; femur I: 1–1.3.

###### Distribution.

Found in caves near the Iron Quadrangle, state of Minas Gerais, Brazil (Fig. 17).

##### Cuacuba morrodopilar sp. n.

Taxon classificationAnimaliaAraneaeTheridiosomatidae

http://zoobank.org/709CAEE8-D109-4A9A-BC3D-0BF2DCA64054

Figs 14 , 15 , 17

###### Types.

Male holotype and female paratype from Cave MP_07 (19°15'30"S, 43°22'35"W), Morro do Pilar, Minas Gerais, Brazil, 12-24/IX/2011, Equipe Carste coll., deposited in IBSP 174389; Paratypes: male and female (IBSP 174387); one female (MZSP 70928); one female (MPEG 32026), 13-17/II/2012, all with same of data of holotype.

###### Other material examined.

Brazil, Minas Gerais: Morro do Pilar, Cave MP_06 (19°14'58"S, 43°21'09"W), 3♀, 12-24/IX/2011 (IBSP 174383-174384); 3♀, 12-24/IX/2011, J. Mascarenhas coll. (IBSP 174385, IBSP 174394); 1♂ 3♀, 13-17/II/2012 (IBSP 174395, IBSP 174400); Cave MP_07 (19°15'30"S, 43°22'35"W), 2♂ 8♀, 12-24/IX/2011 Equipe Carste coll. (IBSP 174387–IBSP174392); 15♀, 13-17/II/2012, (IBSP 174396-174399) all collected by Equipe Carste; 1♂ 7♀, 12-24/IX/2011, J. Mascarenhas coll. (IBSP 174386–IBSP 174389, IBSP 174393).

###### Etymology.

The specific name is a noun in apposition taken from the type locality.

###### Diagnosis.

**Figure 14.**
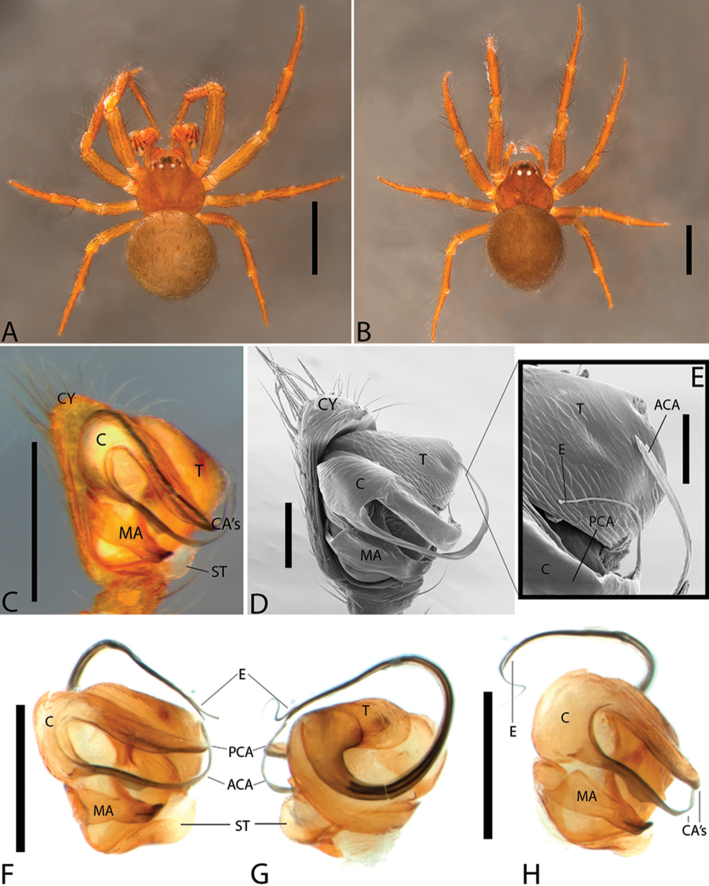
*Cuacuba morrodopilar* sp. n. **A** male, dorsal view **B** female, dorsal view **C** male palp, ventral view **D** male palp, ventral view (SEM) **E** detail of the lower conductor apophysis tip (SEM) **F, G, H** male palp, expanded (**F**, ventral view **G** dorsal view, **H**, retrolateral view). Abbreviations: **ACA** anterior conductor apophysis; **C** conductor; **CA** conductor apophysis; **E** embolus; **EA** embolic apophysis; **MA** median apophysis; **O** embolic opening; **PCA** posterior conductor apophysis; **ST** subtegulum; **T** tegulum. Scale bars: **A** 0.9; **B** 1.1; **C, F–H** 0.2; **D** 0.1; **E** 0.04 mm.

**Figure 15.**
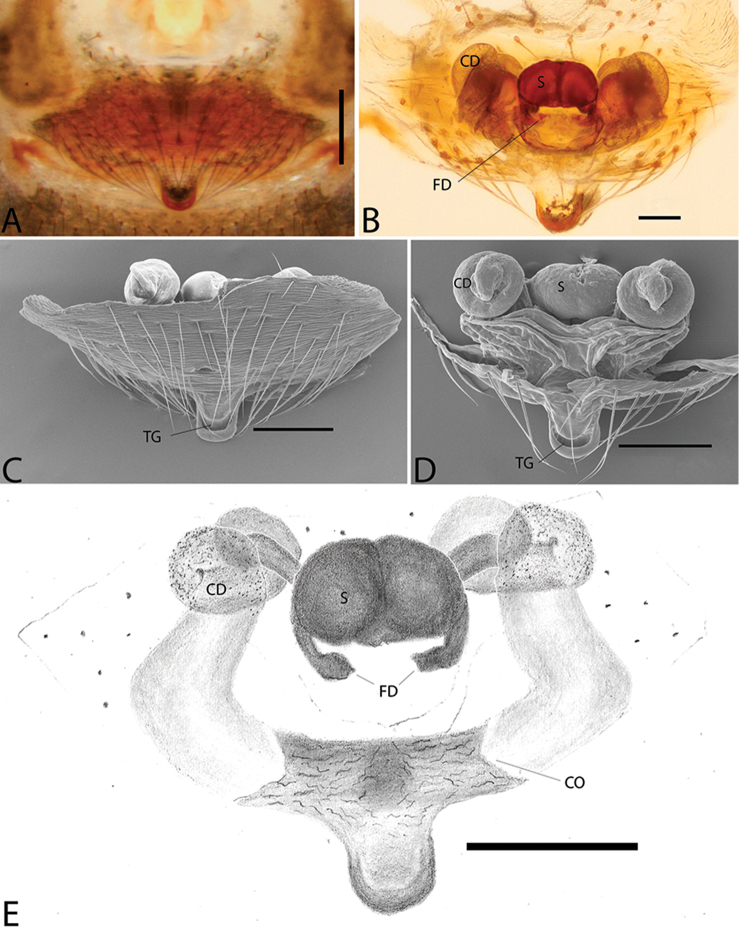
*Cuacuba morrodopilar* sp. n. **A** female epigynum, ventral view **B** female epigynum, dorsal view, clarified (LM) **C** female epigynum, ventral view (SEM) **D** female epigynum, ventral view with plate removed (SEM) **E** female epigynum, dorsal (drawing). Abbreviations: **CD** copulatory ducts; **CO** copulatory opening; **TG** transversal groove; **FD** fertilization ducts; **S** spermathecae. Scale bars: **A**, 0.9; **B** 0.13; **C, D** 0.1; **E** 0.25 mm.

Male of *C. morrodopilar* sp. n. differs from *C. mariana* sp. n. by the anterior conductor apophysis with flattened tip (Fig. 14D–E) and by the median apophysis with protruding and recurve tip, darkened (Fig. 14D, F, H). Females can be distinguished by the posterior margin of epigynal plate in “U” shape, with very remarkable distally notched transversal groove (Fig. 15A–E).

###### Description.

Male (holotype IBSP 174389). Cephalothorax and sternum brown. Chelicerae, endites and labium brownish. Legs light brown. Abdomen grayish. Total length: 1.68. Carapace: 0.91 long. Clypeus 0.18 high. Sternum 0.4 long, 0.33 wide. Eye measurements: AME: 0.06; ALE-PLE: grouped, 0.06 each; PME-PME: separated by 1 PME (0.06 each). Legs: I femur 0.77/ patella 0.35/ tibia 0.67/ metatarsus 0.48/ tarsus 0.44/ total 2.7. II 0.75/ 0.33/ 0.65/ 0.38/ 0.4/ 2.51. III 0.48/ 0.26/ 0.36/ 0.32/ 0.31/ 1.73. IV 0.63/ 0.28/ 0.46/ 0.35/ 0.3/ 2.02. Anterior conductor apophysis longer and thinner than posterior one (Fig. 14C–H). Abdomen: 0.81 long.

Female (paratype IBSP 174389). Coloration as in male. Total length: 1.8. Carapace: 0.9 long. Clypeus 0.18 high. Sternum 0.51 long, 0.48 wide. Eye measurements: AME: 0.07; ALE-PLE: grouped, 0.06 each; PME-PME: separated by 1 PME (0.07 each). Legs: I femur 1.07/ patella 0.45/ tibia 0.75/ metatarsus 0.61/ tarsus 0.48/ total 3.36. II 0.92/ 0.46/ 0.61/ 0.45/ 0.44/ 2.88. III 0.58/ 0.3/ 0.42/ 0.37/ 0.36/ 2.03. IV 0.76/ 0.3/ 0.54/ 0.44/ 0.35/ 2.39. Epigynal plate larger than long, with long bristles (Fig. 14A–D). Spermathecae connate (Fig. 15B, D–E). Abdomen: 1.24 long.

###### Variation.

2 males: total length: 1.3–1.7; carapace: 0.75–0.91; femur 1: 0.77–0.9; 10 females: total length: 1.78–2.8; carapace: 0.75–1.05; femur I: 1–1.25.

###### Distribution.

Known only from the type locality, Morro do Pilar, Minas Gerais, Brazil (Fig. 17).

**Figure 16.**
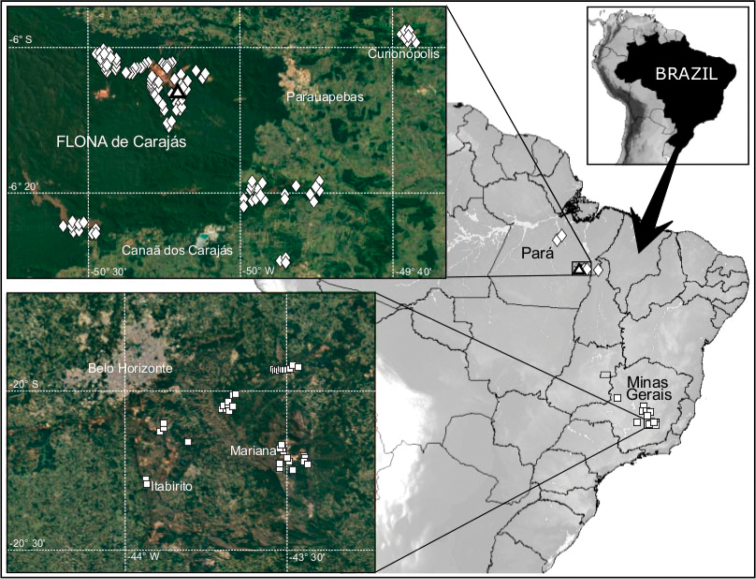
Distribution maps of *Plato ferriferus* sp. n. (diamond), *P. striatus* sp. n. (triangle) and *P. novalima* sp. n. (square) in states of Pará and Minas Gerais, Brazil.

**Figure 17.**
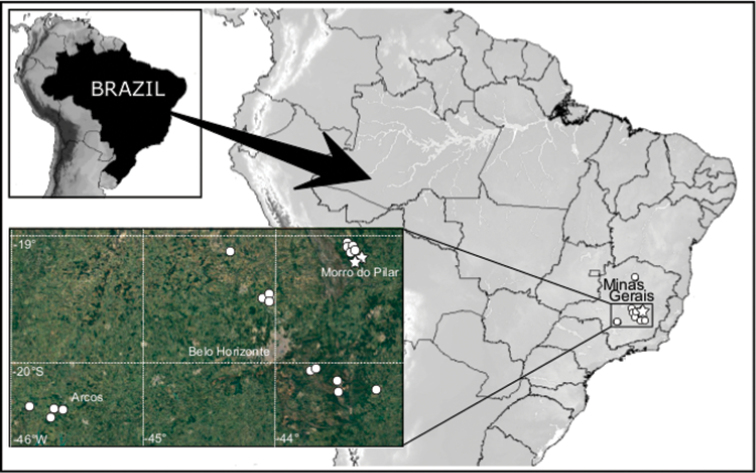
Distribution maps of *Cuacuba mariana* sp. n. (circle) and *C. morrodopilar* sp. n. (star) in state of Minas Gerais, Brazil.

## Discussion

### Relationships

Among the results obtained with the genus *Plato*, the three new species have the five putative synapomorphies indicated for Platoninae (see Coddington 1986: 18−19). A phylogenetic analysis is necessary to establish the relationships among all species of the genus. On the other hand, the peculiar characteristics of *Cuacuba* gen. n. genitalia make it difficult to insert the genus into any of the current four Theridiosomatidae subfamilies (Coddington 1986; Labarque and Griswold 2014). The same occurs even if we consider the two subfamilies proposed by Wunderlich (2011), Luangnaminae Wunderlich (with two tribes, and the genus type synonymized with *Coddingtonia* Labarque and Griswold 2014) and Theridiosomatinae Simon (with five tribes), a classification that was ignored by the recent authors.

*Cuacuba* gen. n. can be distinguished from the other genera of Theridiosomatidae by at least three putative synapomorphies, all located in the male palp: conductor C-shaped with a posterior apophysis covering the embolus (Fig. 11C–E), filamentous structures on the anterior apophysis (Fig. 9D) and cuneiform tegulum (Fig. 9B).

*Cuacuba* gen. n. is similar to the Asian species of the genus *Sinoalaria* Zhao & Li, 2012 in structures of the male genitalia. The long and thin embolus of *Cuacuba* gen. n. as well as the absence of an embolic division resemble the palp of the *Sinoalaria* males, which are also involved by a broad conductor. However, in *Sinoalaria*, the conductor does not present the peculiar C-shape (Figs 9A−B, 11C) nor the distal filamentous projections (Fig. 9D), which appear to be exclusive of *Cuacuba* gen. n.

As in *Plato*, *Cuacuba* gen. n. has a mesal embolic apophysis (Figs 1G, 12A) that differs it from the other genera of Theridiosomatidae. In the latter, the apophysis normally originates from the embolic division. Nevertheless, by their morphology, this mesal embolic apophysis does not appear to be homologous in *Plato* and *Cuacuba* gen. n. In addition, the palp of *Cuacuba* gen. n. resembles *Tagalogonia* Labarque & Griswold, 2014, *Coddingtonia* Miller, Griswold & Yin, 2009 and *Ogulnius* in the conductor covering the long and thin embolus.

The female genitalia in *Sinoalaria* resemble that of *Cuacuba* gen. n. in the route of the copulatory ducts and morphology of the connate spermathecae. However, differently from *Cuacuba* gen. n., the route of the copulatory duct seems to have little variation among the species of the former genus. *Sinoalaria bicornis* Lin, Li & Jäger, 2014 has the same route of the copulatory ducts as in *Cuacuba* gen. n. (see Lin et al. 2014: fig. 13E). The main difference between the female genitalia in the two genera is in the scape, as *Cuacuba* gen. n. has no scape, only a marked prolongation of the posterior region of the epigynal plate. Both genera have a transverse groove in the posterior area of the epigynum.

The female genitalia of *Ogulnius* and *Tagalogonia* also resemble those of *Cuacuba* gen. n. in the curves of the copulatory ducts. *Ogulnius* females have connate spermatheca, wide and sinuous copulatory ducts and a transverse groove. *Cuacuba* gen. n. (Figs 10A−B, 13D) has more complex curves in the copulatory ducts, more similar to those of *Tagalogonia*, besides presenting the posterior margin of the elongated and accentuated epigyinal plate. In addition, *Sinoalaria* females share the twisted routes of the coupling ducts with *Coddingtonia*, *Tagalogonia*, *Ogulnius*, Theridiosomatinae, and *Cuacuba* gen. n.

In general, the internal morphology of genitalia of Theridiosomatidae is conservative within each genus. The median spermathecae is present in most genera, usually paired, globular, connate or with little distance between them, in addition to coiled or enveloped ducts, peripheric to the spermathecae, generally varying more in width than length (Figs 2C, 6C, 13D, Coddington 1986: figs 26, 51, Labarque and Griswold 2014: figs 3F, 6F). Although *Sinoalaria* was well characterized by the authors, it has not been formally included in any subfamily. It was, however, considered a probable member of the Epeirotypinae or Platoninae subfamilies, due to the distal embolic opening and absences of apophysis and embolic division (Labarque and Griswold 2014).

*Cuacuba* gen. n. is very similar in external morphology and in general habits to *Plato*, so they can be easily confused (Pinto-da-Rocha 1995). In addition, some similarities in the genitalia are similar to *Sinoalaria*, *Tagalogonia* and *Ogulnius*. *Cuacuba* gen. n. resembles *Ogulnius* in the long and tapered embolus covered by the conductor, in addition to the female characteristics mentioned above; however, it does not present the diagnostic characteristics of Ogulninae Coddington, 1986, such as the abdomen greatly overlapping cephalothorax, leg IV longer than leg I and reduced body size. Important synapomorphies of Platoninae (probable subfamily to which *Sinoalaria* belongs) such as pointed cymbium, T-shaped paracymbium and median apophysis recurved with long-tip, are absent in *Cuacuba* gen. n. Thus, *Cuacuba* gen. n. shares general characteristics with different subfamilies and does not currently fit into any known subfamily. Without an extensive analysis of the intrageneric family relationships, the new genus cannot be allocated to any of the current subfamilies, nor can we confirm it is a sister group of *Sinoalaria*.

### Distribution and associated lithologies

Even with the development of biospeleology in Brazil, undersampled or even non-sampled areas (Trajano and Bichuette 2010) and large mappings of this fauna are extremely scarce. In this work, we identified 3868 adult spiders distributed in 1007 caves, and observed that the spider species in *Plato* and *Cuacuba* gen. n. are highly related to cave environments. They are troglophile aerial spiders that build orbicular webs but do not present troglomorphic characteristics. They mostly occur in dark environments and may occupy dysphotic environments, twilight regions and cave entrances. According to Coddington (1986), all described *Plato* species were located in cryptic habitats and/or in caves, corroborating the observed for species included in this work. This characteristic behavior was also observed for species of *Cuacuba* gen. n., which were sometimes confused with *Plato* (Pinto-da-Rocha 1995).

The species of *Plato*, *Baalzebub* Coddington, 1986 and *Cuacuba* gen. n. are the most frequent theridiosomatides in Brazilian caves. *Plato ferriferus* sp. n. is one of the spiders most commonly found in the ferruginous caves located in FLONA Carajás and surrounding regions, in the state of Pará. *Plato novalima* sp. n., and *Cuacuba mariana* sp. n., together with *Baalzebub acutum* Prete, Cizauskas & Brescovit, 2016 are the most frequently observed species in caves in the state of Minas Gerais. *Plato novalima* sp. n. is mainly found in the karst region of the Iron Quadrangle.

Due to the high abundance of individuals collected, we evaluated the frequency of capture of the species recorded in this study. We observed that the species of *Plato* are mostly located in caves in iron formations, whereas those of *Cuacuba* gen. n. are usually found in other lithologies, such as carbonates. *Plato ferriferus* sp. n., the most abundant species in this study (3098 adult spiders collected), was observed in 816 caves, 809 of which (99% of the records) were inserted in ferruginous rock outcrops. The sole representative of *Plato striatus* sp. n. was also collected in a ferruginous cave in FLONA de Carajás.

**Figure 18.**
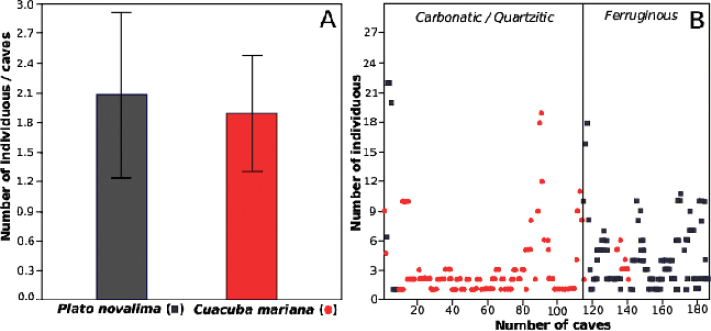
**A** Mean value of individuals collected per cave **B** Number of individuals collected per cave in their respective lithologies.

A more detailed analysis was performed on *Plato novalima* sp. n. and *Cuacuba mariana* sp. n., as they are the only species herein studied living in sympatry inside caves (n = 2) of the region of Iron Quadrangle in the state of Minas Gerais. *Cuacuba morrodopilar* sp. n. was collected only in two quartzite caves. Table 1 shows the number of individuals collected and from how many caves. For both species, the mean number of individuals collected per well was approximately two (Fig. 18A). However, when we evaluated the cave environment where the specimens were captured we observed that there is a preference for caves inserted in different lithologies (Fig. 18B).

**Table 1.** Number of individuals collected and number of caves studied per lithology. Key: *Capture frequency = N total individuals / N total caves by lithology.

Specie	Ferruginous caves	Capture frequency*	Limestone / Quartzitic caves	Capture frequency*
*Cuacuba mariana* sp. n.	33 ind. / 7 caves	0.45	312 ind. / 109 caves	3.00
*Plato novalima* sp. n.	306 ind. / 68 caves	4.19	85 ind. / 7 caves	0.82

## Supplementary Material

XML Treatment for Plato

XML Treatment for Plato ferriferus

XML Treatment for Plato striatus

XML Treatment for Plato novalima

XML Treatment for Cuacuba

XML Treatment for Cuacuba mariana

XML Treatment for Cuacuba morrodopilar
